# Single-Cell Transcriptomic Analysis of Kaposi Sarcoma

**DOI:** 10.1371/journal.ppat.1012233

**Published:** 2025-04-01

**Authors:** Daniel A. Rauch, Paula Valiño Ramos, Mariam Khanfar, John Harding, Ancy Joseph, Anam Fahad, Paul Simonson, Isabel Risch, Obi Griffith, Malachi Griffith, Lee Ratner

**Affiliations:** 1 Department of Medicine, Washington University School of Medicine, St Louis, Missouri, United States of America,; 2 Department of Molecular Microbiology, Washington University School of Medicine, St Louis, Missouri, United States of America,; 3 Department of Genetics, Washington University School of Medicine, St Louis, Missouri, United States of America,; 4 Department of Pathology and Laboratory Medicine, Weill Cornell Medical College, New York, New York, United States of America; University of Colorado Denver School of Medicine, UNITED STATES OF AMERICA

## Abstract

Kaposi Sarcoma (KS) is a complex tumor caused by KS-associated herpesvirus 8 (KSHV). Histological analysis reveals a mixture of “spindle cells”, vascular-like spaces, extravasated erythrocytes, and immune cells. In order to elucidate the infected and uninfected cell types in KS tumors, we examined twenty-five skin and blood samples from sixteen subjects by single cell RNA sequence analyses. Two populations of KSHV-infected cells were identified, one of which represented a *CD34*-negative proliferative fraction of endothelial cells, and the second representing *CD34*-positive cells expressing endothelial genes found in a variety of cell types including high endothelial venules, fenestrated capillaries, and endothelial tip cells. Although both infected clusters contained cells expressing lytic and latent KSHV genes, the *CD34*+ cells expressed more K5 and less K12. Novel cellular biomarkers were identified in the KSHV infected cells, including the sodium channel SCN9A. The number of KSHV positive cells was found to be less than 10% of total tumor cells in all samples and correlated inversely with tumor-infiltrating immune cells. T-cell receptor clones were expanded in KS tumors and blood, although in differing magnitudes. Changes in cellular composition in KS tumors after treatment with antiretroviral therapy alone, or immunotherapy were noted. These studies demonstrate the feasibility of single cell analyses to identify prognostic and predictive biomarkers.

## Author summary

Kaposi sarcoma (KS) is a malignancy caused by the KS-associated herpesvirus (KSHV) that causes skin lesions, and may also be found in lymph nodes, lungs, gastrointestinal tract, and other organs in immunosuppressed individuals more commonly than immunocompetent subjects. The current study examined gene expression in single cells from the tumor and blood of these subjects, and it identified the characteristics of the complex mixtures of cells in the tumor. In each tumor, two populations of KSHV infected endothelial cells were found, highly transcriptionally active and proliferative subsets, respectively. Both clusters included cells expressing lytic and latent KSHV genes. In addition, new biomarkers of KSHV infected cells were identified, such as voltage gated ion channel transcripts. The number of KSHV positive cells was inversely proportional to immune cell numbers in skin tumors. In addition, changes in the cellular composition were noted with therapeutic interventions, with elevated expression of genes associated with tumor infiltrating CD8+ T cells.

## Introduction

Kaposi sarcoma-associated herpesvirus (KSHV), which is also known as human gammaherpesvirus 8 (HHV-8), is a member of the rhadinovirus genus, and was first identified in 1994 [1]. The highly-conserved, circular, 165-kb double stranded DNA genome of KSHV has a 140-kb unique region encoding approximately 90 genes flanked by 20-30 kb of terminal repeat sequences [2]. The viral genome is maintained as an episome in infected cells and persists in a latent state during which it expresses a latency-associated nuclear antigen (*LANA, ORF73*), *Kaposin* (*K12*), *v-FLIP* (*K13*), *v-Cyclin* (*ORF72*), and 12 microRNAs (miRNAs) [3]. Induction of viral replication and lytic gene expression, often by inflammation, promotes the expression of the Replication Transactivation Activator (*RTA*, *ORF50*), and a resulting cascade of secondary and tertiary viral proteins that make the virus capsid and DNA synthesis enzymes.

The etiological agent of Kaposi sarcoma (KS), KSHV, exists in at least 5 subtypes [4] and is endemic in sub-Saharan Africa, parts of Eastern Europe, the Mediterranean, and parts of China, where rates can range from 30-90% [5]. In the U.S. and many high-resource nations the prevalence of KSHV infection is low in the general population, but substantially elevated in high-risk groups such as HIV-1 infected individuals who have sex with men and in immunosuppressed subjects. Saliva is the major route of KSHV transmission and in endemic regions of the world, most infections occur within the first 5 years of life [6]. KSHV is a class I carcinogen and about 1% of human tumors are associated with KSHV infection [7]. Fewer than 1% of immunocompetent KSHV infected individuals develop disease, however most immunosuppressed individuals infected with the virus manifest one (or more) disorders [8] including KS, multicentric Castleman disease (MCD), KSHV inflammatory cytokine (KICS), immune reconstitution syndromes (IRIS), and primary effusion lymphoma (PEL).

KS is an incurable disease originally described as a blood vessel tumor in 1872 by Hungarian Moritz Kaposi, and is now understood to be a highly vascularized solid tumor of endothelial origin, characterized by KSHV-positive “spindle cells”, cellular pleomorphism, an inflammatory infiltrate of lymphocytes and plasma cells, sinuous vascular spaces, extravasated erythrocytes, and fibrosis [9]. Forms of KS include classic KS (cKS), iatrogenic immunosuppression-associated KS (iKS), endemic KS (enKS), and epidemic HIV-1/AIDS-associated KS (epKS) [10]. The enKS and cKS are often indolent, whereas epKS can have widespread mucocutaneous, nodal, and visceral involvement [9]. Although the majority of infected cells in a KS lesion manifest KSHV in latency, lytic reactivation is a critical step in oncogenesis [11]. KSHV infection of endothelial cells or hematopoietic progenitors leads to changes in their morphology, glucose metabolism, proliferation, lifespan, and gene expression. KSHV oncogenicity is reflected by numerous pro-angiogenic molecules that are induced, including members of the vascular endothelial growth factor (VEGF)-VEGF receptor and angiopoietin families, Interleukin 6 (IL6) and IL8, and platelet-derived growth factor, through the activities of lytic proteins K1, K15, and viral G protein coupled receptor (*v-GPCR*) [12]. Latency proteins contribute to tumorigenesis through repression of apoptosis (*LANA, v-FLIP*), and activation of cyclin-dependent kinase (*v-Cyclin*). KSHV also elaborates an array of mediators of immune evasion [13].

Although recent transcriptomic studies have provided novel insights into latent and lytic KSHV genomic expression in tumors [14–18], questions remain that can only be addressed by a single-cell approach to transcriptomics. Given the cellular complexity, rarity of infected cells, and variable clinical course of KSHV associated disorders, single-cell analyses provide a unique opportunity to explore key interactions of lytic and latent infected tumor cells with the tumor microenvironment.

KS therapy is focused on disease palliation to improve quality of life and survival, but it is not curative [19]. A key management component is to minimize immune suppression, whether by reducing immunosuppressive medications for iKS, or optimizing antiretroviral therapy for epKS. For indolent localized KS with minimal cosmetic or functional disturbance, topical or localized therapies may be indicated. For aggressive or visceral disease, or lesions with moderate-severe cosmetic or functional disturbance, systemic therapy is indicated. This may include FDA-approved chemotherapies such as liposomal doxorubicin or taxanes, or the immune or cereblon-modulator drug (IMiD, cel-mod) pomalidomide. The mechanism of action of these drugs remains unclear but they are known to alter angiogenesis, cytokine production, and T-cell activation [20]. Other chemotherapeutic, anti-angiogenic, proteasome inhibitor, and immune checkpoint inhibitor drugs showed preliminary activity. However, response rates of 30-60% are seen with most approaches, and biomarkers of activity remain to be defined. In rare cases, exacerbation of KS-associated inflammatory disorders were seen [21].

Previous studies suggested that KSHV latent and lytic gene expression occurs in KS, and disruption of either program results in tumor regression [22]. Animal models for KS are lacking, and there is a dearth of genomic studies on primary KS tissue due to the admixture of multiple cell types, the small proportion of KSHV positive cells, and the complexity of fibrotic skin tumors. Here we used a scRNAseq multiomic approach to characterize the cellular and viral KS transcriptome at a single cell level in primary tissue. These findings may have applications for discovery of prognostic and predictive biomarkers and therapeutic insights for the design of safe and effective therapies for KS. Application of these technologies to understand primary KS pathogenesis and therapeutic responses may be applied to understanding oncogenic virus biology, as well as defining the evaluation and treatment of other infection-associated cancers.

## Results

Sixteen participants contributed twenty-five samples for scRNAseq analysis, comprising nineteen KS skin biopsies, one non-KS skin biopsy, and five PBMC samples (Table 1). Eleven HIV+ participants had AIDS-associated KS (epKS), two HIV- participants had classic KS (cKS), one HIV- participant had iatrogenic KS (iKS), one HIV+ participant who also had a renal transplant had KS (ep/iKS), and one HIV+ participant had neither KS nor AIDS. Three participants contributed samples before and after therapy, one treated with nivolumab and ipilimumab, one treated with antiretroviral therapy, and one treated with pomalidomide. The participants with classic KS were the only females in the cohort. The sample set is small, diverse, and representative of the patient population presenting for clinical care in a non-endemic region. The current study is intended to demonstrate the feasibility and reproducibility of the methodology, generate hypotheses for future studies, and offer novel insights into potential biomarkers, pathways, and therapeutic targets.

**Table 1:** Kaposi Sarcoma Primary Patient Samples.

Subject	Age	Sex	Race^1^	Ethnicity^2^	HIV-1 Status	Subtype	CD4 count (cells/ul)^3^	CD8 count (cells/ul)^4^	HIV-1 RNA Level in Plasma (copies/ml)	Additional Samples^5^	Number of Cells, PBMC (CellRanger)	Number of Cells, Skin (CellRanger)	Percent KSHV^+^ (>1 read) Cells, Skin
KS1	37	M	B	N	POS	AIDS ASSOCIATED	A: 108 B: 156^7^	A: 690 B: ND	<20	–		A: 16938 B: 23497	A: 0.44 B: 0.28
KS2	26	M	B	N	POS	AIDS ASSOCIATED	139	754	<20	–		15537	1.16
KS3	34	M	W	H	POS	AIDS ASSOCIATED	<35	ND	<20	–		6730	1.35
KS4	32	M	B	N	POS	AIDS ASSOCIATED	<35	ND	1492	–		26028	0.01
KS5	54	M	B	N	POS	NOT KS^6^	444	ND	<20	–		2136	0^12^
KS6	34	M	B	N	POS	AIDS ASSOCIATED	A: 203 B: 347^8^	661	<20	PBMC-A PBMC-B ^10^	A: 48618 B: 5146	A: 17168 B: 21742	A: 1.25 B: 3.76
KS7	81	F	W	N	NEG	CLASSIC	>2000	ND	ND	–		A:5348 B: 3249^11^	1.07 0.86
KS8	35	M	B	N	POS	AIDS ASSOCIATED	95	ND	52	–		13258	7.17
KS9	31	M	B	N	POS	AIDS ASSOCIATED	<35	ND	86	–		5546	8.65
KS10	61	M	B	N	POS	AIDS ASSOCIATED	A: 225 B: 152^9^	ND	210	PBMC-B	16078	A: 5987 B: 19301	A: 0.4 B: 1.09
KS11	76	M	W	N	POS	IATROGENIC	114	ND	<20	PBMC	8635	1033	5.81^13^
KS12	73	M	B	N	NEG	IATROGENIC	N/A	ND	ND	PBMC	16407	4508	1.09
KS13	90	F	W	N	NEG	CLASSIC	629	ND	ND	–		6396	0.64
KS15	32	M	W	N	POS	AIDS ASSOCIATED	141	956	11,400	–		5207	1
KS16	32	M	B	N	POS	AIDS ASSOCIATED	<35	ND	160,000	–		12155	0.92
KS18	50	M	B	H	POS	AIDS ASSOCIATED	562	ND	<20	–		7673	0.14

ND: not done

Samples were obtained from sequential patients from the central U.S. presenting for medical care, excluding KS14 described elsewhere and KS17 for whom inadequate number of viable cells were obtained

^1^Race: B, black; W, white

^2^Ethnicity: H, Hispanic; N, non-Hispanic

^3^Reference Range 365-1294

^4^Reference Range 187-781

^5^PBMC: peripheral blood mononuclear cells

^6^Diagnosis on skin biopsy: Dermal sclerosis and mixed inflammation with negative KSHV immunohistochemical stain

^7^At time of 2^nd^ skin biopsy after 4 weeks of treatment with nivolumab plus ipilimumab

^8^At time of 2^nd^ skin biopsy after 7 months of antiretroviral therapy

^9^At time of 2^nd^ skin biopsy after 21 days of pomalidomide therapy

^10^PBMC samples obtained at the time of both skin biopsies

^11^This sample B constitutes a duplicate submission of the same skin biopsy. No 2^nd^ skin biopsy was obtained.

^12^This sample was excluded from analysis due to non-KS diagnosis (see footnote^6^)

^13^This sample was excluded from analysis due to poor quality (low fraction of confidently mapped reads)

Cells utilized for scRNAseq were obtained from viably frozen single-cell suspensions prepared from fresh primary skin and blood samples and submitted in three batches for 10X Genomics 5’ or 3’ gene expression with multiomic TCR profiling (S1 Fig). KSHV transcripts could be detected in all KS skin tumors. HIV-1 and EBV infected cells were not detected in KS lesions. The number of cells analyzed by scRNAseq and the percent KSHV positive cells for each sample (S1C Fig) are indicated in Table 1. Comparisons to scRNAseq of normal skin utilized published data. (S2 Fig) [23].

The rarity of KSHV transcript reads in infected cells and the rarity of infected cells in tumors resulted in an analysis challenge in which stringent quality control measures typically applied to filter out noise (cells that expressed less than 100 features; genes that were expressed in less than 10 cells) also filtered out most KSHV-infected cells. Filters for dying cells (>20% mitochondrial genes), low-quality reads (proportion of UMI > 93^rd^ percentile), and doublets removed between 15 and 25% of cells from each skin sample and an average of 32% of cells from PBMC samples. For identifying KSHV+ cells in skin samples, most suspected false-positives resulted from 1 KSHV read per cell. Evaluating cells with more than 1 KSHV read per specified gene retained 70% of suspected true positives and removed ~90% of suspected false positives (S3 Fig).

### The landscape of primary KS

**Fig 1:**
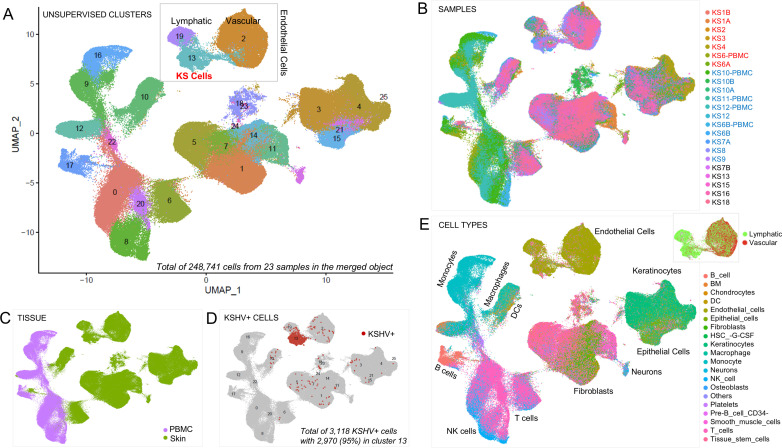
Landscape of Primary KS. Single cell suspensions from viably frozen primary KS blood and enzymatically separated skin tumor samples processed for scRNAseq resulted in A) an unsupervised UMAP clustering projection of the merged data set containing 248,741 cells from 23 samples representing a landscape of 25 clusters. Between 15 and 25% of cells from each sample were removed when filtering out dying cells (>20% mitochondrial genes), low quality cells (proportion of UMI > 93^rd^ percentile), and doublets (identified by DoubletFinder package). Seurat’s default settings were used for normalization and scaling. Principal component (PC) analysis was based on 22 high variable genes with clustering of cells set at a resolution of 0.6. The box around clusters 2, 13, and 19 indicates they are endothelial cells in skin tumors with 95% of KSHV-infected cells mapping to cluster 13. B) UMAP clustering projection color coded based on individual sample distribution across the merged dataset. Samples were submitted in three batches indicated by the color of the sample name in the legend (batch 1, red; batch 2, blue; batch 3, black) and the percent of KSHV+ cells contributed by each sample and the percent of KSHV+ cells present in each sample is shown in S1C Fig. C) UMAP clustering projection color-coded based on tissue source of each sample demonstrated that cells obtained from skin (n=175,472) and peripheral blood mononuclear cell (PBMC) (n=73,269) form clearly delineated clusters. D) UMAP clustering projection color-coded based on reads corresponded to the KSHV genome in which 3,118 KSHV+ cells (with 2 or more reads of viral transcripts) in the merged object corresponding to an average of 1.8% of total tumor cells. 2,970 (95%) of KSHV+ cells were in cluster 13. KSHV+ cells were not detected in PBMC samples. E) UMAP clustering projection color-coded were based on cell annotation using SingleR and the Human Primary Cell Atlas (HPCA) reference dataset. The inset focuses on vascular (red) vs. lymphatic endothelial cells (green) within clusters 2, 13, and 19 identifying cluster 13 as predominantly lymphatic endothelial cells.

To observe the cellular landscape of primary KS, 248,741 cells from twenty-three samples (5 peripheral blood mononuclear cell, PBMC, samples and 18 skin samples submitted in three batches; S1 Fig) were merged into a single dataset, forming 25 unsupervised clusters (Fig 1A). Results for each of the individual samples are highlighted in Fig 1B. Cells from the peripheral blood (n=73,269) were clearly distinct from those in the skin samples (n=175,472) (Fig 1C). In addition to human transcripts, expressed KSHV genes were also detected in 3,118 cells (1.8% of total skin tumor cells), exclusively from skin samples (Fig 1D). In the merged plot, 95% of KSHV-expressing cells clustered together in cluster 13 and migrated very closely to, though separate from, non-infected endothelial cells in clusters 2 and 19.

In the peripheral blood, separable clusters of cells were found for monocytes, B and T lymphocytes, and natural killer cells (Fig 1E). Within skin biopsies, several clusters of endothelial cells and fibroblasts were detected as well as keratinocytes and epithelial cells. Clusters of B and T cells, macrophages, and dendritic cells were also detected in skin preparations and, interestingly, formed distinct clusters from the same cell types obtained from peripheral blood, though they migrate closely.

#### Two populations of KSHV infected cells in primary KS skin lesions.

**Fig 2:**
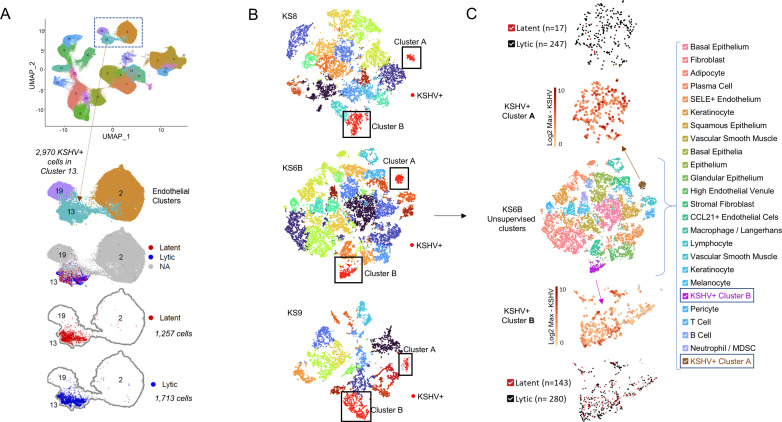
Latent and Lytic KSHV in Primary KS. A “latent” cell is defined as any cell expressing at least two reads from any of the following KSHV latency transcripts: *LANA (ORF73, gp81), Kaposin (K12, gp79), vFLIP (ORF71, gp80), K15 (ORF75, gp85), vOX-2 (K14, gp83), or vIRF-2 (gp65)* AND expressing no other KSHV gene. A “lytic” cell is defined as any cell expressing at least one read from a latency transcript AND at least one read from any other viral gene. When all samples are combined in a single data set as in Fig 1, KSHV infected cells populate in a single cluster (cluster 13). A) Lytic vs. Latent: Within the UMAP clustering projection described in Fig 1, 2,970 KSHV+ cells are in cluster 13; of which the virus is in latency in 1,257 cells (42%) and lytic replication in 1,713 cells (58%). However, when samples are evaluated individually, unsupervised clustering divides KSHV infected cells into two distinct clusters. B) Two clusters: 10X Cell Ranger, unsupervised, graph-based t-SNE plots of KS6B, KS8, and KS9 skin tumors with KSHV+ (red) clusters (A and B) highlighted. Remaining cluster colors are not coordinated between samples. C) An exploded diagram of the t-SNE plot of sample KS6B in which clusters A (brown) and **B** (purple) are magnified and color coded based on Log2 KSHV gene expression and lytic vs latent cell identity demonstrate that viral gene expression and lytic vs latent replication are not the defining features that distinguish the cluster A from cluster B. Samples shown in panels B and C were those samples with >2% KSHV-positive cells (S1C Fig).

**Fig 3:**
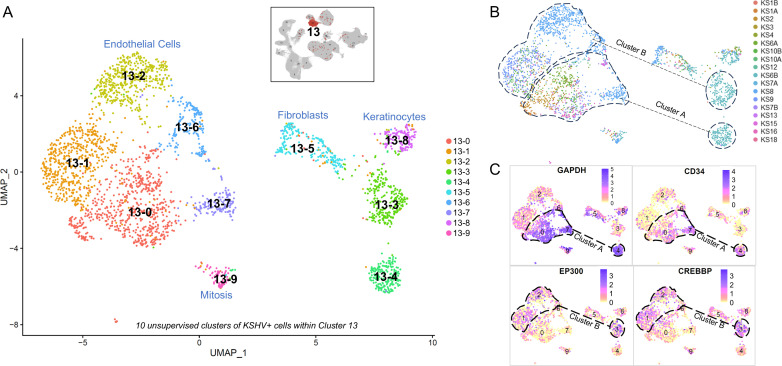
Two Populations of KSHV Infected Cells in Primary KS. To characterize infected tumor cells, a new unsupervised analysis of the 2,970 KSHV+ cells within cluster 13 was performed (with number of principle components = 10 and resolution = 0.3) in which cells were annotated using SingleR and Human Primary Cell Atlas (HPCA) dataset resulting in A) a UMAP projection of 10 KSHV+ clusters (numbered 13-0 through 13-9). Although cells in cluster 13 were identified as lymphatic endothelial, small subclusters of KSHV+ cells also express keratinocyte markers (cluster 13-8, n=19 of 135), fibroblast markers (subcluster 13-5, n=23 of 204), vascular endothelial markers (subcluster 13-6, n=40 of 182), or markers of mitosis, including MKI-67, RRM2, and DIAPH3 (subcluster 13-9, n=76). B) The remaining subclusters can be divided into two groups with subclusters 13-1, 13-2, and 13-3 representing cluster B from Fig 2 and subclusters 13-0, 13-4, 13-7 representing cluster A. A UMAP projection color-coded based on sample reveals that subclusters 13-3 and 13-4 are exclusively from sample KS6B and subclusters 13-2 and 13-7 are exclusively from KS8. C) A FeaturePlot of Log2 expression values representing normalized read counts for *GAPDH, CD34, EP300* and *CREBBP* identify cluster A (subclusters 13-0,4,7) as *CD34*^+^GAPDH^HI^ and cluster **B** (subclusters 1,2,3) as *CD34*^-^GAPDH^LO.^

Although in the composite object KSHV infected cells were clustered in a single cluster (Figs 1,2A,S1D), in individual KS skin tumors, cells in which KSHV genes were expressed typically formed two distinct clusters (Figs 2B,2C,3,S4 and S5B-D). Both clusters contained cells expressing lytic and latent KSHV genes (Figs 2C,S5B and S5D). Both clusters expressed endothelial markers *PECAM1* (*CD31*), *FLT4 (VEGFR3),* and *CD36*; lacked *ITGA2B, PDGFRA*, and *ACKR4*; and expressed the vascular-to-lymphatic endothelial determinant prospero homeobox protein 1 (*PROX1)* (S5A Fig) [24]. However, one of the two KSHV-infected clusters (cluster A) was highly transcriptionally active (S5B,S5C,S5E and S5G Figs). Thousands of host genes were differentially expressed in this cluster, including *CD34* and, unexpectedly, housekeeping genes including *GAPDH* (Figs 3C, S2B,S2D,S4 and S5).

Expression of *CD34* and lymphatic vessel endothelial hyaluron receptor 1 (*LYVE1)* in this cluster, along with ribosomes, exosomes, and markers like *LGALS1, IGFBP7, CD63*, and *BSG* was consistent with genes expressed in high endothelial venules (HEVs) (S5E Fig) [25]. However, while cell types like telocytes can be excluded (due to expression of *CD31*, *PDPN*, and *LYVE1*, and the absence of *PDGFRA),* the expression of markers for fenestrated capillaries (*PLVAP*) and endothelial tip cells (*NID2, ITGB1, CAV1, GMFG, IGFBP3, TGFBI, KLF2, PLAUR*, *SOX17*) makes the identity of the *CD34*+ cells unclear [25–28]. Interestingly, the *CD34*+ KSHV+ cluster expressed transcripts associated with spliceosome and electron transport machinery, and also exhibited high expression of lymphocyte antigen 6 complex, locus H (*LY6H*), which has been described by Moorad et al. as associated with “inflammatory” KS lesions (S5F Fig) [15]. Although *CD34*+ and *CD34*- endothelial cells were also seen in normal skin samples, there were no significant differences in GAPDH expression (S2 Fig).

In the *CD34*- cluster, housekeeping genes were suppressed while cellular proliferation factors were enriched including *EP300* and *CREBBP* (S4 and S5D Fig), along with factors like *DTX1, DTX4, HEY1*, and *CTNNB1* that regulate the *NOTCH* and *WNT* signaling pathways. Expressing biomarkers consistent with a lymphatic endothelial lineage (S5A Fig), the *CD34*- cluster is likely representative of proliferating KS cells. Despite the significant differences in viral, cellular, and specific biomarker gene expression, the two infected clusters were grouped together in our merged UMAP plot, supporting similarities between these 2 clusters that could be due to a common endothelial cell lineage origin. KSHV+ cells with characteristics of both clusters could be detected in skin tumors from all KS samples with >50 KSHV+ cells (S4A-O Fig), both may be involved in tumor growth, and each cluster represents a largely uncharacterized and rare sub-population of cells within KS tumors (Figs 1D and S1C) that are readily distinguishable by expression of housekeeping genes.

#### Novel biomarkers of KSHV infected cells in primary KS lesions.

**Fig 4:**
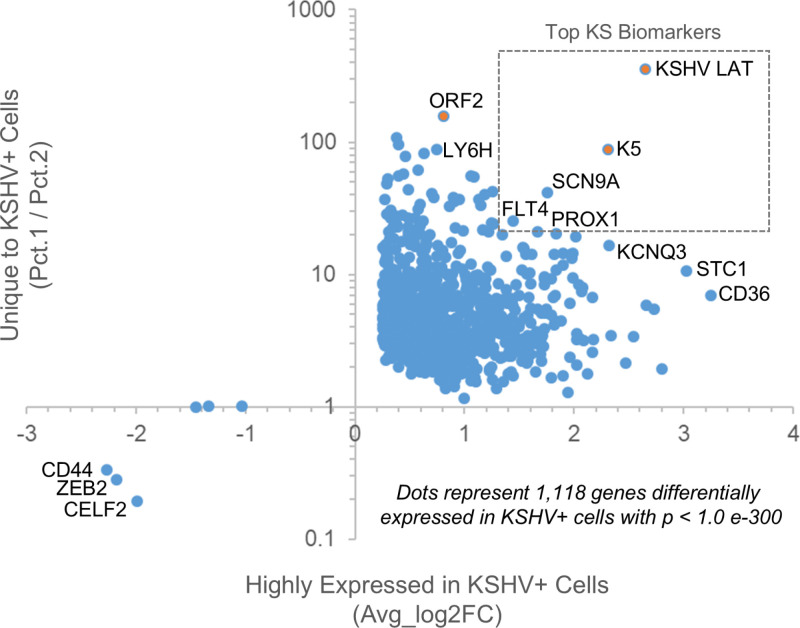
Differential Expression Analysis of KSHV **+** **cells.** A) Differential expression analysis of genes expressed in KSHV+ cells included 1,118 genes with a p value less than 1e-300. When graphed as the average Log2 fold change of gene expression in KSHV+ vs. KSHV negative cells against the ratio of percent of KSHV+ cells expressing that gene (Pct.1) to the percent of KSHV- cells expressing that gene (Pct. 2) the KSHV latency cluster downstream of the LTd promoter (designated *KSHV LAT*, including viral genes *v-FLIP, v-CYC, and K12*) along with the lytic transcript *K5* were the top viral biomarkers. The voltage-gated sodium channel *SCN9A (Nav1.7)* emerged as the top, non-KSHV biomarker among a number of previously described factors including *FLT4, PROX1, STC1* and *CD36*.

In addition to viral genes, several cellular genes were commonly expressed in the KSHV positive cells including *PROX1*, mannose receptor C-type 1 (*MRC1; CD206*), fms-related tyrosine kinase 4 (*FLT4*), and *Kir2.1* inward-rectifier potassium channel (*KCNJ2*) (S5A Fig). These markers were previously described in KS lesions and provide confirmation for the sensitivity and reproducibility of scRNAseq data obtained from primary skin lesions [29–31]. Differential expression analysis revealed 1,118 significantly enriched genes in KSHV+ cells including several voltage gated sodium channel (VGSC) genes with *SCN9A* as a top biomarker candidate along with the KSHV Latency cluster (Figs 4, S1D, S6, S7 and S8). The expression of the VGSC gene *SCN9A* has not been previously described in KS, was tightly associated with both clusters of KSHV infected cells (S7A-C Fig), was minimally expressed in uninfected endothelial or other stromal cells, and was not expressed in the KS-negative skin samples (S2 Fig).

#### The number of KSHV+ cells is inversely proportional to immune cell number in primary KS.

**Fig 5:**
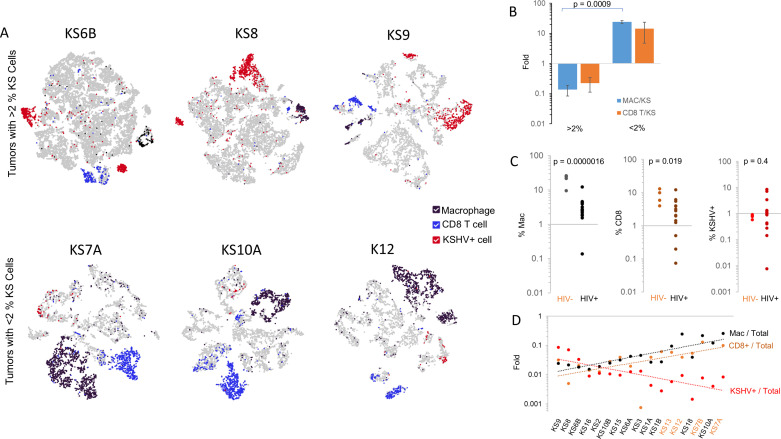
Immunity in Primary KS. A) t-SNE plots of primary KS tumor samples in two groups, three samples with greater than (>) 2% KSHV positive cells (KS6B, KS8, KS9) and three samples with less than (<) 2% KSHV positive cells (KS7A, KS10A, KS12) color- coded to highlight Macrophages (Black dots; defined as all cells with elevated Log2 sum expression >2 for *CD14, CD80, CD86, TLR2, MARCO, IL10, IL1B, IL1A*, and *ITGAX* (*CD11C*) also see S9 Fig), CD8+T cells (Blue dots; defined as all cells with elevated Log2 sum expression >2 for *CD2, CD3E, CD8A, NKG7, LAG3, GZMH, PTPRCAP*, and negative for *CD4*), and KSHV+ tumor cells (Red dots, defined as all cells expressing KSHV genes). All samples with >2% KSHV-positive cells are shown, and samples shown with <2% KSHV-positive cells are representative of the entire group of samples with that characteristic. B) Graph representing the ratio of Macrophages to KSHV+ cells (black bars), and CD8+T cells to KSHV positive cells (blue bars) in samples are shown in panel A. C) Percent Macrophages, Percent CD8+ T cells and Percent KSHV+ cells in samples from HIV+ vs. HIV- donors with p values shown for comparisons of the results from each group. D) Dot plot of the ratio (Fold) of Macrophages/ Total; CD8+ T cells/ Total; and KSHV+ cells/ Total, for each skin sample. Correlation coefficient for Macrophage vs. KSHV = -0.273 (1 tailed p value = 0.14); CD8+T cell vs. KSHV = -0.356 (one tailed p value = 0.074); Macrophage vs. CD8+ T cell = 0.69 (one tailed p value = 0.00007).

In most samples, including two HIV-negative samples, KSHV+ cells were very rare, comprising less than 2% of the total cells in the sample (Figs 5A and S1C). In three samples, all of which were HIV-associated, KSHV+ cells were notably more abundant, representing 3-9% of the total cells in the sample. In samples in which KSHV+ cells were rare, macrophages (S9 Fig) and CD8+ T lymphocytes were more abundant than in samples in which KSHV+ cells were prevalent (Fig 5). In these samples there was an inverse correlation between the number of macrophages and CD8 T cells and the number of KSHV+ cells in the primary tumor (Fig 5D) suggesting their role in KS immunity. HIV-negative samples had significantly more CD8 T cells and macrophages than samples obtained from AIDS-associated lesions (Fig 5C).

### The peripheral blood of KS subjects

Whether truly absent or simply below the level of detection for scRNAseq, there were few EBV+, HIV-1+, or KSHV+ cells in the peripheral blood mononuclear cells (PBMCs) (S10 Fig). However, the data did reveal unique characteristics of T cells in the peripheral blood of KS patients.

#### Low CD4:CD8 ratio in peripheral blood in HIV+ KS subjects.

**Fig 6:**
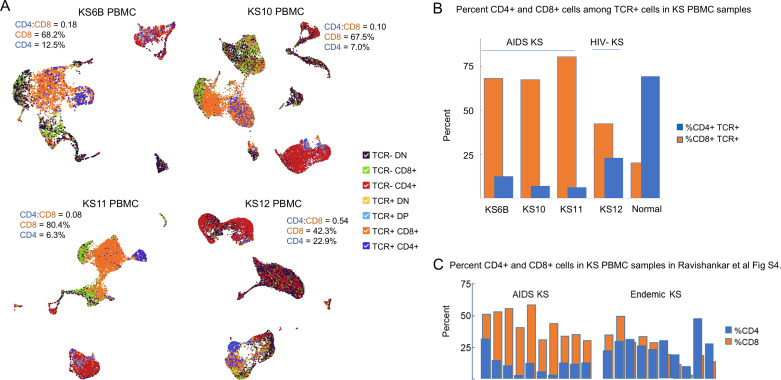
Low CD4:CD8 Ratio in KS Blood. A) UMAP plots for 4 primary peripheral blood mononuclear cell (PBMC) samples in which multiomic scRNAseq and T-cell receptor (TCR) sequencing was performed (for batch 1 samples including KS6A PBMC, TCRseq was not performed). The combination of TCR reads with gene expression for CD4 and CD8A clearly defined clusters representing the following cell types: TCR+CD4+ (Blue; CD4+ T cells), TCR-CD4+ (Orange; monocytes) also see S9 Fig, TCR+CD8- (Green; CD8+ T cells), TCR-CD8+ (Red; NK), TCR+DP (Purple; double-positive T cells), TCR+DN (Brown; double-negative T cells). The ratio of CD4+ T cells to CD8+ T cells is indicated, as well as the percentage of CD4+ and CD8+ T cells out of total TCR+ cells. B) Quantitation of the percent CD4+ TCR+ and percent CD8+ TCR+ cells in KS peripheral blood mononuclear cell (PBMC) samples (three AIDS-KS and one HIV-negative iatrogenic KS sample are compared to publically available PBMC data from a normal reference data set from ScaleBio [80]. C) Data reported in S4 Fig of Ravishankar *et al*. [33] in which the CD4:CD8 ratio in AIDS-KS is similar to the samples shown in panel B.

The CD4:CD8 T cell ratio is an important biomarker of pathogenesis. In healthy subjects the CD4:CD8 T cell ratio in whole blood is usually greater than 1.0, indicating that CD4+ T cells are typically present in greater abundance than CD8+ T cells [32]. In our PBMC samples, the CD4:CD8 T cell ratio was less than 1 (Fig 6), with all four of these KS patients being immune-suppressed due to AIDS or immunosuppressant medication. For the HIV+ subjects, the low ratio CD4:CD8 T cell ratio resulted from both low representation of CD4 T cells (6.3-12.5% of total PBMC sample) and notably high representation of CD8 T cells (67.5-80.4% of total PBMC cells). While PBMC counts of CD4+ and CD8+ T cells based on transcript counts are not comparable to whole blood reference values based on cell surface protein expression, these proportions could be reflective of these subjects’ persistent HIV infection [32]. The KS12 blood sample from the HIV negative iKS participant had the highest CD4:CD8 T cell ratio, albeit still <1, due to a low proportion of both CD4+ T cells (22.9% of total cells) and CD8+ T cells (42.3% of total cells). Similar results were described in Ravishankar et al (Fig 6C) [33]. In calculating these ratios, the value of combining scRNAseq with TCR sequencing was apparent, clearly distinguishing TCR-CD4+ monocytes (S9B Fig) from TCR+CD4+ T cells, and TCR-CD8+ NK cells from TCR+CD8+ T cells [33–35].

#### Expansion of T cell clones in peripheral blood of KS subjects.

**Fig 7:**
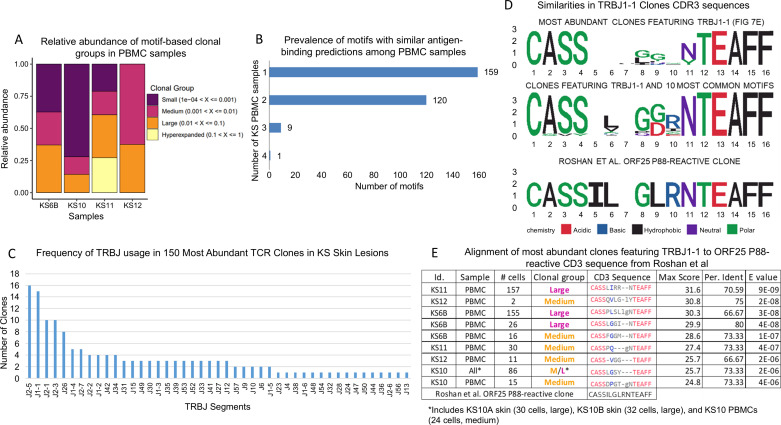
Characterization of T cell diversity in KS. A) Diversity of T cell clonal expansion in peripheral blood mononuclear cell (PBMC) samples from 4 patients, characterized by proportion of space occupied by clones. Clonal expansion was ascribed to one of 4 relative abundance categories, based on a clone size score calculated by scRepertoire. Clonal size cut points are listed in the panel legend [81]. B) Number of samples expressing the most common complementarity-determining region 3 (CDR3) motifs found in GLIPH2 clustering (Grouping Lymphocyte Interactions by Paratope Hotspots). Among 289 motifs, only one was found among PBMC samples from all 4 subjects. C) The graph depicts the frequency of T-cell receptor β chain joining segment (TRBJ) usage in the 150 most abundant T-cell receptor (TCR) clones in primary KS samples with matched blood and skin samples (KS6B, KS10, KS11, KS12). D) Three sequence logos; top) 9 CDR3 sequences in abundant TRBJ1-1 clones in KS PBMC samples from 4 patients described in panel E; middle) 19 CDR3 sequences from TRB1-1 clones found by GLIPH2 clustering described in panel B; compared to bottom) the anti-open reading frame (ORF) 25 P88 sequence reported by Roshan et al. in [40]. The y-axis BITS indicates the height method used by the sequence logo generation package. E) Alignment of 9 CD3 TRBJ1-1 sequences from abundant T cell clones from 4 KS subjects to the sequence reported in [40]. ‘Max score’ refers to the highest bit score, or sequence similarity score, for the alignment. ‘Perc. Ident’ refers to the percent of total sequence amino acids that are identical to the Roshan et al. sequence [40]. ‘E value’ refers to the significance score. Similar to p values, E values closer to zero represent fewer chances of a result being due to chance, and hence higher significance. The sequence color code refers to the E values, with gray marking an alignment score of <40, blue marking a score of 40-50, and red marking identical residues (score >200) [82]. Samples shown in panels C-E are all samples for which TCR analysis was performed on both skin and PBMCs.

T cells present in the peripheral blood exist as a unique oligoclonal pool of various T cell clones carrying a diverse array of antigen-specific T cell receptors (TCRs) [36]. An emerging challenge in tumor immunology is elucidating the role and identity of tumor or pathogen specific T cell clones. By combining single-cell TCR profiling with single-cell gene expression, multiplex scRNAseq provides a powerful tool to identify KS-specific CD8+ TCR clones [37]. In nine samples from four participants for whom TCR reads from both tumor and PBMC could be evaluated, medium to large clonal groups (with 0.1-10% abundance) were present in PBMCs (Fig 7A) and the distribution of VDJ recombination was non-random among the most abundant CD8+ clones in skin tumors (Fig 7C). One complementarity-determining region 3 (CDR3) sequence motif pattern was found in all 4 PBMC samples, 9 motifs were found in 3 of 4 PBMC samples, and 120 motifs were shared between two PBMC samples (Fig 7B). The most common motifs and the clones that endorse them are listed in S1 Table. Identical CD8 T cell clones were found in both the skin and the peripheral blood from the same patient and in two different skin samples taken before and after therapy from the same patient (Figs 7E and S11). Interestingly, in these cases, the most abundant T cell clone in the peripheral blood was not the most abundant clone in the tumor. The most abundant T cell clones among the KS samples carried a subset of frequent rearrangements, especially enriched in TCR β joining region 1-1 (TRBJ1-1) and TRBJ2-5, with recurring similarities among the CDR3 sequences of the most abundant clones in each patient. Interestingly, TRBJ1-1 and TRBJ2-5 were not the most frequently used TRBJ variants in the TCRβ repertoire of healthy controls [33,38,39], and the CDR3 sequences associated with TRBJ1-1 clones shared significant similarities with the CDR3 sequence (CASSILGLRNTEAFF) found in CD8+ T cell clones that react with the KSHV major capsid protein (ORF25) (Fig 7D and 7E) [40].

### Tumor-associated KSHV single cell transcriptome

#### Detection of lytic and latent KSHV gene expression in primary KS skin lesions.

In primary KS lesions, only a small subset of cells was positive for KSHV. KSHV gene expression is tightly regulated in infected cells and the virus persists in lytic and latent phases of replication and dormancy [3]. During latency, the virus is largely quiescent, and expresses a small subset of viral genes from the KSHV “latency cluster” of genes including *LANA* (ORF73; gp81), *Kaposin* (K12; gp79), *vFLIP* (ORF71, gp80), and *vCYC* as well as other latency genes including *K15* (ORF75, gr85), *vOX-2* (K14, gp83), and *vIRF-2* (gp65). Cells in which any or all of these genes were expressed, but not other KSHV genes, were considered to be harboring latent virus. Cells in which latency genes together with other KSHV genes were expressed were considered to be harboring virus in lytic replication. The use of scRNAseq to quantitate gene expression from the KSHV latency cluster is difficult due to the polycistronic nature of transcripts [41]. The LANA, *v-CYC*, and *v-FLIP* genes can all be co-transcribed into a single mRNA originating from the constitutive latency (LTc) promoter (S6 Fig). Alternatively, transcripts originating from either the LTc promoter or the downstream latency (LTd) promoter can include *v-CYC* and *v-FLIP* without including *LANA*. Spliced transcripts originating from the LTd promoter can also express the *Kaposin* gene. Using IGV to visualize Bam files from scRNAseq reads mapped to the KSHV genome it is apparent that the LTd promoter is the predominant promoter in the KSHV Latency cluster in vivo and that transcripts of *v-CYC, v-FLIP* and *Kaposin* are much more abundant than transcripts containing *LANA* in primary lesions [41]. Viral genes regulated by the LTd promoter were the most highly expressed latency genes (designed *KSHV LAT*), and *K5* was the most highly expressed lytic gene (Fig 4). One sample, KS6B, may have an amplification of the portion of the viral genome encoding *K5-K7*, based upon the high levels of these viral transcripts in this sample (S12A and S12B Fig). Amplification of this region of the KSHV genome has been described in approximately one-third of virus samples harvested from primary KS tissues [41].

#### Detection of KS-specific viral transcripts and quantitation of viral load.

Utilization of single cell RNAseq, bulk RNAseq, and DNA-based quantitation assays on a single primary sample not only compensates for limitations of each methodology but also offers complementary insights and quality control for each sample. For example, *Kaposin (K12*) mRNA is detectable in scRNAseq data, but splice variants of *Kaposin* transcripts have been described in KS that may be difficult to detect by scRNAseq, or quantitate via ddPCR. We utilized probe-capture RNAseq for detection and quantitation of viral genes involved in lytic and latent viral gene expression in bulk RNA from a primary lesion and also for the detection of specific splice variants of Kaposin mRNA (S13 Fig). Similarly, in order to rapidly quantify KSHV viral load (the relative abundance of viral genomes per cell in a sample), primers and a probe were designed for a KSHV-specific digital droplet PCR assay (S13 Fig and S1 Methods). These tools were utilized to quantitate viral DNA and RNA in a primary KS sample from subject KS9 in addition to scRNAseq studies.

#### Evaluation of therapeutic interventions.

By evaluating samples obtained before and after therapeutic intervention, scRNAseq can be used to identify the effects of therapy on viral gene expression, as well as tumor cell abundance and gene expression. In addition, scRNAseq can assess the impact of therapy on the abundance and expression of cells in tumor stroma and peripheral blood. To evaluate these potential applications in our samples, we compared three pairs of serial samples, each before and after introduction of antiretroviral therapy (S14A Fig), nivolumab and ipilimumab therapy (S14B Fig), or pomalidomide therapy (S14C Fig). Interestingly, all three of these treatments resulted in the expansion of epidermal growth factor-like domain multiple 7 positive (*EGFL7+*), *FLT4+, PECAM1+* endothelial cells and the emergence of distinct populations of KSHV+ and KSHV- endothelial cells in the skin lesions after therapy (S14A-C Fig). Under unsupervised clustering, endothelial cells in post-therapy samples consistently formed clusters differentiated by the presence or absence of KSHV+ cells, with spatially separate migration on PCA dimensionality reduction (S14A-c, S14B-c and S14C-c Fig), and expression of several host cell markers. This endothelial cell clustering pattern was not observed pre-therapy. Genes associated with CD8+ T cells were also elevated after therapy in all three cases suggesting the possibility of crosstalk between activated CD8+ T cells, T cell-reactive stromal endothelial cells, and KSHV-infected cells in skin lesions (S14A-d, S14B-d and S14C-d Fig).

## Discussion

KS, like other KSHV-associated diseases, exhibits widely different transcription programs [16,42,43]. Several recent studies have applied scRNAseq to acute virus infections [44–48], as well as latent infection with human cytomegalovirus and other herpesviruses [49–51]. In a previous scRNAseq study, Landis *et al*. found latency-associated transcripts in the majority of cells of two KSHV-infected PEL cell lines [17]. Fewer than 1% of cells expressed lytic viral RNAs, with a predominance of early over late lytic viral transcripts. Jung *et al.* performed scRNAseq on KSHV infected 3-dimensional organoid cultures. They noted high levels of lytic replication, a unique pattern of lytic K2-K5 gene expression, and marked changes in host gene expression in infected and uninfected cells [52].

The current study provides the first single cell transcriptomic analysis of primary KS tumors and PBMC samples from different subtypes of KS, including cKS, iKS, and epKS. In this study, KSHV transcripts were found in a minority of cells in each KS tumor but not in the peripheral blood. PCR assays have detected KSHV DNA in peripheral blood mononuclear cells in 52% of epKS subjects [53]. The lack of KSHV+ cells in PBMCs in this study could be due to low levels of expression, a limitation of the sensitivity of our current scRNAseq technology or analysis, or both. Future studies using CITE-Seq with a custom panel of KSHV antibodies could improve the sensitivity of this analysis.

A novel finding from our study was the consistent presence of two separate clusters of KSHV-infected cells in skin tumors, differentiated by the presence or absence of *CD34* expression as well as ribosomal genes and housekeeping genes like *GAPDH*. The *CD34*- cluster was consistent with a lymphatic endothelial cell (LEC) lineage and was characterized by proliferative gene expression and voltage-gated ion channels [54]. The *CD34*+ cluster expresses many genes that correlate specifically with high endothelial venule cells which promote lymphocyte extravasation and maintain elevated levels of ribosomes and secretory vesicles [25]. However, this cluster is also enriched in genes associated with vascular endothelial “tip” cells, which drive angiogenic sprouting, are motile, and express long filopodia [27,28,55]. Cells in these clusters shared common biomarkers of endothelial cells, including *PECAM1, PROX1,* and *CD36*. However, in addition to marked differences in viral and cellular gene expression, we observed differential expression of several biomarkers, such as *CD34, LYVE1, PLVAP, and PDPN*. We conjecture that challenges in developing KS tissue culture models may be due to lack of cultivation conditions that include both the *CD34*- and *CD34*+ KSHV infected cell types. It is also possible that the characteristics of one or both of these subtypes of KSHV infected cells causes KS to be intrinsically sensitive to tumor immunity.

Interestingly, a study proposed a “transcriptional reprogramming” mechanism in which overexpressing *PROX1,* a known upregulated gene in KS, suppressed blood vascular endothelial cell (BVEC) genes and induced the LEC transcriptional program [56]. We found consistent upregulation of *PROX1* in both subtypes of KSHV-infected cells. This finding supports a potential lineage relationship between the two infected clusters observed in our study, and suggests a potential *PROX1* role in endothelial cell differentiation and/or malignant transformation in KSHV pathogenesis.

While latency-associated transcripts predominated, lytic transcripts were also identified in both KSHV-infected cell subtypes in our study. Although we cannot exclude some level of KSHV reactivation during sample processing, we consistently found lytic transcripts in all cases. The most abundantly expressed viral genes in our samples were the latency cluster genes downstream of the LTd promoter, a pathogenesis factor that encodes at least three proteins (v-CYC, v-FLIP and Kaposin); and K5, an early lytic ubiquitin ligase with immune evasion functions [57]. *CD34*- clusters showed higher average viral reads per cell than *CD34*+ clusters. One notable exception was *K5*, which was more highly expressed in *CD34*+ clusters while being predominant in both. A potential explanation for this over-representation could be related to genomic rearrangements, which were not fully investigated in this study but likely existed in one sample. One study showed *K5* overexpression in almost a third of KS lesions, due to similar *de novo* KSHV genomic rearrangements of a 1.5kb section containing the *K5* and *K6* genes [41].

Differential expression of host genes, such as *GAPDH* and *CD34*, in KSHV infected cell clusters did not correlate with the latent or lytic viral programs. Pardamean et al described two lytic-associated mechanisms of KSHV host gene shutoff, involving *v-SOX* and *ORF10* expression during early and late lytic replication, respectively [58]. We detected no *v-SOX* or *ORF10* transcription in any of our samples, and we found notably low expression of *GAPDH* and other host genes in infected clusters with low lytic transcripts. It is unclear whether host gene shutoff is induced by *v-SOX* and/or *ORF10* expression below the level of detection for our assay, or other host gene inhibition mechanisms are at play.

Our study identified several potential cellular biomarkers of KSHV infection, including some that were previously described [29–31]. One of these markers, *FLT4*, has previously been linked to malignancy [59] and discussed as a potential therapeutic target [60]. Another marker, *PROX1*, has been found to co-localize with *CD34* in KS lesions [29] and other cancers [61], and may be involved in regulation of endothelial to mesenchymal transition. An interesting finding was the expression pattern of *LYVE1*, a known lymphatic endothelial marker, which was present in both KSHV-infected clusters and particularly overexpressed in *CD34*+, transcriptionally active cells. *LYVE1* has been described in close correlation with *PROX1* in “budding endothelial cells”, a pattern thought to be a hallmark of lymphatic phenotype induction in budding vascular endothelium [24,54]. *LYVE1* and *PROX1* colocalization was also observed in tumors and tumor-adjacent lymphatic vessels [24]. Our findings support a role of endothelial phenotype transition in KSHV-induced host cell changes and oncogenesis.

The expression of VGSCs in both clusters of KSHV-infected cells was a novel observation. VGSCs are abnormally expressed in many types of cancers, and their level of expression and activity are related to the aggressiveness of the disease. Their tumor effects may be mediated through sodium ion effects, membrane potential modulation, or other pathways [62]. Of particular note was *SCN9A* (*Nav1.7*), a tetrodotoxin-sensitive VGSC with known roles in angiogenesis and regulation of chemotaxis [63]. *SCN9A* expression and activity were shown to steadily increase in herpes simples virus type 1 latent infection [64]. *SCN9A* is normally expressed in skin vasculature [65], and it has been associated with several cancer types, including endometrial, gastric, and prostate carcinomas [66–68]. In this study, *SCN9A* was tightly correlated with viral gene expression. Whether *SCN9A* could potentially be involved in KSHV-induced angiogenesis and blood vessel migration remains to be determined. Nonetheless, *SCN9A* emerges as a top RNA biomarker candidate of KSHV infection within primary KS lesions. Notably, another cation ion channel, despite relative low levels of expression, may be a therapeutic target in Kaposi sarcoma [69].

A recent bulk RNAseq study identified two types of KS lesions, inflammatory and proliferative, distinct by host gene transcription patterns [15]. Of the transcripts associated with the inflammatory subtype, we detected cell migration-inducing and hyaluronan-binding protein (*KIAA1199*; *CEMIP*) expression in all KS tumor samples, in both *CD34*+ and *CD34*- clusters. *CEMIP* was among the most commonly expressed genes among KSHV infected cells. Vesicle amine transport protein 1 homolog (*Trixis californica*)-like (*VATL1*) was also expressed in both infected clusters, to a lesser level and not in all samples; and *LY6H* was enriched in *CD34*+ clusters. Additionally, the proliferative subtype marker annexin A8-like 1 (*ANXA8L1*) was detected in non-infected epithelial cells and keratinocytes in all of our samples. While the inflammatory and proliferative distinction was not readily apparent in our analysis, quantification using single cell technology may further elucidate the transcriptional patterns of these newly identified markers.

Among the KSHV-negative cell population in KS tumors were T cells. KSHV-specific CD8 T cells are known to recognize and kill cells expressing KSHV lytic antigens [70], and they are more frequent in KSHV-seropositive individuals without KS than those with active disease, suggesting an anti-tumor role of these cells [71]. Perhaps reflective of these anti-tumor and anti-viral functions, the number of KSHV+ cells in our sample tumors were inversely proportional to immune cell number. One caveat to this relationship is that the proportion of immune cells in each sample also positively correlated with the total number of cells in the sample, though there was no correlation between percent of KSHV positive cells and total number of cells. In distinction to previous reports [72,73], we noted clonally expanded CD8+ T cells in tumors in paired tumor and blood samples, as well as longitudinal tumor samples. TCR Vβ CDR3 sequences of T cell clones in the peripheral blood were similar, but not identical, to a clone known to be directed against the major KSHV capsid protein [40]. Another KS PBMC single cell study [33] also reported common TCR sequence motifs of unknown antigen specificity, postulating a potential poly-specific T cell response to KSHV. Further investigation is warranted to elucidate whether T cell clonal expansion is indeed directed against KSHV, cellular tumor-specific antigens, HIV-1, or other persistent viral infections in these individuals.

To evaluate the potential feasibility of scRNAseq to monitor KS therapy, we examined samples obtained longitudinally from three individuals. In one individual each, we assessed the effects of antiretroviral therapy, immune checkpoint inhibition, and pomalidomide therapy. With each therapy we noted an increase in the abundance of stromal endothelial cells, distinct from both KSHV+ endothelial cell subtypes in unsupervised cluster formation, expression of KSHV genes, and KSHV infection markers in host cells. This change correlated with elevated expression of genes associated with tumor infiltrating CD8+ T cells. Studies of additional individuals are warranted to comprehensively assess effects of immunotherapy on the KS tumor microenvironment.

Our study has several limitations. The sensitivity of our scRNAseq assay may play a role in our consistently low KSHV detection in primary skin tumors compared to other assays [74,75], as well as the rarity of EBV, KSHV or HIV-1 detection in PBMC samples. Whether assay sensitivity prevented KSHV detection in non-endothelial tumor cell types, such as epithelial and immune cells, is unknown. Another limitation is that we did not examine micro- or long non-coding RNAs [76,77]. Our subjects constitute a small, heterogeneous subject pool of different age ranges and gender, who had different KS subtypes. While some demographic traits, such as gender, have not been found to significantly influence KS transcriptomics (34), the inter-individual differences in scRNAseq between and within tumors remain to be characterized. In addition, subjects with epKS had received differing durations of antiretroviral therapy. Nevertheless, the current study demonstrates the feasibility and utility of scRNAseq for interrogating the KS biology.

Taken together, these studies demonstrate the feasibility of single cell, multiomic analyses to characterize the malignant and stromal composition of primary KS blood and tumor tissue, quantitate viral and host gene expression, identify novel prognostic and predictive biomarkers and potential therapeutic targets, and evaluate the efficacy of therapeutic interventions.

## Materials and methods

### Ethics statement

The study compiled with ethical standards outlined in the Belmont Report and was approved by the Washington University Institutional Human Studies Review Committee. Formal written informed consent was obtained from each subject at the time of sample collection.

### Biopsies and skin cell dissociation

Viably frozen (in Bambanker, serum-free cell freezing media. Sigma) single-cell suspensions were prepared from fresh primary skin biopsy samples using enzymatic digestion and gentle manual tissue dissociation (Whole Skin Dissociation Kit, Miltenyi Biotech) and thawed immediately prior to submission for single cell RNA sequencing. It should be noted that sample preparation without enzymatic digestion dramatically diminished the diversity and abundance of cell populations obtained from skin lesions. An estimated 50,000 cells were submitted for sequencing for each sample, though there was some variation between samples in the viable cells submitted.

### scRNAseq

Libraries were prepared using the 10x Genomics 5′ or 3′ immune profiling kit-snRNA-seq protocol (GTAC@MGI). The resulting 10x library was sequenced on an Illumina S4 flow cell (300 cycles targeting 100,000 reads/cell). Alignment and gene expression quantification were performed with CellRanger multi pipeline (v7.1.0). The feature-barcode matrices were QCed, normalized and scaled using Seurat’s (v4.2.1) default settings. Principal component (PC) analysis was performed based on selected high variable genes and clustering of cells was performed using resolution = 0.7. Dimensionality reduction and visualization were performed using Seurat’s t-SNE and UMAP functions. Cells were annotated with SingleR (v2) using expression profiles from the Human Primary Cell Atlas (HPCA) dataset. Finally, differential expression analyses was performed using Seurat’s FindMarkers function with the Wilcoxon Rank Sum method (logfc.threshold=0.2, min.pct=0.05). Volcano plots were generated using EnhancedVolcano (v1.16.0) for visualization of the top differentially expressed genes.

Reads from single cells obtained for each sample were mapped against the human genome (GRCh38/hg38), the KSHV genome (Strain GK18, RefSeq Organism 37296, ViralProj14158, GCF_000838265.1; 137,969 bp genome) and the HIV-1 genome (RefSeq GCF_000864765.1; ViralProj15476; 9,181 bp genome), visualized using t-distributed stochastic neighbor embedding (t-SNE) or uniform manifold approximation and projection (UMAP) plots, and clustered to enable cell type identification. Analysis: Default parameters for t-SNE clustering used the top 10 principal components from the principle component analysis (PCA) step as initialization for secondary analysis. The reference dataset from the Human Primary Cell Atlas (HPCA) was used to annotate clusters using SingleR. Single-cell transcriptomic data is available at dbGaP study accession number phs003800.v1.p1.

### TCRseq analysis

For TCR analysis, the filtered contig annotation files from CellRanger were loaded into R (v4.4.0), and the scRepertoire package (v2.0.0) was used to perform analysis. To format and clean the data, the ‘combineTCR’ command was called with parameters ‘removeNA = T’ and ‘removeMulti = T’. The resulting list of matrices was used as input to downstream analysis and plotting commands. Clonal abundance barplots were generated using the ‘clonalHomeostasis’ command with parameter ‘cloneCall = “strict”’. Motif analysis was performed on the cleaned TCR data using the GLIPH2 software. For the reference data, the combined CD4+ and CD8+ reference was used, and the literature-based reference (version 1.0) was selected. The ‘all_aa_interchangeable’ parameter was set to YES. HLA typing data was not available and therefore was not provided [78].

### Immunohistochemistry and immunofluorescence microscopy

Formalin-fixed paraffin-embedded tissue slides were deparaffinized and rehydrated (60^o^C for 30 min followed by ethanol series and two washes in water for 10 minutes total). Antigen retrieval was performed by the Heat-Induced Epitope Retrieval (HIER) method in 0.1M citrate buffer (pH 6) under pressure for 20 minutes. Blocking included room temperature incubation in 1% goat normal serum, 1%BSA, 0.1% Tween 20 in Dulbecco’s phosphate-buffered saline (dPBS) for 1 hour followed by three, five-minute rinses in dPBS. Slides were incubated in rabbit SCN9A (1:100; LSBio, Lot # 198169) and rat LANA (1:500; EMD Milipore Corp, Lot # 4122574) primary antibodies overnight at 4^o^C, then washed three times in (dPBS). Slides were incubated in polyclonal goat anti-mouse and goat anti-rabbit secondary antibodies (1:100) for 1 hour at room temperature, washed three times in dPBS, and cover-slipped in mounting medium. Imaging was performed using a Keyence BZ-X810 high resolution microscope at 20X magnification. Each slide was scanned using multipoint acquisition with automated focusing using Hoechst stain fluorescence at 1/80s DAPI, 1/2s LANA, 1/2s SCN9A.

### Digital droplet PCR (ddPCR)

Viably frozen single-cell suspensions of a primary KS skin biopsy sample from subject KS9 were thawed from stocks. Then, digital droplet PCR was used to quantify copies of viral gene *K1* using the QX200 Droplet Digital PCR System (BioRad). ddPCR was performed as described previously [79]. Briefly, fifty ng of DNA were loaded per sample. Probe sequence was 5’-/56-FAM/CGG CCC TTG/ZEN/TGT AAA CCT GTC/3IABkFQ/ -3’, and the primer sequences were 5’- GTT CTG CCA GGC ATA GTC -3’ and 5’- GCC AGA CTG CAA ACA ACA TA -3’. Results were detected with QX200 Droplet Reader (BioRad).

## Supporting information

S1 Fig Primary Samples: 10X Cell Ranger, graph-based, UMAP cluster plots of the 25 primary patient samples including A) 20 skin biopsies and (B) 5 PBMC preparations included in this study as described in Table 1. Sample names are color coded by batch: Red (batch 1), Blue (batch 2), Black (batch 3). Cluster colors are generated independently for each sample image and do not necessarily correlate to the same cell types from image to image. C) The pie chart reflects the percent of total KSHV+ cells contributed by each sample to the composite data sets in Figs 1-4. The bar graph shows the percent of cells in each sample that express a single read of a single KSHV gene (blue) and >1 read of one or more KSHV transcripts (orange). Dotted line represents the 2% cut-off (which excluded cells with a single read of a single KSHV gene) used in Fig 5 to define KS6B, KS8, and KS9 as the only samples in which the blue portion of each bar exceeds 2%. Sample KS11 was excluded from the analysis due to poor quality (low fraction of confidently mapped reads in cells). D) Heat Map of Differential Gene Expression between KSHV+ and KSHV- cells. 50 of 2,398 differentially expressed genes with p value < 0.01 are shown. All genes shown have p values = 0).
(PDF)

S2 Fig Publically available scRNaseq data of skin. A) UMAP of 9424 skin cells from CELL×GENE | Explorer (cziscience.com) Tabula Sapiens (skin from a healthy male, 10X Genomics 3ʹ v3 kit). B) Log2 *CD34*, SCN9A, and GAPDH expression in endothelial cells. C) Scatter plot of Log2 *CD34* vs Log2 GAPDH with cells color coded based on *CD34* expression. D) Violin plots of Log2 expression of GAPDH in *CD34*^HI^KSHV+, *CD34*^LO^KSHV+, KSHV- Endothelial Cells compared to Log2 expression of GAPDH in Tabula Sapiens database. Reference: The Tabula Sapiens Consortium* The Tabula Sapiens: A multiple-organ, single-cell transcriptomic atlas of humans.*Science*376,eabl4896(2022). https://doi.org/10.1126/science.abl4896. p values calculated in Cell Ranger are adjusted using the Benjamini-Hochberg correction for multiple tests ** = p <1e-5; *** = p <1e-15
(PDF)

S3 Fig Potential False Positives. 10X Cell Ranger, graph-based, t-SNE cluster plots of KS6B and KS8 (2 of 3 samples with >2% KSHV positive cells). A) Number of KSHV+ cells (purple) in each panel is based on min read threshold cut-off indicated (>0 through >5). B) Cells with a single read of a single KSHV gene are shown in the plots and the identity of the viral gene and number of cells expressing a single read of that gene are shown in the tables. The graph indicates the percentage of suspected false positive cells at each min read cut-off threshold. Suspected false positive cells are any cell positive for KSHV but not included in the clusters containing >99% of infected cells at the highest min read cut-off threshold (>5). KSHV LAT transcript reads integrate those from K12, LANA, and v-FLIP.
(PDF)

S4 Fig Presence of distinct clusters of KSHV+ cells in primary KS samples. A-O) UMAP plots of unsupervised clusters of cells from 15 skin samples (samples with >50 KSHV+ cells). KSHV+ cells expressing two different subsets of genes present in distinct endothelial subclusters are distinguishable in each sample based on differential gene expression; Red = *CD34*^+^GAPDH^HI^EP300^LO^ cells; Black = *CD34*^-^GAPDH^LO^EP300^HI^ cells as depicted in violin plots. KS11 was excluded due to poor sample quality. p values calculated in Cell Ranger are adjusted using the Benjamini-Hochberg correction for multiple tests.
(PDF)

S5 Fig Characteristics of *CD34*^HI^ and *CD34*^LO^ KSHV+ cells. A) UMAP projections of KSHV+ Cluster 13 cells as defined in Fig 3 color coded based on Log2 expression of indicated endothelial markers. B) Left: Violin plots of Log2 total UMI count of all genes per cell for each graph-based t-SNE cluster in three KS samples (KS6B, KS8, KS9, all 3 samples with >2% KSHV positive cells) generated in Loupe Browser 8. Two subtypes of KSHV-infected cells are highlighted in each sample (with red circles and black squares) corresponding to graph-based unsupervised clusters containing *CD34*^HI^ KSHV+ cells and *CD34*^LO^ KSHV+ cells, respectively. Right: Violin plots of Log2 total UMI count per cell as well as Log 2 sum of lytic and latent gene expression in cells within each KSHV-infected cluster in KS8. p values calculated in Cell Ranger are adjusted using the Benjamini-Hochberg correction for multiple tests. C) t-SNE cluster plot of KS6B (1 of 3 samples with >2% KSHV positive cells) with *CD34*^HI^ and *CD34*^LO^ KSHV+ cell clusters highlighted. Differential expression of GAPDH in *CD34*^HI^ and *CD34*^LO^ KSHV+ cells graphed based on percent of GAPDH positive cells vs number of GAPDH reads per cell with table indicating the average number of GAPDH reads per cell in each group, the Log2 fold change, and the p value. D) a) t-SNE plots of KS6B, KS8, and KS9 skin samples (all 3 samples with >2% KSHV positive cells) with Log2 sum gene expression of latent KSHV genes (top) and lytic KSHV genes (bottom) in each cluster. b) Corresponding volcano plot depicts ratio of Log2 fold change between *CD34*^HI^ and *CD34*^LO^ KSHV+ cells vs. p-value (using paired t-test) to compare average reads per cell for selected human and KSHV genes for KS6B, KS8, KS9. c) Volcano plot comparing *CD34*^HI^ and *CD34*^LO^ KSHV+ cells from all samples indicating that the only viral genes significantly differentially expressed between the two clusters are K5 (in *CD34*^HI^ cells) and K12 (in *CD34*^LO^ cells). E) Genes expressed in high endothelial venules (HEVs) in mice are also significantly enriched in *CD34*^HI^ KSHV+ *CD34* Cluster 13 cells (p<0.01) were compared to genes identified in a study by 2019 Veerman et al. (PMID: 30865898) describing scRNAseq analysis of high endothelial venules (HEVs) in mice. Table a lists 20 genes (including Log2 expression and p-value in *CD34*^HI^ cells) that are also known to be expressed in HEVs (as described in Veerman et al.). The volcano plot in panel b indicates the log2 fold change and p value of 812 genes that are significantly different between *CD34*^HI^ KSHV+ cells and *CD34*^LO^ KSHV+ cells that are also significantly different between HEVs and blood endothelial cells (bECs) in Veerman et al. The volcano plot (depicting log2 fold change and p-value) in panel c shows that the majority of the genes differentially expressed in mouse HEVs in Veerman et al. are also significantly expressed in *CD34*^HI^ KSHV+ cells compared to *CD34*^LO^ KSHV+ cells (genes in the volcano plot are listed in the table along with p value and log2 fold change and color coded as shown it the legend of the volcano plot). F) t-SNE plots of selected KS samples (all 3 samples with >2% KSHV positive cells) indicating *CD34*^HI^ KSHV+ cells (red circles) and *CD34*^LO^ KSHV+ cells (black boxes) and highlighting genes (Log2 expression) identified by Moorad et al. as associated with inflammatory vs. proliferative KS lesions. G) Heat map of differential gene expression between *CD34*^HI^ and *CD34*^LO^ KSHV+ cells. 100 of 3,546 differentially expressed genes with p value < 0.01 are shown. All genes shown have p values <1E-200).
(PDF)

S6 Fig The KSHV Latency Cluster. A graphical depiction of the KSHV latency cluster with a corresponding bam file viewed in IGV of a representative KS skin sample. Pink and blue bars represent reads mapped to the KSHV genome. Long blue horizontal lines represent reads that span splice junctions and correspond with blue arcs which quantitate splice junctions. The grey bar graph indicates read depth and the genome coordinates correspond to the NCBI KSHV reference genome GCF_000838265.1. In this sample, the LTd promoter is most active promoter in the Latency cluster as previously described in Rose et al (PMID: 30557332).
(PDF)

S7 Fig Expression of voltage-gated sodium channel genes in KS. A) t-SNE plots of selected KS samples (KS8, 1 of 3 samples with >2% KSHV positive cells) showing unsupervised clustering and log2 expression of KSHV latent (Latency cluster) and lytic genes (K5) and highlighting the expression (purple = reads >1) of 14 voltage-gated sodium channel genes and the correlation between SCN9A and KSHV. B) t-SNE plots of selected KS samples (KS9, 1 of 3 samples with >2% KSHV positive cells) showing unsupervised clustering and log2 expression of KSHV latent (Latency cluster) and lytic genes (K5) and highlighting the expression (purple = reads >1) of 14 voltage-gated sodium channel genes and the correlation between SCN9A and KSHV. C) a) Violin plots of SCN9A expression and KSHV gene expression in each cluster of the graph-based t-SNE plot of KS6B. b) t-SNE plots of three KS samples (KS6B, KS8, KS9, all 3 samples with >2% KSHV positive cells), comparing the abundance, location, and expression of KSHV+ cells to SCN9A expressing cells (log2 scale). p values calculated in Cell Ranger are adjusted using the Benjamini-Hochberg correction for multiple tests. KSHV gene used for p value calculation is gp81.
(PDF)

S8 Fig SCN9A expression in a case of Kaposi sarcoma. Parts A-D show one section of a Kaposi sarcoma tissue block with SCN9A primary antibody used. Parts E-H show another section from the same Kaposi sarcoma tissue block, with isotype control antibody used instead of SCN9A primary antibody. Both sections show staining with Hoechst (for chromatin, A and E) and indirect immunofluorescence staining for LANA (B and F). LANA staining in both sections demonstrates expected variable nuclear staining with characteristic dot-like pattern in Kaposi sarcoma cells. SCN9A staining in the first tissue section (C) shows increased signal intensity compared to isotype control (G). D shows color overlay of LANA (red) and SCN9A (green), with increased green signal demonstrating SCN9A positivity as compared to the color overlay of LANA (red) and isotype control (absent) in H.
(PDF)

S9 Fig Markers used to Identify Monocytes and Macrophages in scRNAseq Data. A) t-SNE plot of unsupervised clusters (K-means 7 clusters) for KS7 (1 of 3 samples with >2% KSHV positive cells) listing 9 genes used to identify Macrophages (CD14, CD80, CD86, MARCO, TLR2, IL10, IL1B, IL1A, ITGAX) being enriched (Log2 fold change and p-value indicated) in cluster 1 (black dots) and being similar to all cells that meet the criteria of Log2 Sum expression (>2) for all of the nine genes or Reads per cell (>3) for any of the nine genes. B) a) UMAPs of KS6B PBMC and KS10 PBMC (2 of 4 PBMC samples) with corresponding violin plots showing log2 expression of CD4, CD8A, CD3E (marker for T cells), and CD14 (marker for Macrophages). CD4, unlike CD8A is more highly expressed in CD14+ cells than CD3+. In panel b, CD4 expression in 4 of 4 evaluable PBMC samples is higher in monocytes than CD4+T cells when plotted as reads vs number of positive cells in each sample.
(PDF)

S10 Fig Viral reads in PBMC samples detected in BAM files. 10X scRNAseq data for each sample was mapped against KSHV, HIV-1 and EBV genomes to generate BAM files. Reads mapping to unique barcodes were projected against composite UMAP objects of unsupervised clusters created in Seurat based on cells called by Cellranger as described in Fig 1. KSHV: 300K total KSHV reads map to 34,693 unfiltered barcodes. Filters applied for doublets, outliers, low quality cells, dying cells and a minimum of 2 KHSV transcripts dramatically reduce the number of cells defined as truly KSHV+ (red dots), also excluding 4 KSHV+ cells in PBMC samples (in KS10 PBMC and KS6A PBMC). HIV-1: 56 total reads mapped to 25 HIV-1+ barcodes in two samples (KS6A PBMC and KS10 PBMC) resulting in 23 unfiltered HIV-1+ cells 15 HIV+ cells after filtering (red dots). Reads in these 15 cells mapped almost exclusively to the viral LTRs. EBV: 222 total reads mapped to 100 EBV+ barcodes, resulting in 90 unfiltered EBV+ cells and 40 filtered EBV+ cells (red dots) all from the PBMC samples of KS6.
(PDF)

S11 Fig Expansion of CD8 T cell clones in primary KS. Three t-SNE plots from three samples from patient KS10, a blood sample and two skin samples, one before and one after therapy. Seven CD8+T cell clones found in each of the three samples are indicated in seven different colors in the t-SNE plots that correspond to the color bar shown adjacent to the table. The number of cells representing each CD8+T cell clone is indicated by “count” and the relative abundance of each clone is indicated by “rank” with the most abundant CD8+T cell lone being #1. The most abundant CD8+T cell clone in the KS tumor (Blue dots) is the same in both skin samples and is also present in the peripheral blood but is the 10^th^ most abundant T cell clone in the peripheral blood. Similarly the most abundant CD8+T cell clone in the peripheral blood (Red dots) is less frequently detected in the KS sample. These samples are from the only patient from who PBMC and 2 skin samples were obtained.
(PDF)

S12 Fig Amplification of the K5-K7 region in KS6B. A) Reads for viral genes flanking and including the K5-K7 region of the KSHV genome were obtained from KSHV+ cells from 4 KS skin tumors with >100 positive cells for both LANA and K12 (KS6B, KS8, KS9, KS10B, all 3 samples with >2% KSHV positive cells and one additional sample), normalized against the Latency cluster, were graphed as a ratio of the values obtained from sample KS9. Organization of the KSHV genome was reproduced from Prazsak et al (PMC10542539). A composite UMAP plot of all KSHV+ cells reveals that the KS6B sample is distinct from the other samples. B) Bam files of reads mapped to the KSHV genome for 4 KS skin samples and visualized in IGV reveal that KS6B (1 of 3 samples with >2% KSHV positive cells) has an amplification of the K5-K7 region that is not seen in the other three samples. Amplification of this region has been described in 9 of 32 KS samples from a Ugandan cohort by Santiago et al. (PMID:36441790)
(PDF)

S13 Fig Quantitation of KSHV RNA and DNA using RNAseq and ddPCR. A) Probe-capture, bulk RNAseq for KSHV latency genes ORF71 (v-FLIP), ORF72 (v-CYC), ORF73 (LANA), and ORFK12 (Kaposin) visualized on IGV. B) Digital Droplet PCR comparing DNA obtained from KS9 to three controls, i) water, ii) DNA from Jurkat cells, and iii) DNA from KSHV-expressing Vero cells showing number of positive droplets.
(PDF)

S14 Fig Therapeutic Responses in Primary KS. A) Primary KS tumor changes in one patient after ART initiation. KS tumor biopsy samples were obtained from Patient KS6 before and after initiation of antiretroviral therapy. KS6A represents the pre-therapy sample, and KS6B represents the post-therapy sample. a) Quantification of significant gene expression changes in KSHV+ tumor cells. Significant expression was determined by a p-value of <0.05 of the normalized mean gene counts in KSHV+ cells relative to KSHV- cells in the tumor. All red areas represent gene upregulation, and the blue area represents gene downregulation in KS6B. The yellow area represents genes that were upregulated in KS6A and downregulated in KS6B. b) Volcano plots of gene expression profiles of KSHV+ tumor cells in KS6A and KS6B. Due to high gene counts in KS6B, genes with a fold change between -3 and +3 were omitted. c) Cell composition of KS6A and KS6B tumors. Macrophage, CD8 T cells, and KSHV+ cells are shown on the left-hand t-SNE plots. Endothelial cells are shown on the right-hand t-SNE plots. The KSHV+ endothelial cell populations are boxed in red and the KSHV- endothelial cell population is boxed in blue. d) Volcano plot of T cell gene expression changes before and after therapy. The X axis represents the ratio of differential gene expression in T cells relative to all other tumor cells. The Y axis represents the p-value of KS6B fold change in logarithmic scale. B) Primary KS tumor changes in one patient after Nivolumab plus Ipilimumab treatment. KS tumor biopsy samples were obtained from Patient KS1 before and after a course of Nivolumab plus Ipilimumab therapy. KS1A represents the pre-therapy sample, and KS1B represents the post-therapy sample. a) Quantification of significant gene expression changes in KSHV+ tumor cells. Significant expression was determined by a p-value of <0.05 of the normalized mean gene counts in KSHV+ cells relative to KSHV- cells in the tumor. All genes represented in the diagram are upregulated in KSHV+ tumor cells. b) Volcano plots of gene expression profiles of KSHV+ tumor cells in KS1A and KS1B. c) Cell composition of KS1A and KS1B tumors. Macrophage, CD8 T cells, and KSHV+ cells are shown on the left-hand t-SNE plots. Endothelial cells are shown on the right-hand t-SNE plots. The KSHV+ endothelial cell populations are boxed in red and the KSHV- endothelial cell population is boxed in blue. d) Volcano plot of the T cell gene expression changes before and after therapy. The X axis represents the ratio of differential gene expression in T cells relative to all other tumor cells. The Y axis represents the p-value of KS1B fold change in logarithmic scale. C) Primary KS tumor changes in one patient after Pomalidomide treatment. KS tumor biopsy samples were obtained from Patient KS10 before and after initiation of pomalidomide therapy. KS10A represents the pre-therapy sample, and KS10B represents the post-therapy sample. a) Quantification of significant gene expression changes in KSHV+ tumor cells. Significant expression was determined by a p-value of <0.05 of the normalized mean gene counts in KSHV+ cells relative to KSHV- cells in the tumor. All red areas represent gene upregulation, and the blue area represents 2 downregulated genes in KS10B. b) Volcano plots of gene expression profiles of KSHV+ tumor cells in KS10A and KS10B. c) Cell composition of KS10A and KS10B tumors. Macrophage, CD8 T cells, and KSHV+ cells are shown on the left-hand t-SNE plots. Endothelial cells are shown on the right-hand t-SNE plots. The KSHV+ endothelial cell populations are boxed in red and the KSHV- endothelial cell population is boxed in blue. d) Volcano plot of the T cell gene expression changes before and after therapy. The X axis represents the ratio of differential gene expression in T cells relative to all other tumor cells. The Y axis represents the p-value of KS10B fold change in logarithmic scale. Larsson J (2021). *eulerr: Area-Proportional Euler and Venn Diagrams with Ellipses*. R package version 6.1.1, https://CRAN.R-project.org/package=eulerr.
(PDF)

S1 Table Most Common T Cell Receptor CDR3 Motif-Based Clonal Groups Found in KS PBMC Samples. Most common shared motifs in T cell receptor data from 4 PBMC samples. T cell clones were clustered based on sequence motifs likely to have a common antigen specificity using GLIPH2 software, returning 289 motif patterns (Fig 7B). The 10 most commonly shared clonal groups, listed here, are defined as those that included clones from at least 3 of the 4 PBMC samples. These data reflect the frequent use of the TRBJ1-1 segment in KS that we reported on Fig 7C.
(PDF)

S1 Methods Sequences for probe capture bulk RNAseq. (PDF)

## Financial statement

The funders had no role in study design, data collection and analysis, decision to publish, or preparation of the manuscript.
