# Supplementary material for: Single-Cell Transcriptomic Analysis of Kaposi Sarcoma
Source: PLoS Pathog. 2025 Apr 1;21(4):e1012233. doi: 10.1371/journal.ppat.1012233 (PMC11984749; doi:10.1371/journal.ppat.1012233)
Supplement: S1 Fig — Sample names are color coded by batch: Red (batch 1), Blue (batch 2), Black (batch 3). Cluster colors are generated independently for each sample image and do not necessarily correlate to the same cell types from image to image. C) The pie chart reflects the percent of total KSHV+ cells contributed by each sample to the composite data sets in Figs 1-4. The bar graph shows the percent of cells in each sample that express a single read of a single KSHV gene (blue) and >1 read of one or more KSHV transcripts (orange). Dotted line represents the 2% cut-off (which excluded cells with a single read of a single KSHV gene) used in Fig 5 to define KS6B, KS8, and KS9 as the only samples in which the blue portion of each bar exceeds 2%. Sample KS11 was excluded from the analysis due to poor quality (low fraction of confidently mapped reads in cells). D) Heat Map of Differential Gene Expression between KSHV+ and KSHV- cells. 50 of 2,398 differentially expressed genes with p value < 0.01 are shown. All genes shown have p values = 0). (PDF) [file ppat.1012233.s001.pdf]

FIGURE S1A

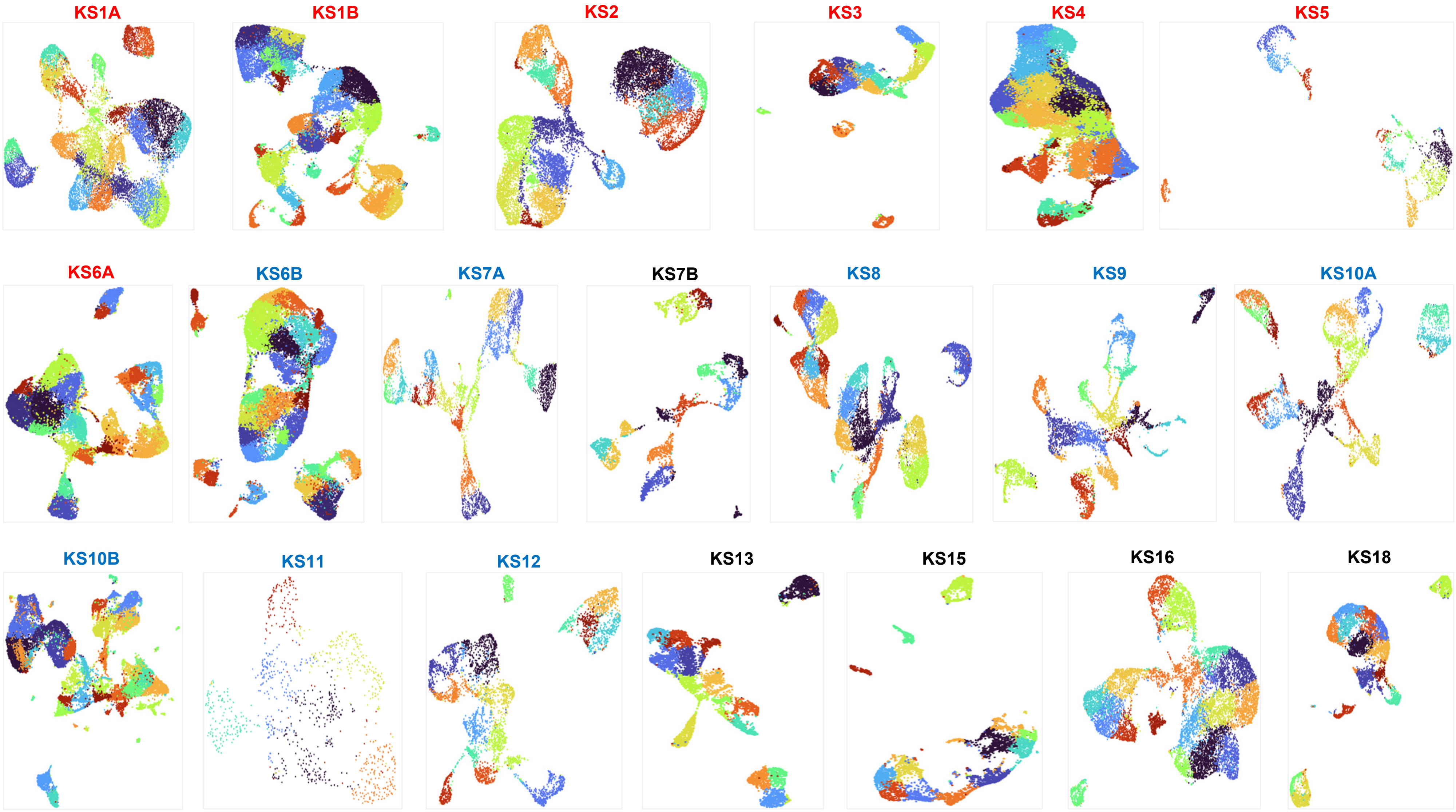
KS1A
KS1B
KS2
KS3
KS4
KS5
KS6A
KS6B
KS7A
KS7B
KS8
KS9
KS10A
KS10B
KS11
KS12
KS13
KS15
KS16
KS18

FIGURE S1B

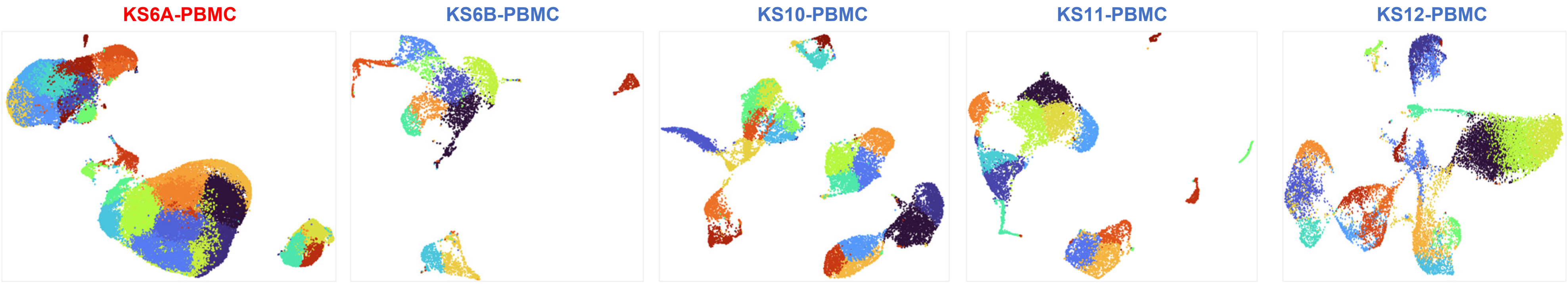

Figure S1A-B: Primary Samples: 10X Cell Ranger, graph-based, UMAP cluster plots of the 25 primary patient samples including A) 20 skin biopsies and (B) 5 PBMC preparations included in this study as described in Table 1. Sample names are color coded by batch: Red (batch 1), Blue (batch 2), Black (batch 3). Cluster colors are generated independently for each sample image and do not necessarily correlate to the same cell types from image to image.

FIGURE S1C

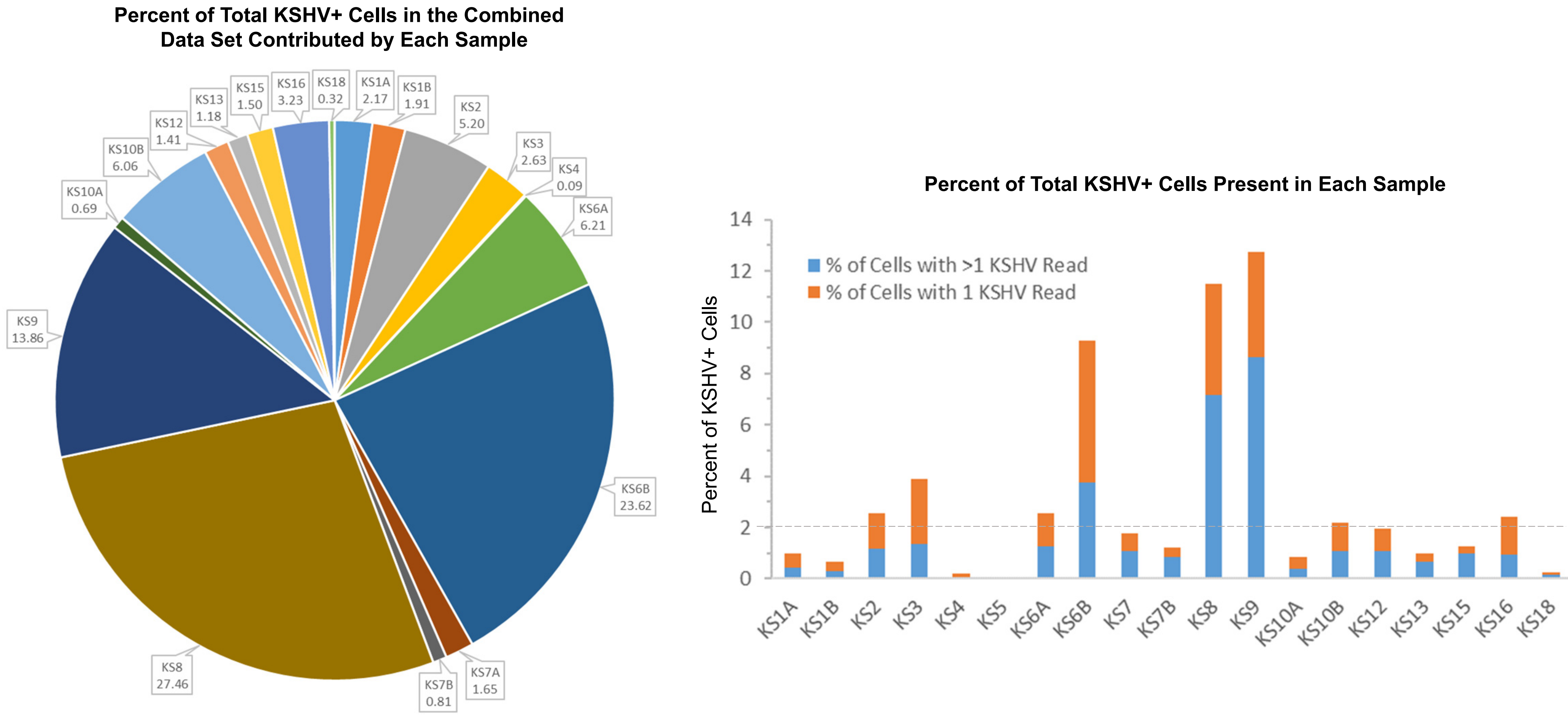

Figure S1C: Primary Samples: The pie chart reflects the percent of total KSHV+ cells contributed by each sample to the composite data sets in Figures 1-4. The bar graph shows the percent of cells in each sample that express a single read of a single KSHV gene (blue) and >1 read of one or more KSHV transcripts (orange). Dotted line represents the 2% cut-off (which excluded cells with a single read of a single KSHV gene) used in Figure 5 to define KS6B, KS8, and KS9 as the only samples in which the blue portion of each bar exceeds 2%. Sample KS11 was excluded from the analysis due to poor quality (low fraction of confidently mapped reads in cells).

FIGURE S1D

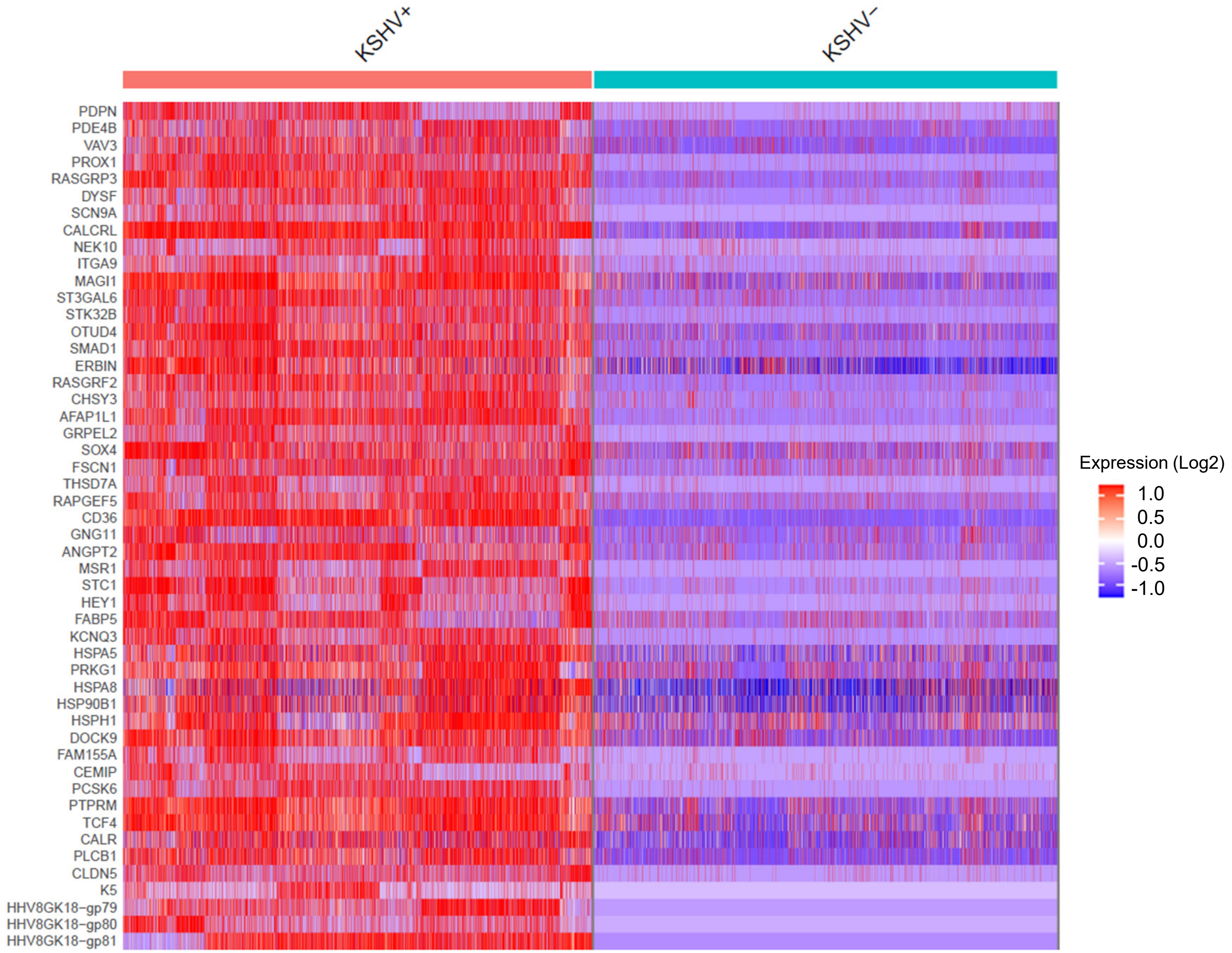

Figure S1D: Heat Map of Differential Gene Expression between KSHV+ and KSHV- cells. 50 of 2,398 differentially expressed genes with p value < 0.01 are shown. All genes shown have p values = 0).
