# Supplementary material for: Single-Cell Transcriptomic Analysis of Kaposi Sarcoma
Source: PLoS Pathog. 2025 Apr 1;21(4):e1012233. doi: 10.1371/journal.ppat.1012233 (PMC11984749; doi:10.1371/journal.ppat.1012233)
Supplement: S2 Fig — A) UMAP of 9424 skin cells from CELL×GENE | Explorer (cziscience.com) Tabula Sapiens (skin from a healthy male, 10X Genomics 3ʹ v3 kit). B) Log2 CD34, SCN9A, and GAPDH expression in endothelial cells. C) Scatter plot of Log2 CD34 vs Log2 GAPDH with cells color coded based on CD34 expression. D) Violin plots of Log2 expression of GAPDH in CD34HIKSHV+, CD34LOKSHV+, KSHV- Endothelial Cells compared to Log2 expression of GAPDH in Tabula Sapiens database. Reference: The Tabula Sapiens Consortium* The Tabula Sapiens: A multiple-organ, single-cell transcriptomic atlas of humans.Science376,eabl4896(2022). https://doi.org/10.1126/science.abl4896. p values calculated in Cell Ranger are adjusted using the Benjamini-Hochberg correction for multiple tests ** = p <1e-5; *** = p <1e-15 (PDF) [file ppat.1012233.s002.pdf]

FIGURE S2

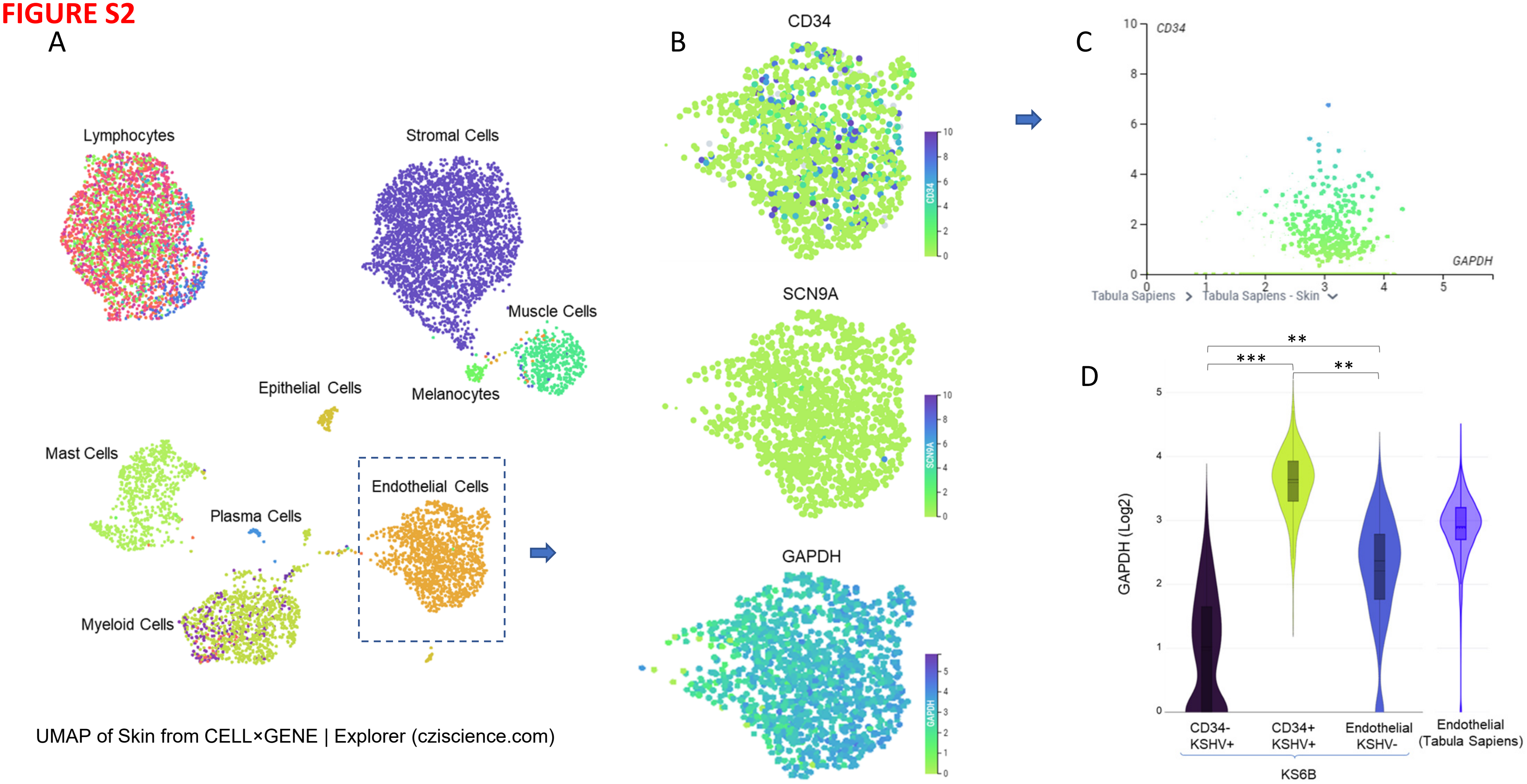

Figure S2: Publically available scRNaseq data of skin. A) UMAP of 9424 skin cells from CELL×GENE | Explorer (cziscience.com) Tabula Sapiens (skin from a healthy male, 10X Genomics 3' v3 kit). B) Log2 CD34, SCN9A, and GAPDH expression in endothelial cells. C) Scatter plot of Log2 CD34 vs Log2 GAPDH with cells color coded based on CD34 expression. D) Violin plots of Log2 expression of GAPDH in $CD34^{HI}$KSHV+, $CD34^{LO}$KSHV+, KSHV- Endothelial Cells compared to Log2 expression of GAPDH in Tabula Sapiens database. Reference: The Tabula Sapiens Consortium* The Tabula Sapiens: A multiple-organ, single-cell transcriptomic atlas of humans.*Science*376,eabl4896(2022).DOI:10.1126/science.abl4896. p values calculated in Cell Ranger are adjusted using the Benjamini-Hochberg correction for multiple tests ** = $p < 1e\text{-}5$; *** = $p < 1e\text{-}15$
