# Supplementary material for: Single-Cell Transcriptomic Analysis of Kaposi Sarcoma
Source: PLoS Pathog. 2025 Apr 1;21(4):e1012233. doi: 10.1371/journal.ppat.1012233 (PMC11984749; doi:10.1371/journal.ppat.1012233)
Supplement: S3 Fig — 10X Cell Ranger, graph-based, t-SNE cluster plots of KS6B and KS8 (2 of 3 samples with >2% KSHV positive cells). A) Number of KSHV+ cells (purple) in each panel is based on min read threshold cut-off indicated (>0 through >5). B) Cells with a single read of a single KSHV gene are shown in the plots and the identity of the viral gene and number of cells expressing a single read of that gene are shown in the tables. The graph indicates the percentage of suspected false positive cells at each min read cut-off threshold. Suspected false positive cells are any cell positive for KSHV but not included in the clusters containing >99% of infected cells at the highest min read cut-off threshold (>5). KSHV LAT transcript reads integrate those from K12, LANA, and v-FLIP. (PDF) [file ppat.1012233.s003.pdf]

FIGURE S3

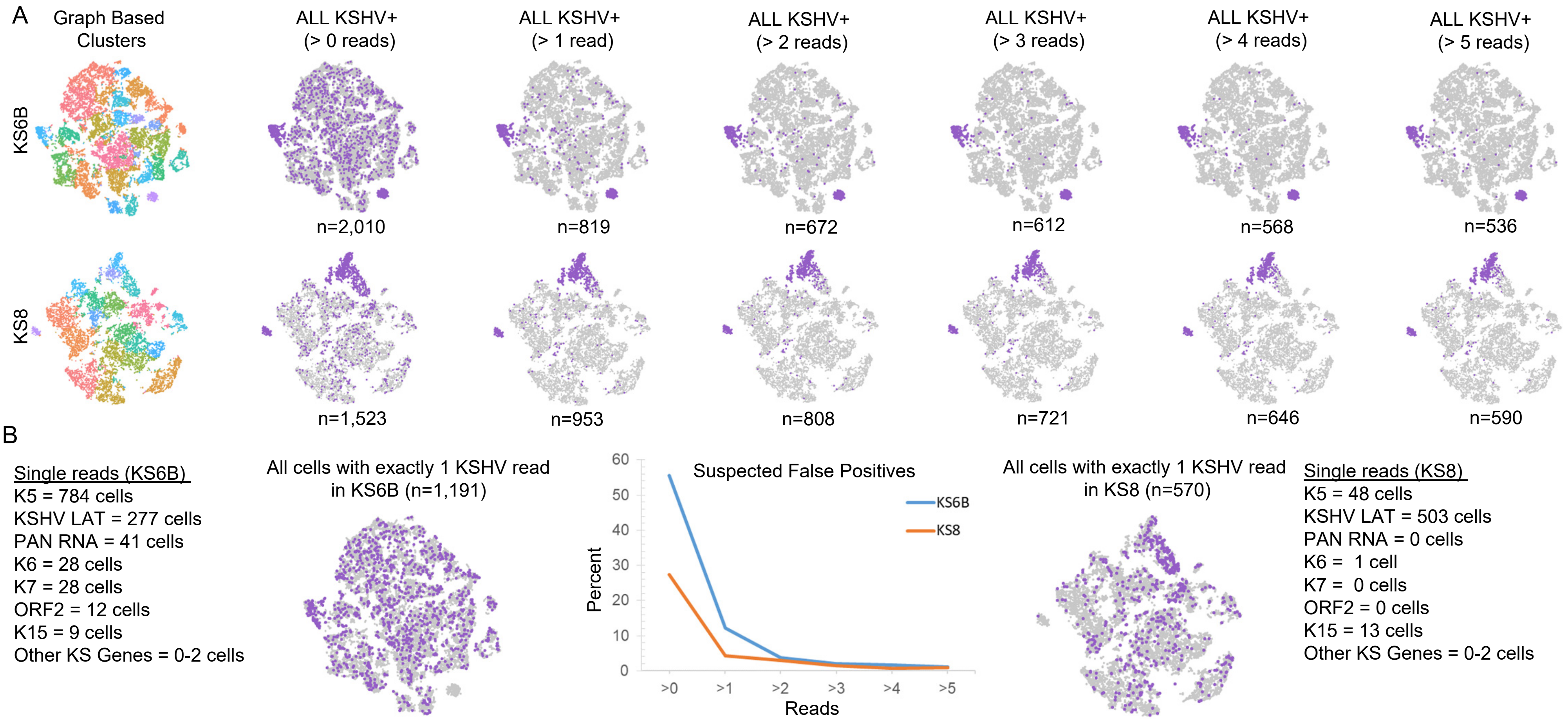

Figure S3: Potential False Positives. 10X Cell Ranger, graph-based, t-SNE cluster plots of KS6B and KS8. A) Number of KSHV+ cells (purple) in each panel is based on min read threshold cut-off indicated (>0 through >5). B) Cells with a single read of a single KSHV gene are shown in the plots and the identity of the viral gene and number of cells expressing a single read of that gene are shown it the tables. The graph indicates the percentage of suspected false positive cells at each min read cut-off threshold. Suspected false positive cells are any cell positive for KSHV but not included in the clusters containing >99% of infected cells at the highest min read cut-off threshold (>5). KSHV LAT transcript reads integrate those from K12, LANA, and v-FLIP.
