# Supplementary material for: Single-Cell Transcriptomic Analysis of Kaposi Sarcoma
Source: PLoS Pathog. 2025 Apr 1;21(4):e1012233. doi: 10.1371/journal.ppat.1012233 (PMC11984749; doi:10.1371/journal.ppat.1012233)
Supplement: S4 Fig — A-O) UMAP plots of unsupervised clusters of cells from 15 skin samples (samples with >50 KSHV+ cells). KSHV+ cells expressing two different subsets of genes present in distinct endothelial subclusters are distinguishable in each sample based on differential gene expression; Red = CD34+GAPDHHIEP300LO cells; Black = CD34-GAPDHLOEP300HI cells as depicted in violin plots. KS11 was excluded due to poor sample quality. p values calculated in Cell Ranger are adjusted using the Benjamini-Hochberg correction for multiple tests. (PDF) [file ppat.1012233.s004.pdf]

FIGURE S4A

KS1A

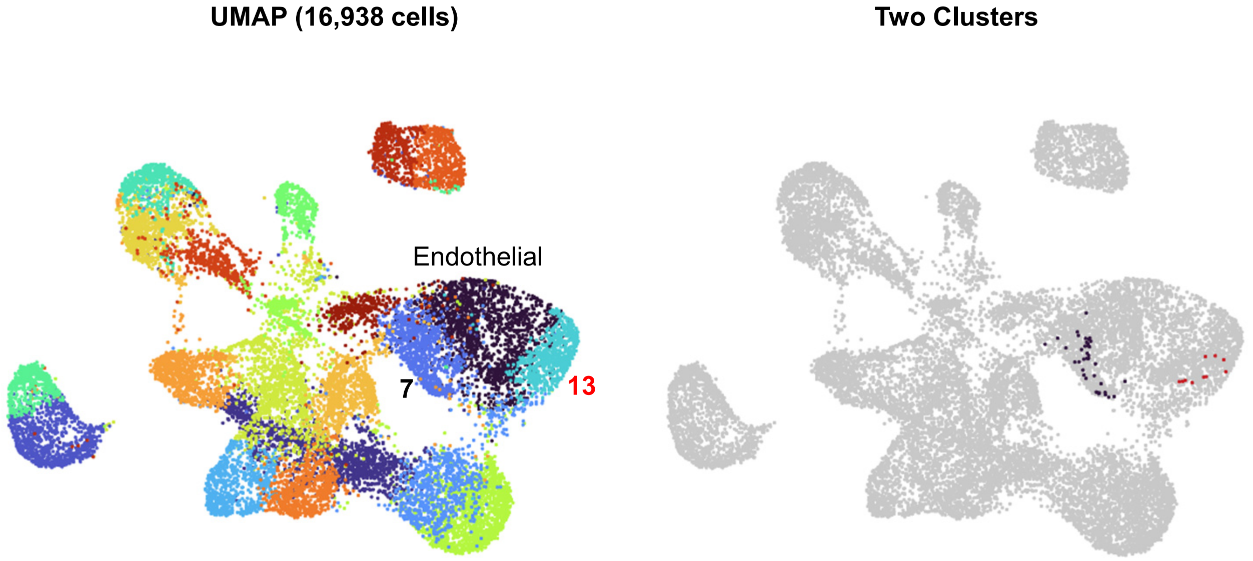

KSHV+ Cells >0 reads = 170; >1 read = 75

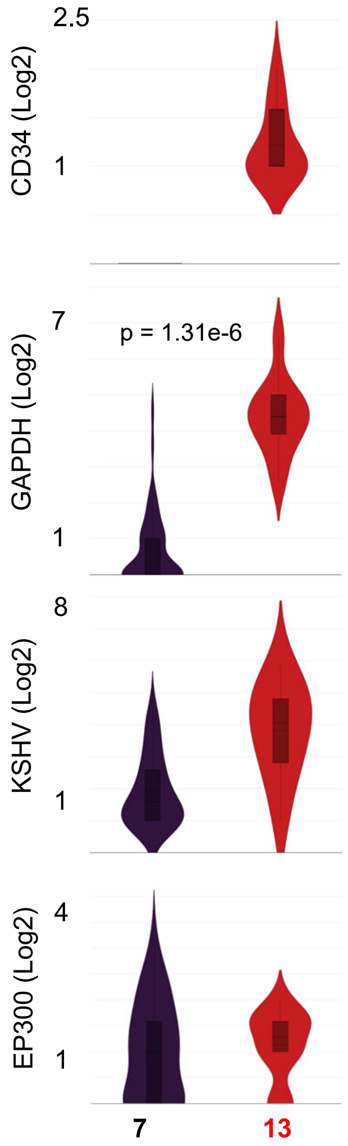

FIGURE S4B

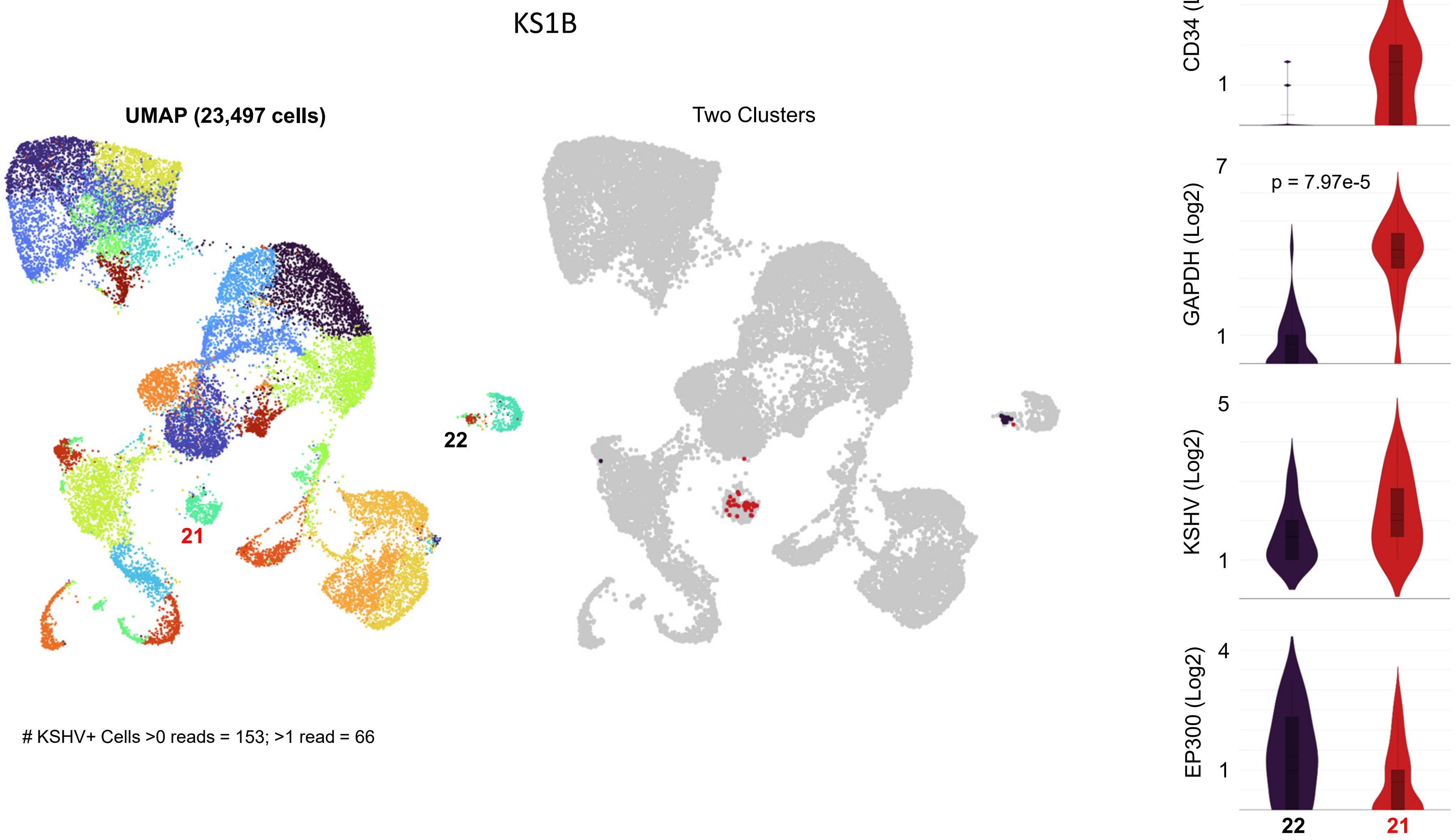
KS1B
UMAP (23,497 cells)
Two Clusters
22
21
KSHV+ Cells >0 reads = 153; >1 read = 66
CD34 (Log2)
GAPDH (Log2)
p = 7.97e-5
KSHV (Log2)
EP300 (Log2)
5
1
7
1
5
1
4
1
22
21

FIGURE S4C

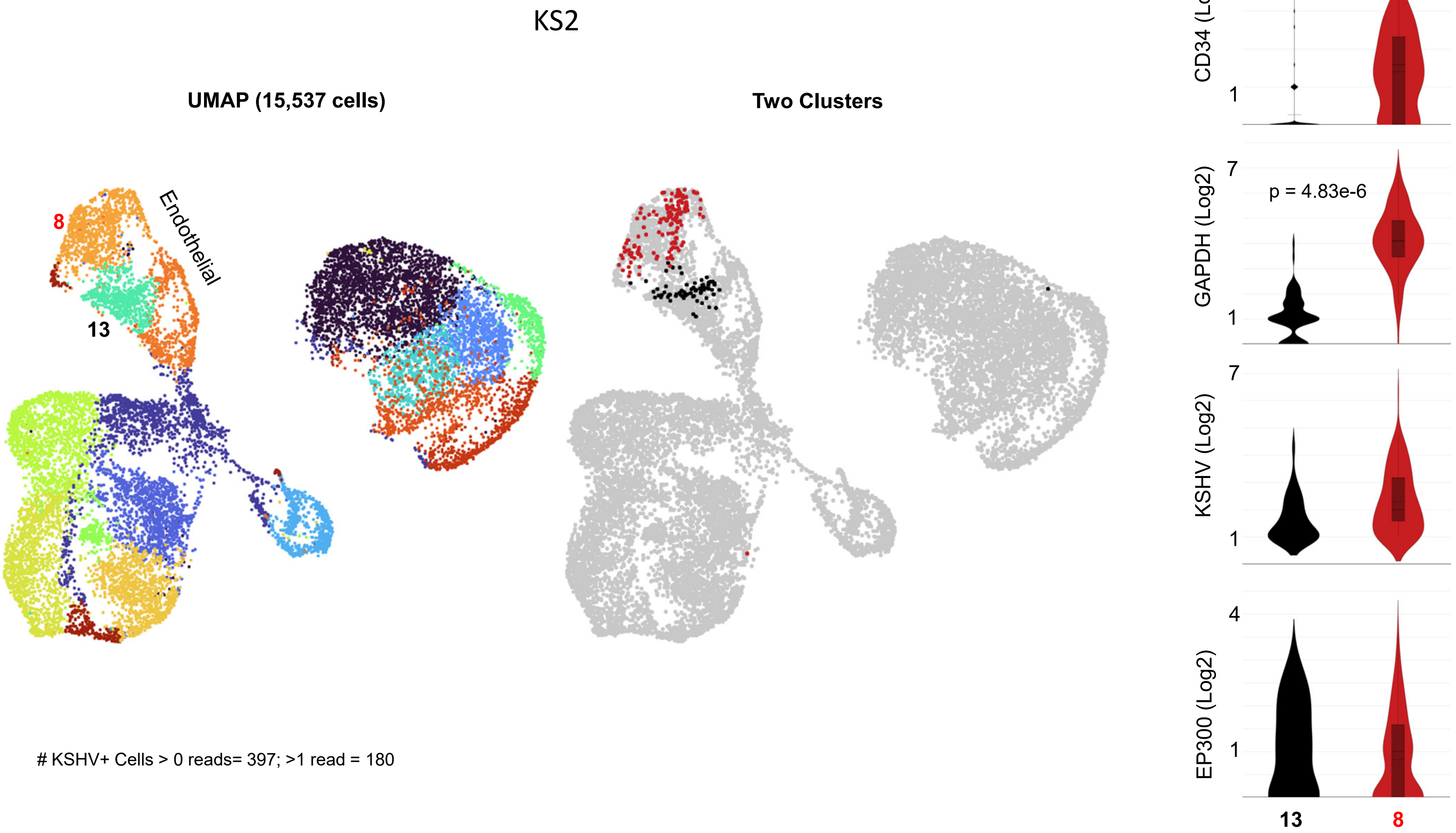
KS2
UMAP (15,537 cells)
Two Clusters
8
Endothelial
13
KSHV+ Cells > 0 reads= 397; >1 read = 180
CD34 (Log2)
5
1
GAPDH (Log2)
7
p = 4.83e-6
1
KSHV (Log2)
7
1
EP300 (Log2)
4
1
13
8

FIGURE S4D

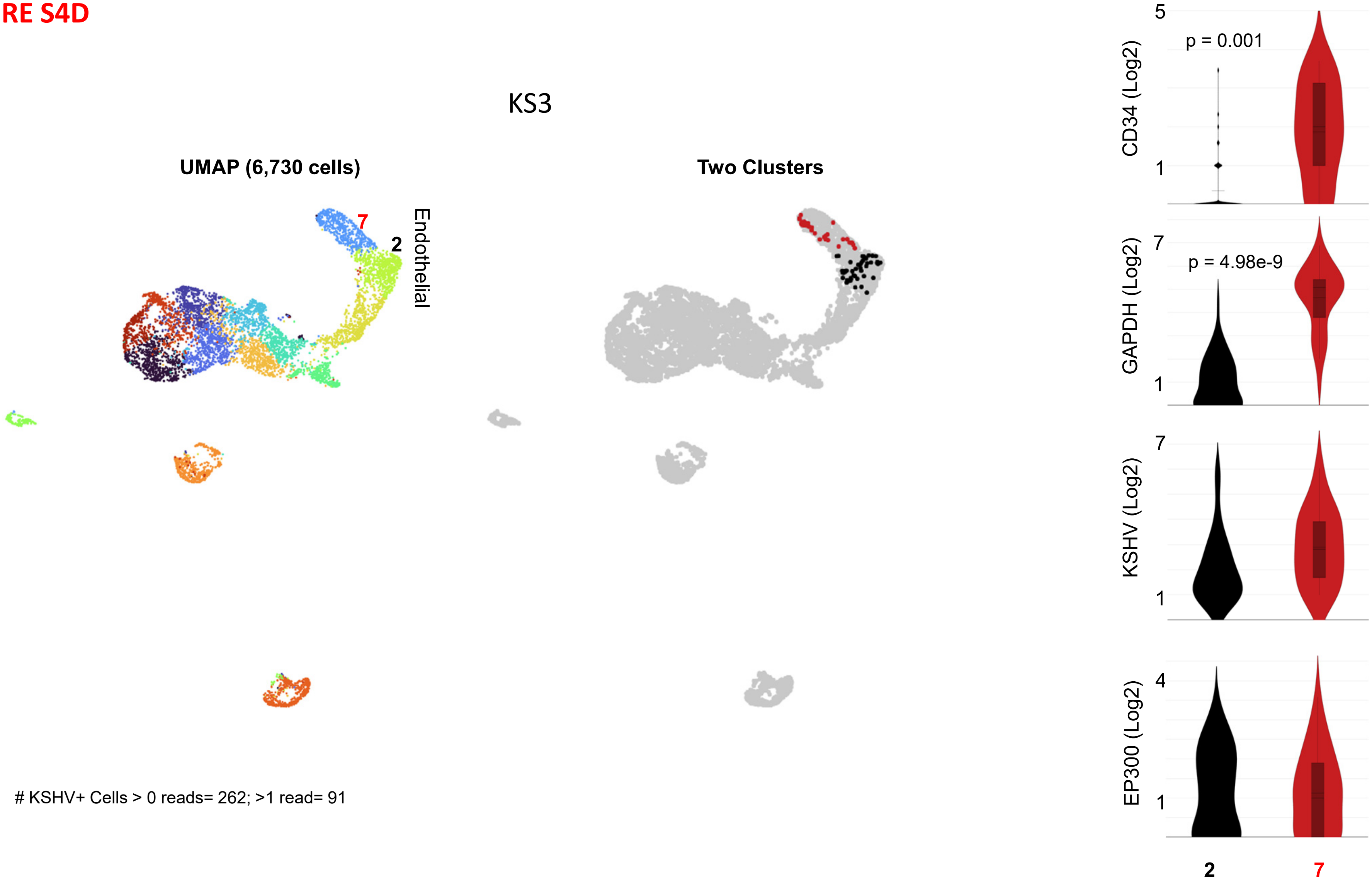

KS3
UMAP (6,730 cells)
7
2
Endothelial
Two Clusters
KSHV+ Cells > 0 reads= 262; >1 read= 91
CD34 (Log2)
5
1
p = 0.001
GAPDH (Log2)
7
1
p = 4.98e-9
KSHV (Log2)
7
1
EP300 (Log2)
4
1
2
7

FIGURE S4E

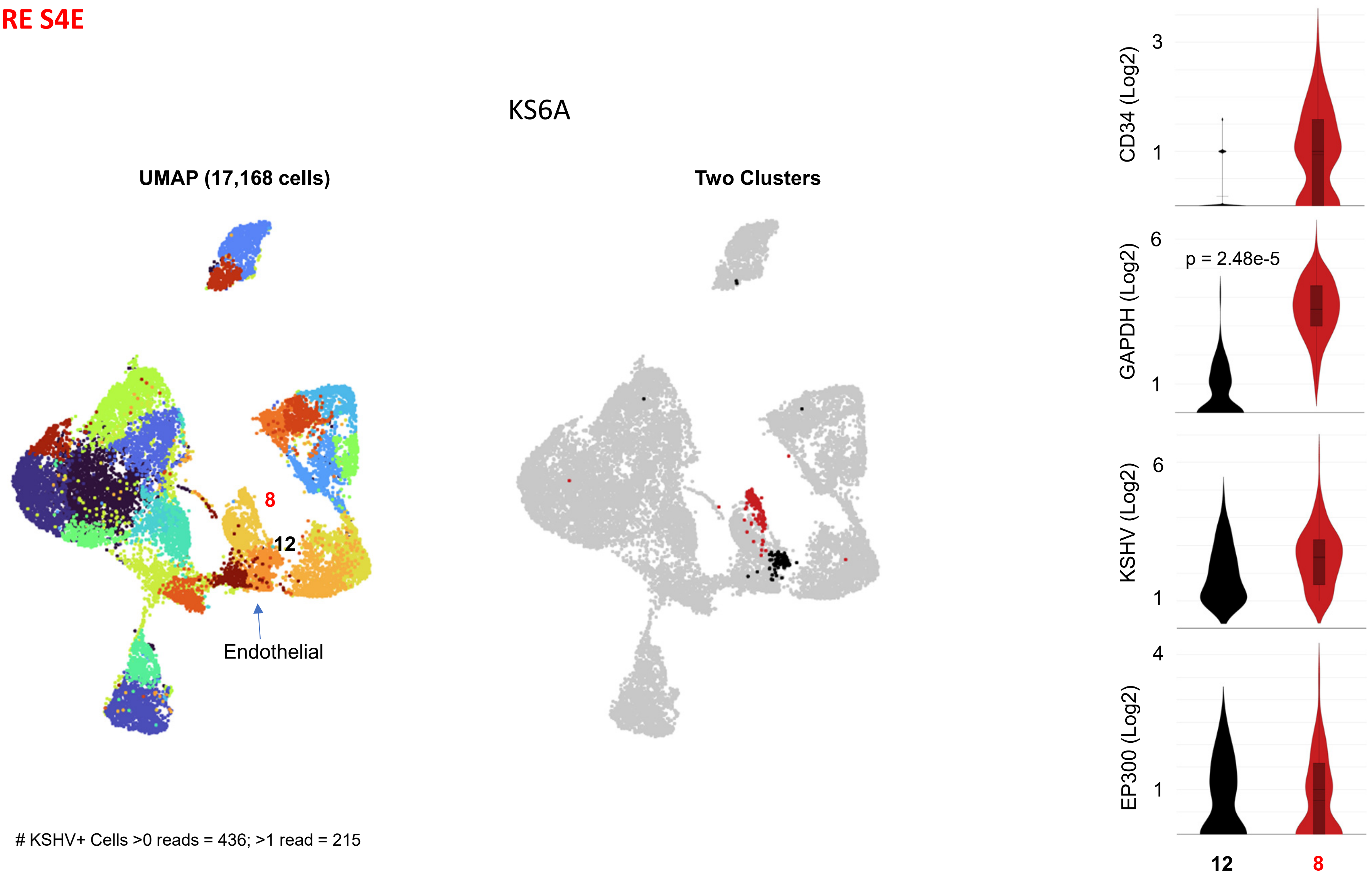

KSHV+ Cells >0 reads = 436; >1 read = 215

FIGURE S4F

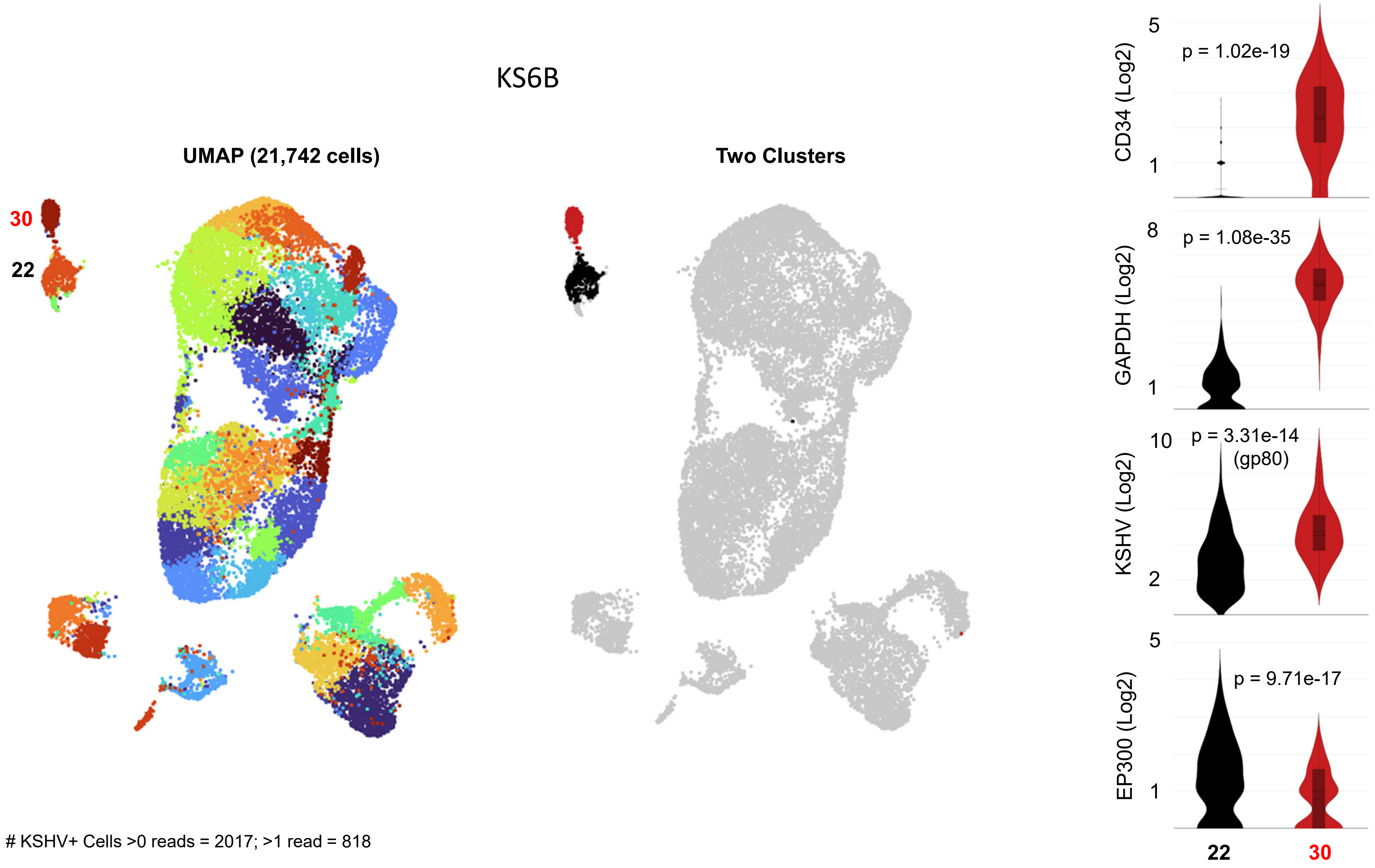
KS6B
UMAP (21,742 cells)
30
22
Two Clusters
KSHV+ Cells >0 reads = 2017; >1 read = 818
CD34 (Log2)
5
1
p = 1.02e-19
GAPDH (Log2)
8
1
p = 1.08e-35
KSHV (Log2)
10
2
p = 3.31e-14
(gp80)
EP300 (Log2)
5
1
p = 9.71e-17
22
30

FIGURE S4G

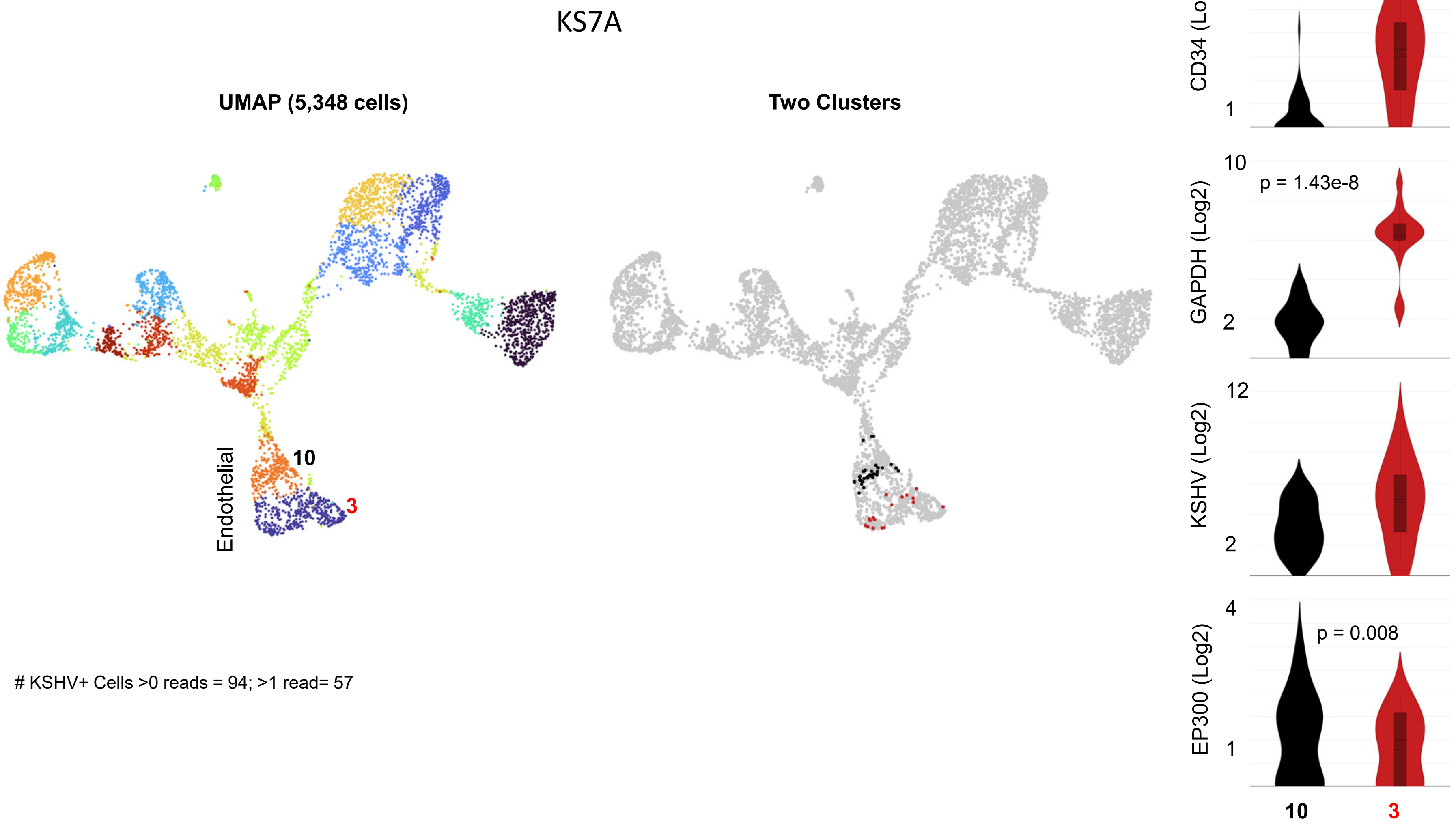

KSHV+ Cells >0 reads = 94; >1 read= 57

FIGURE S4H

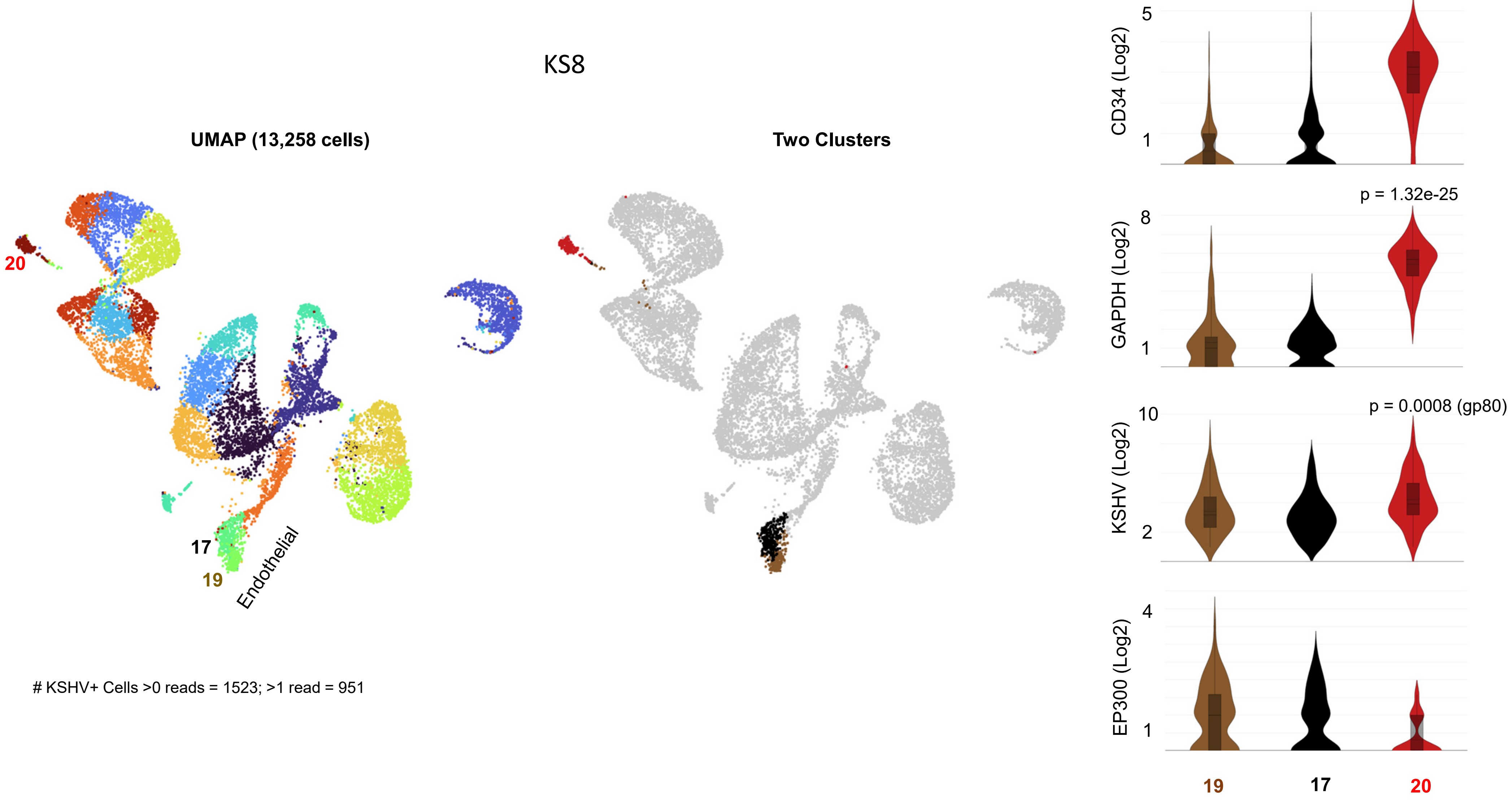
KS8
UMAP (13,258 cells)
Two Clusters
20
17
19
Endothelial
# KSHV+ Cells >0 reads = 1523; >1 read = 951
p = 4.02e-14
CD34 (Log2)
5
1
p = 1.32e-25
GAPDH (Log2)
8
1
p = 0.0008 (gp80)
KSHV (Log2)
10
2
EP300 (Log2)
4
1
19
17
20

FIGURE S4I

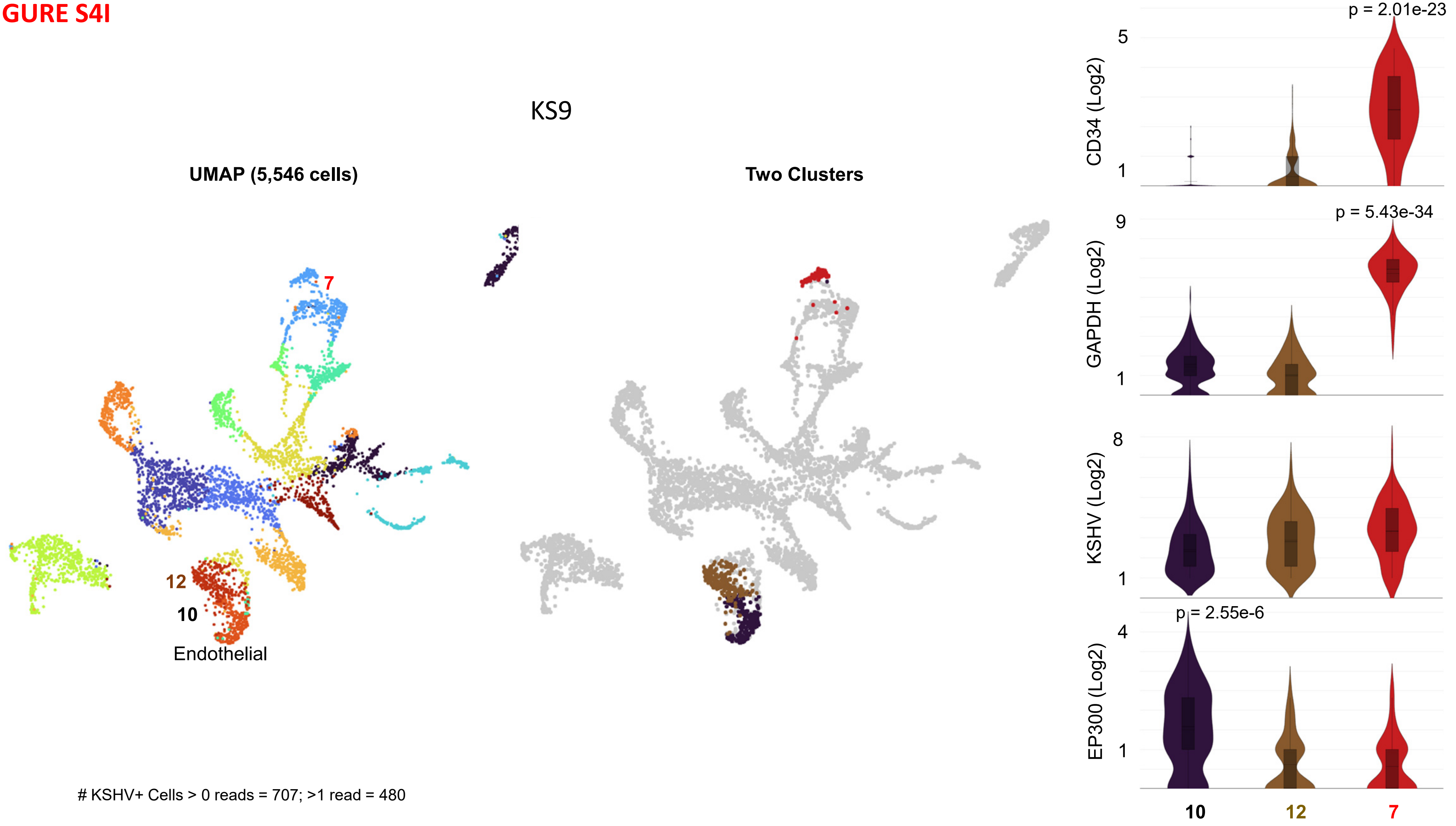
KS9
UMAP (5,546 cells)
7
12
10
Endothelial
KSHV+ Cells > 0 reads = 707; >1 read = 480
Two Clusters
p = 2.01e-23
5
1
CD34 (Log2)
p = 5.43e-34
9
1
GAPDH (Log2)
8
1
KSHV (Log2)
p = 2.55e-6
4
1
EP300 (Log2)
10
12
7

FIGURE S4J

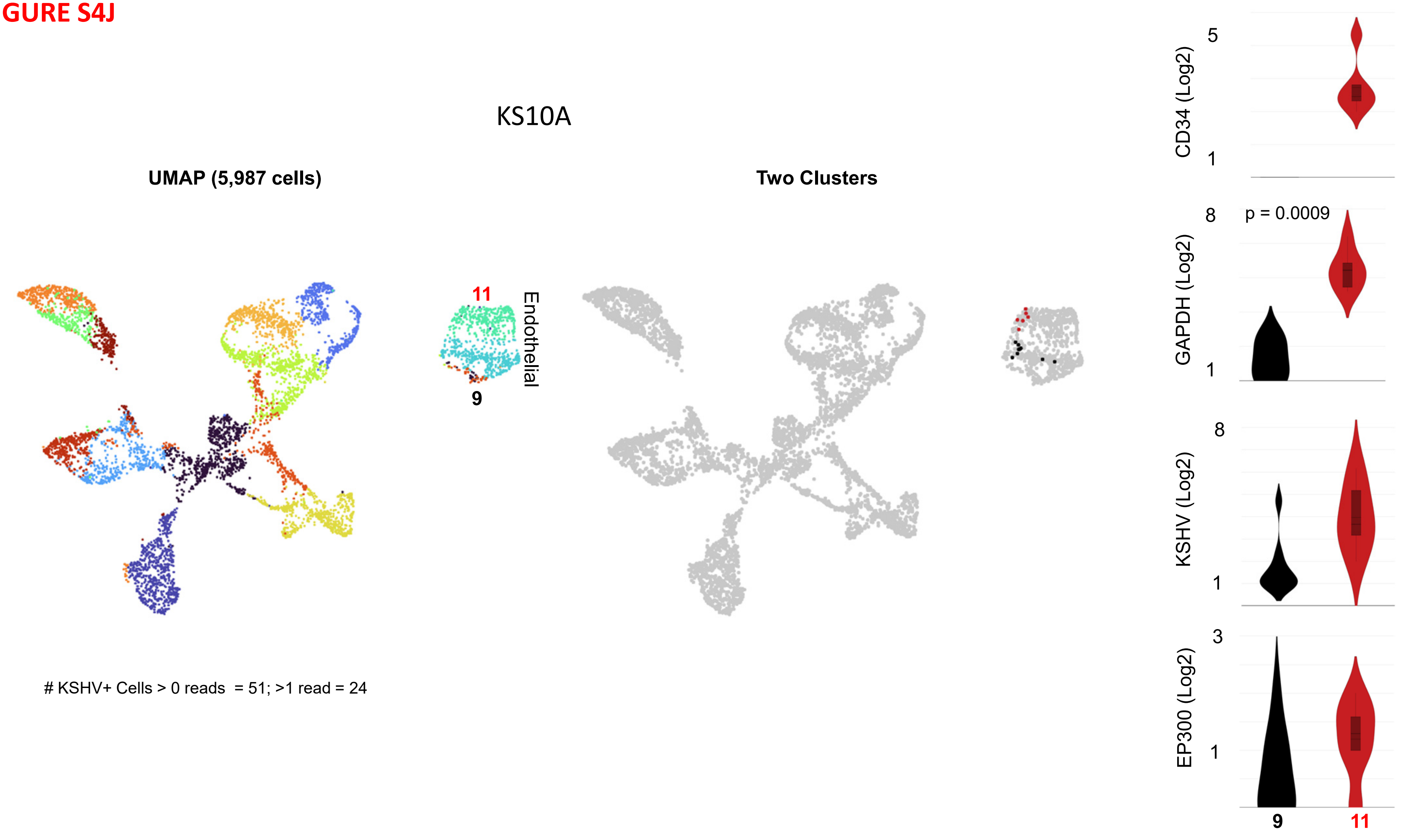

KS10A
UMAP (5,987 cells)
11
9
Endothelial
Two Clusters
KSHV+ Cells > 0 reads = 51; >1 read = 24
CD34 (Log2)
5
1
GAPDH (Log2)
8
1
p = 0.0009
KSHV (Log2)
8
1
EP300 (Log2)
3
1
9
11

FIGURE S4K

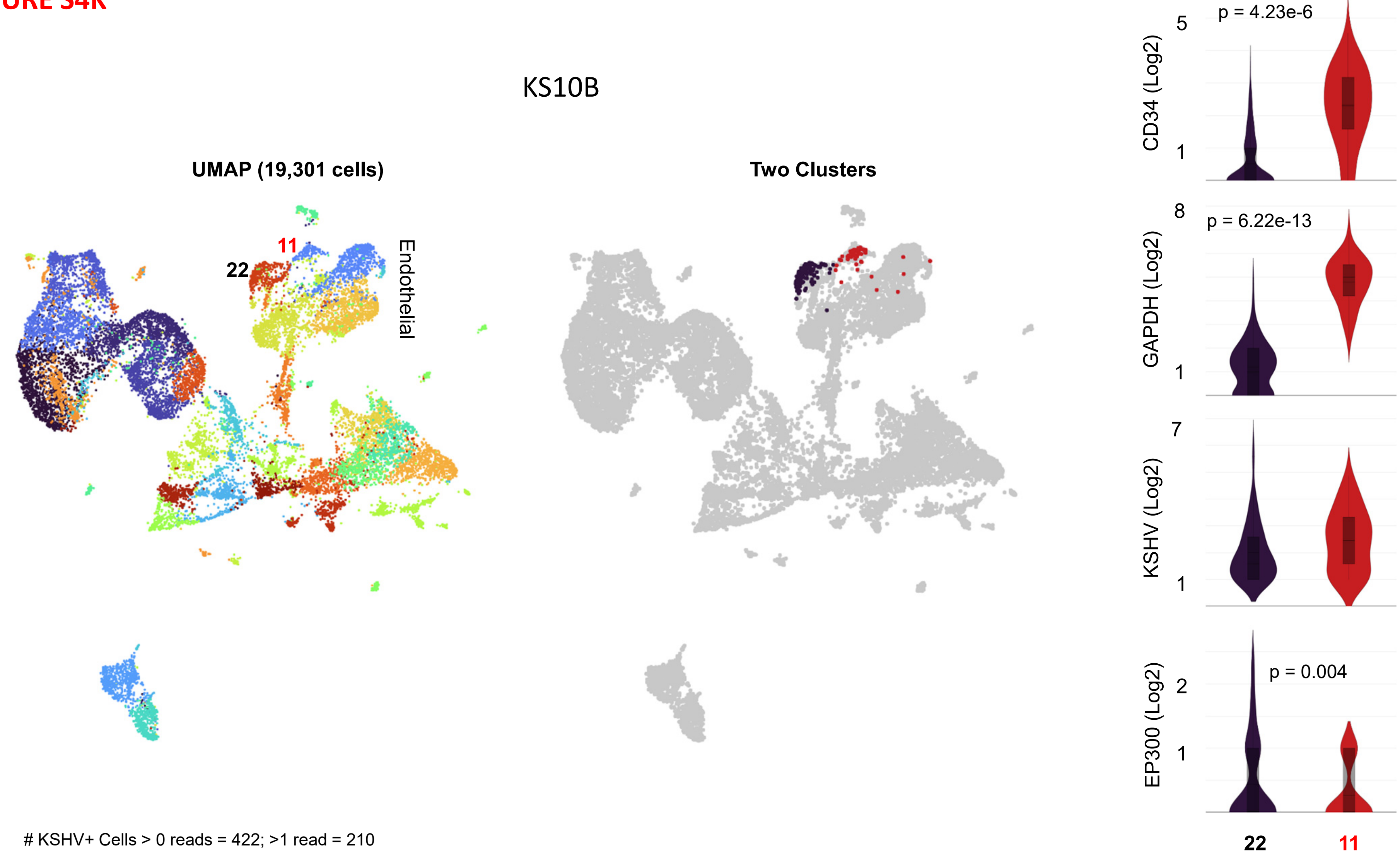

KSHV+ Cells > 0 reads = 422; >1 read = 210

FIGURE S4L

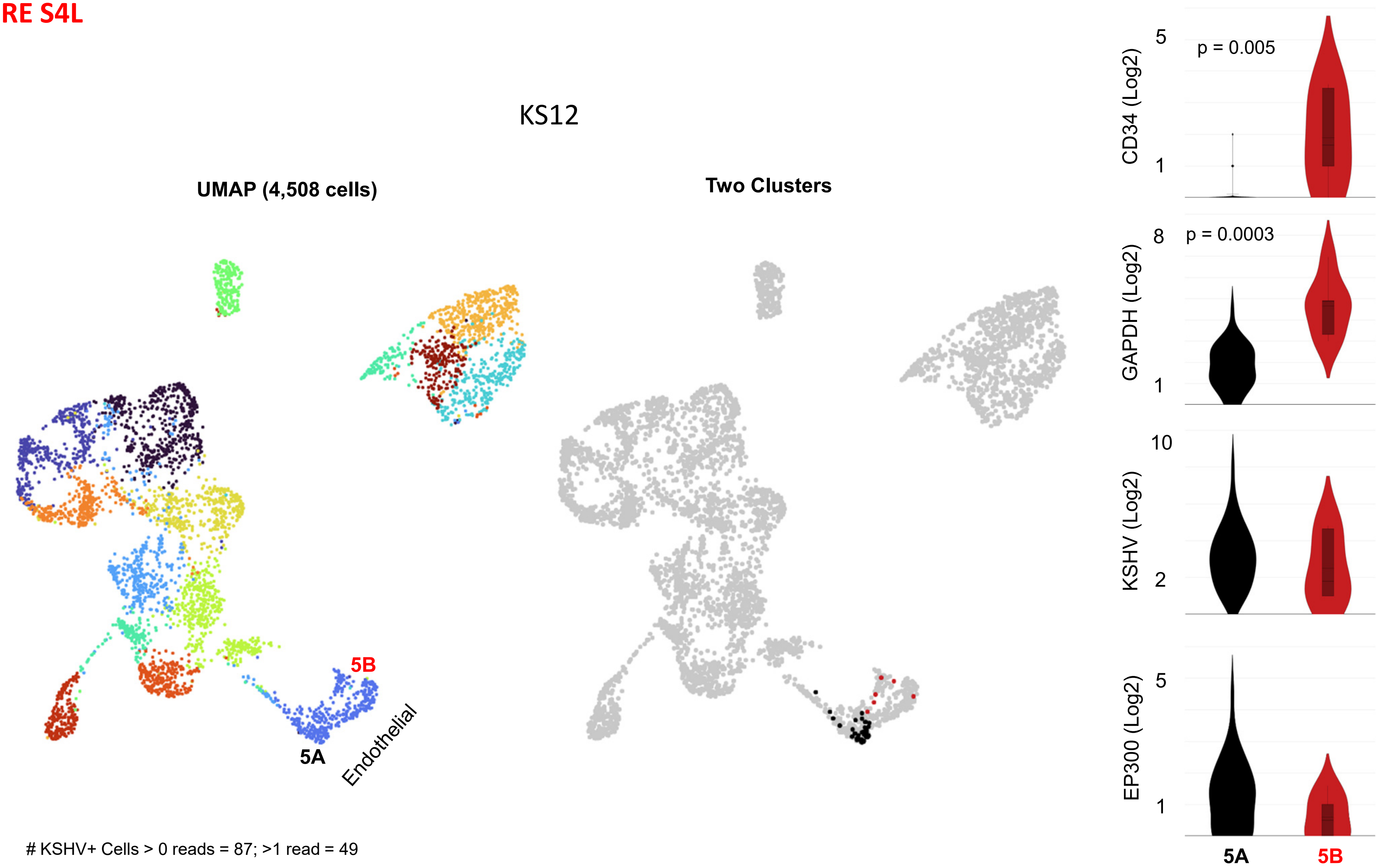

KS12
UMAP (4,508 cells)
5B
5A
Endothelial
Two Clusters
KSHV+ Cells > 0 reads = 87; >1 read = 49
CD34 (Log2)
5
1
p = 0.005
GAPDH (Log2)
8
1
p = 0.0003
KSHV (Log2)
10
2
EP300 (Log2)
5
1
5A
5B

FIGURE S4M

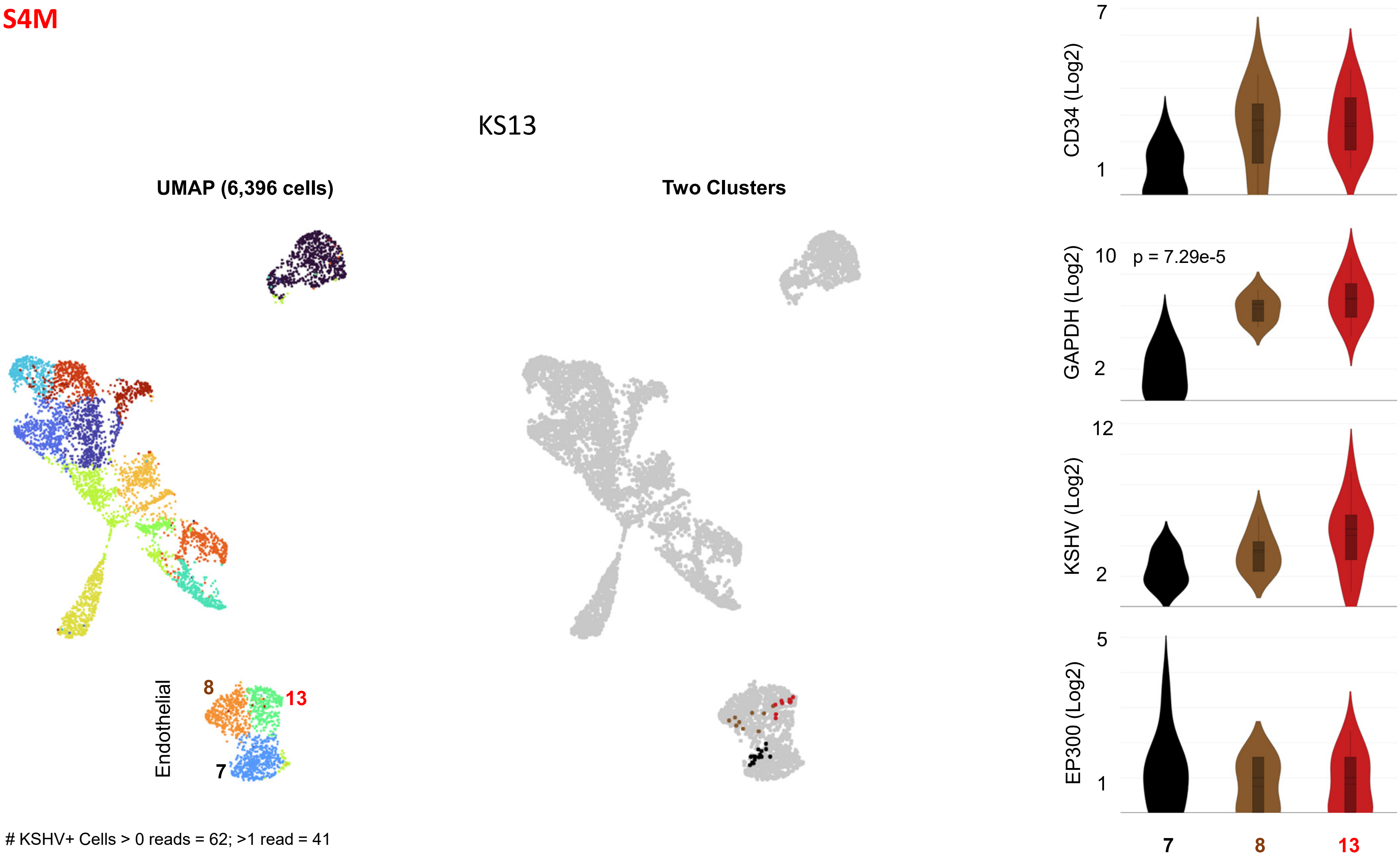
KS13
UMAP (6,396 cells)
Two Clusters
Endothelial
8
13
7
KSHV+ Cells > 0 reads = 62; >1 read = 41
CD34 (Log2)
7
1
GAPDH (Log2)
10
2
p = 7.29e-5
KSHV (Log2)
12
2
EP300 (Log2)
5
1
7
8
13

FIGURE S4N

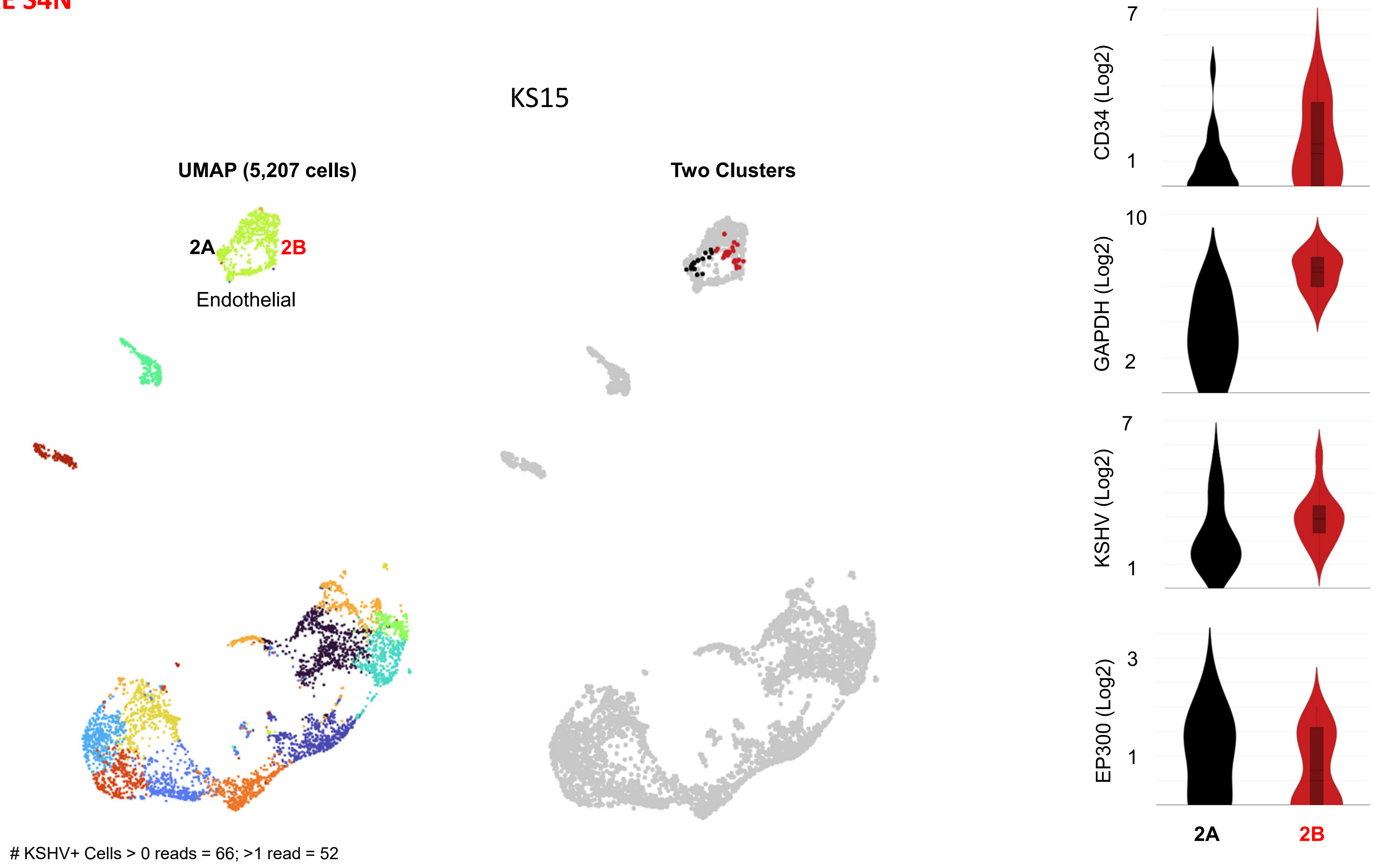
KS15
UMAP (5,207 cells)
2A
2B
Endothelial
Two Clusters
CD34 (Log2)
7
1
GAPDH (Log2)
10
2
KSHV (Log2)
7
1
EP300 (Log2)
3
1
2A
2B
KSHV+ Cells > 0 reads = 66; >1 read = 52

FIGURE S4O

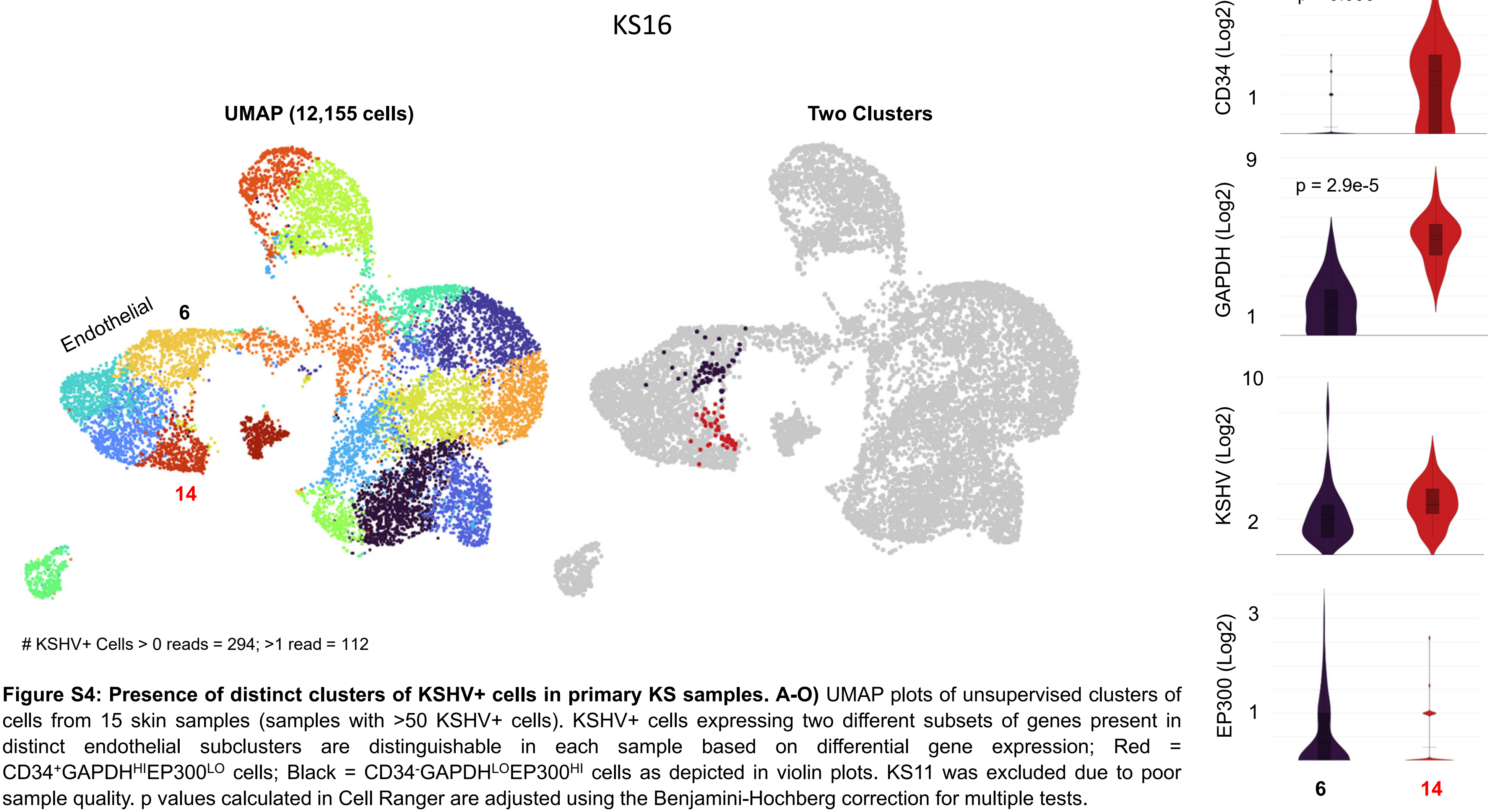

Figure S4: Presence of distinct clusters of KSHV+ cells in primary KS samples. A-O) UMAP plots of unsupervised clusters of cells from 15 skin samples (samples with >50 KSHV+ cells). KSHV+ cells expressing two different subsets of genes present in distinct endothelial subclusters are distinguishable in each sample based on differential gene expression; Red = $CD34^{+}GAPDH^{HI}EP300^{LO}$ cells; Black = $CD34^{-}GAPDH^{LO}EP300^{HI}$ cells as depicted in violin plots. KS11 was excluded due to poor sample quality. p values calculated in Cell Ranger are adjusted using the Benjamini-Hochberg correction for multiple tests.
