# Supplementary material for: Single-Cell Transcriptomic Analysis of Kaposi Sarcoma
Source: PLoS Pathog. 2025 Apr 1;21(4):e1012233. doi: 10.1371/journal.ppat.1012233 (PMC11984749; doi:10.1371/journal.ppat.1012233)
Supplement: S5 Fig — A) UMAP projections of KSHV+ Cluster 13 cells as defined in Fig 3 color coded based on Log2 expression of indicated endothelial markers. B) Left: Violin plots of Log2 total UMI count of all genes per cell for each graph-based t-SNE cluster in three KS samples (KS6B, KS8, KS9, all 3 samples with >2% KSHV positive cells) generated in Loupe Browser 8. Two subtypes of KSHV-infected cells are highlighted in each sample (with red circles and black squares) corresponding to graph-based unsupervised clusters containing CD34HI KSHV+ cells and CD34LO KSHV+ cells, respectively. Right: Violin plots of Log2 total UMI count per cell as well as Log 2 sum of lytic and latent gene expression in cells within each KSHV-infected cluster in KS8. p values calculated in Cell Ranger are adjusted using the Benjamini-Hochberg correction for multiple tests. C) t-SNE cluster plot of KS6B (1 of 3 samples with >2% KSHV positive cells) with CD34HI and CD34LO KSHV+ cell clusters highlighted. Differential expression of GAPDH in CD34HI and CD34LO KSHV+ cells graphed based on percent of GAPDH positive cells vs number of GAPDH reads per cell with table indicating the average number of GAPDH reads per cell in each group, the Log2 fold change, and the p value. D) a) t-SNE plots of KS6B, KS8, and KS9 skin samples (all 3 samples with >2% KSHV positive cells) with Log2 sum gene expression of latent KSHV genes (top) and lytic KSHV genes (bottom) in each cluster. b) Corresponding volcano plot depicts ratio of Log2 fold change between CD34HI and CD34LO KSHV+ cells vs. p-value (using paired t-test) to compare average reads per cell for selected human and KSHV genes for KS6B, KS8, KS9. c) Volcano plot comparing CD34HI and CD34LO KSHV+ cells from all samples indicating that the only viral genes significantly differentially expressed between the two clusters are K5 (in CD34HI cells) and K12 (in CD34LO cells). E) Genes expressed in high endothelial venules (HEVs) in mice are also significantly enriched in CD34HI K [file ppat.1012233.s005.pdf]

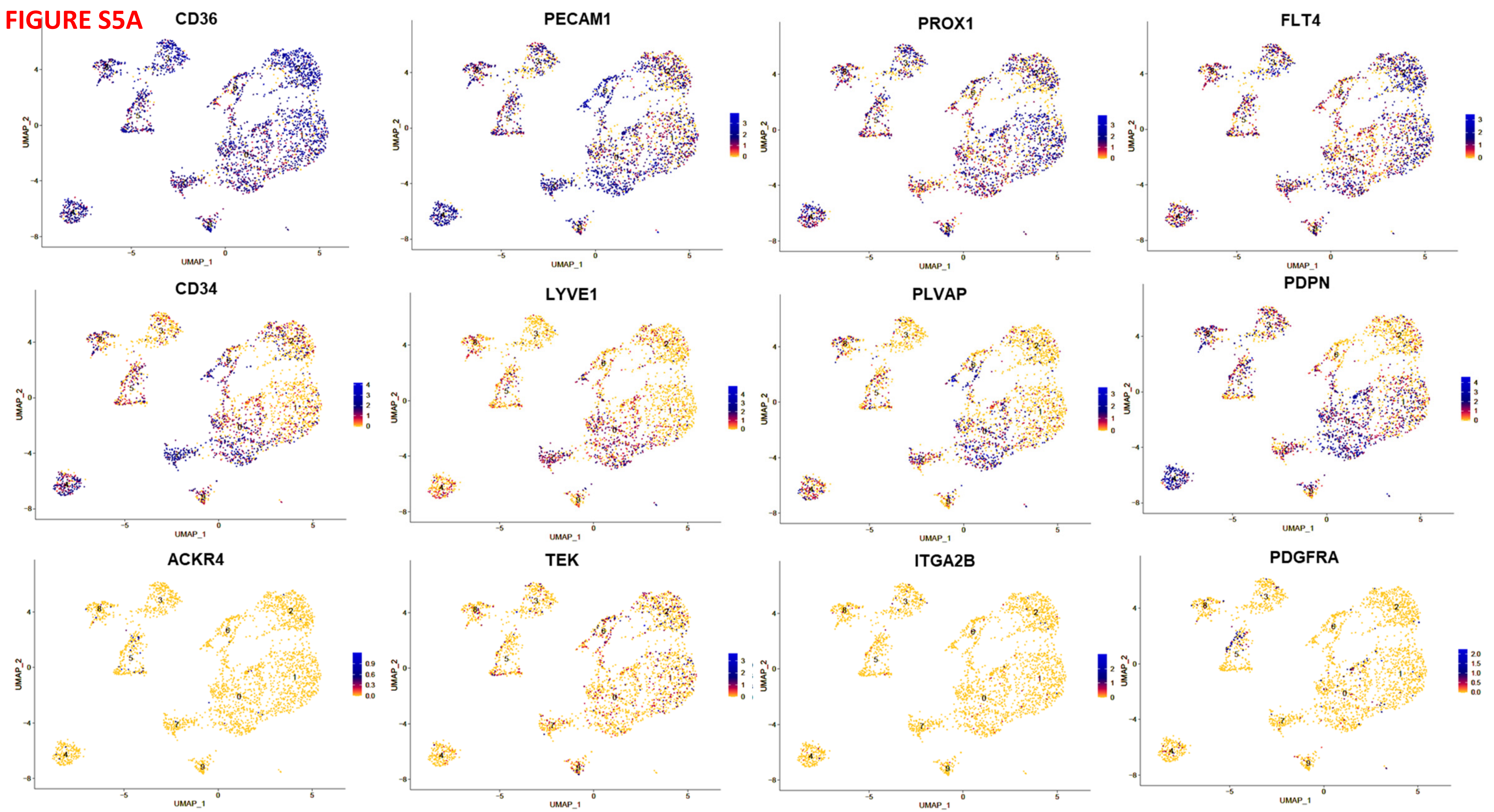

Figure S5A: Characteristics of CD34^{HI} and CD34^{LO} KSHV+ cells. A) UMAP projections of KSHV+ Cluster 13 cells as defined in Figure 3 color coded based on Log2 expression of indicated endothelial markers

FIGURE S5B

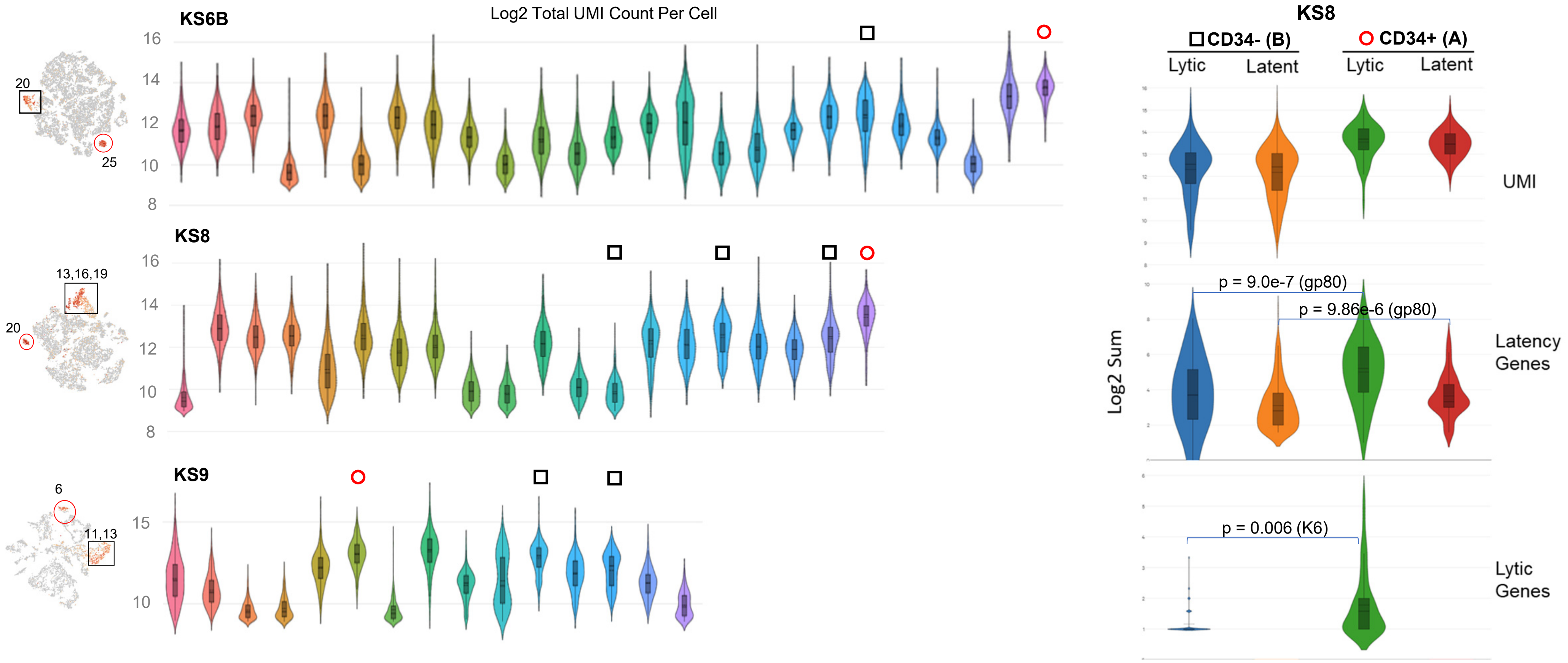

Figure S5B: Characteristics of CD34^{HI} and CD34^{LO} KSHV+ cells. Left: Violin plots of Log2 total UMI count of all genes per cell for each graph-based t-SNE cluster in three KS samples (KS6B, KS8, KS9) generated in Loupe Browser 8. Two subtypes of KSHV-infected cells are highlighted in each sample (with red circles and black squares) corresponding to graph-based unsupervised clusters containing CD34^{HI} KSHV+ cells and CD34^{LO} KSHV+ cells, respectively. Right: Violin plots of Log2 total UMI count per cell as well as Log 2 sum of lytic and latent gene expression in cells within each KSHV-infected cluster in KS8. p values calculated in Cell Ranger are adjusted using the Benjamini-Hochberg correction for multiple tests.

FIGURE S5C

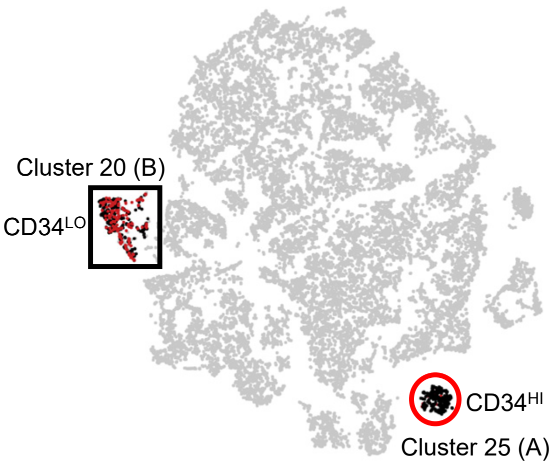

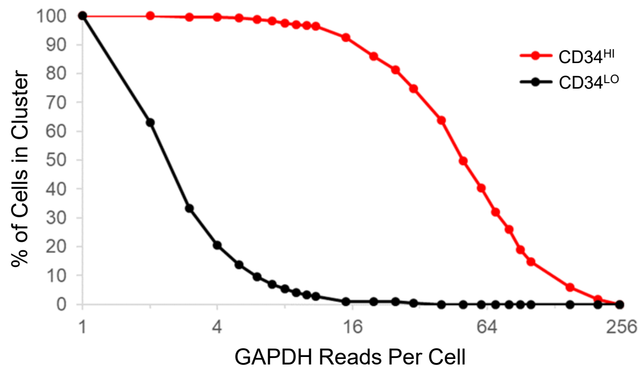

Gene	Avg CD34^{LO}	Avg CD34^{HI}	Log2	P-Value
GAPDH	3.09	44.42	3.85	8.39E-30

Figure S5C: Characteristics of CD34^{HI} and CD34^{LO} KSHV+ cells. t-SNE cluster plot of KS6B with CD34^{HI} and CD34^{LO} KSHV+ cell clusters highlighted. Differential expression of GAPDH in CD34^{HI} and CD34^{LO} KSHV+ cells graphed based on percent of GAPDH positive cells vs number of GAPDH reads per cell with table indicating the average number of GAPDH reads per cell in each group, the Log2 fold change, and the p value

FIGURE S5D

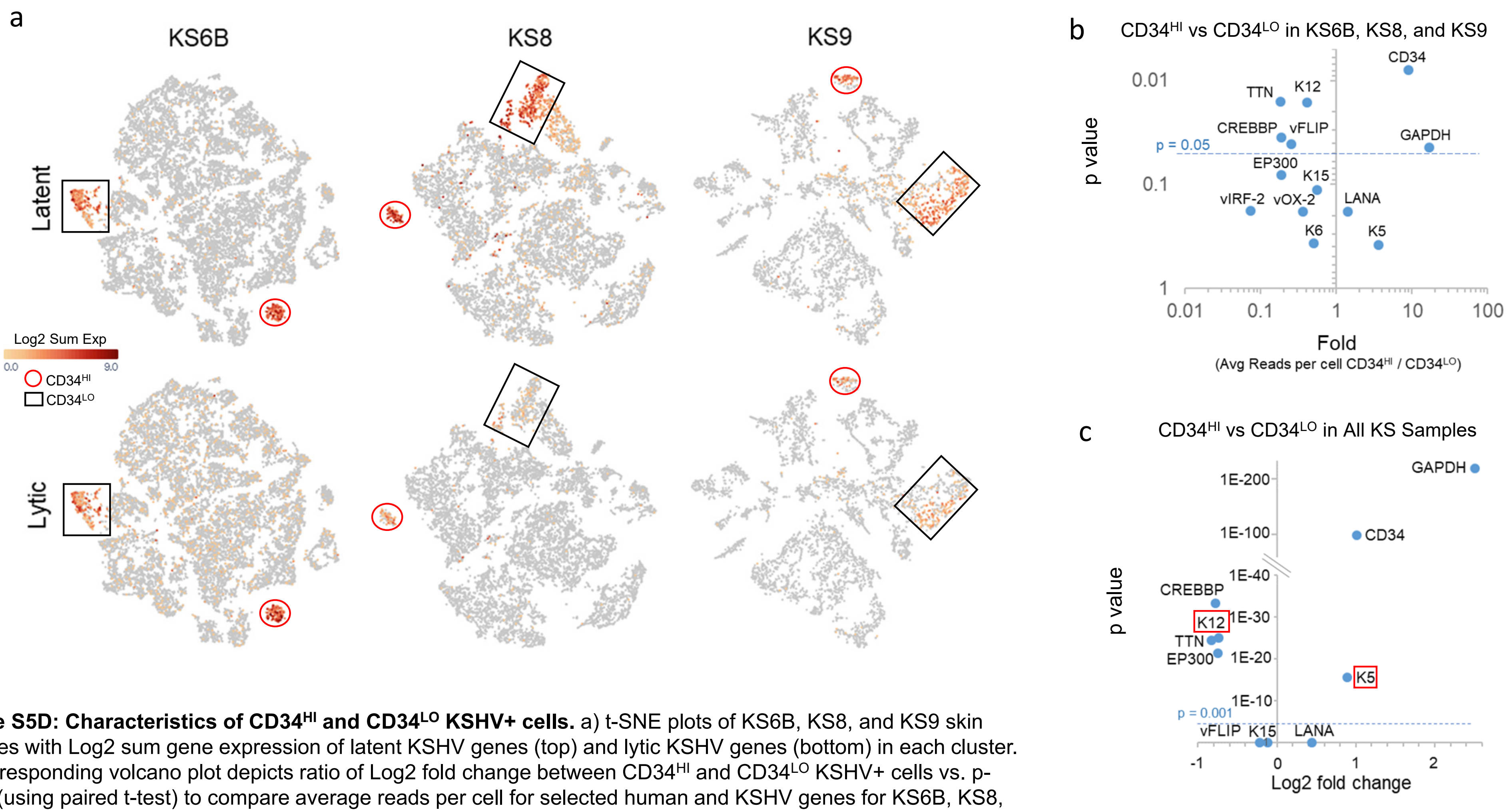

Figure S5D: Characteristics of CD34^{HI} and CD34^{LO} KSHV+ cells. a) t-SNE plots of KS6B, KS8, and KS9 skin samples with Log2 sum gene expression of latent KSHV genes (top) and lytic KSHV genes (bottom) in each cluster. b) Corresponding volcano plot depicts ratio of Log2 fold change between CD34^{HI} and CD34^{LO} KSHV+ cells vs. p-value (using paired t-test) to compare average reads per cell for selected human and KSHV genes for KS6B, KS8, KS9. c) Volcano plot comparing CD34^{HI} and CD34^{LO} KSHV+ cells from all samples indicating that the only viral genes significantly differentially expressed between the two clusters are K5 (in CD34^{HI} cells) and K12 (in CD34^{LO} cells).

FIGURE S5E

a

	p_val	avg_log2FC
CD34	2.03E-103	1.02
LGALS1	1.10E-218	2.52
IGFBP7	4.08E-232	2.50
CD63	2.51E-224	1.80
BSG	1.46E-173	1.21
LYVE1	1.47E-83	0.69
ICAM1	1.10E-36	0.69
FN1	1.56E-41	0.46
CXCL2	3.71E-10	0.46
SPARCL1	2.42E-14	0.42
ESAM	2.13E-17	0.34
F11R	5.83E-44	0.29
ST3GAL4	8.70E-59	0.28
ICAM2	5.03E-44	0.27
NT5E	1.32E-47	0.25
LTBR	5.24E-47	0.25
IL6ST	1.34E-20	0.21
F8	1.05E-19	0.12
LPAR6	3.87E-18	0.11
SDC4	8.32E-19	0.11

b

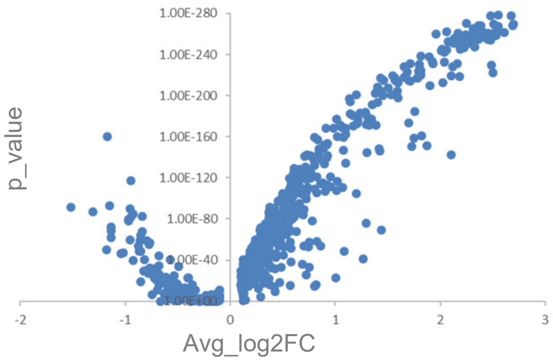

c

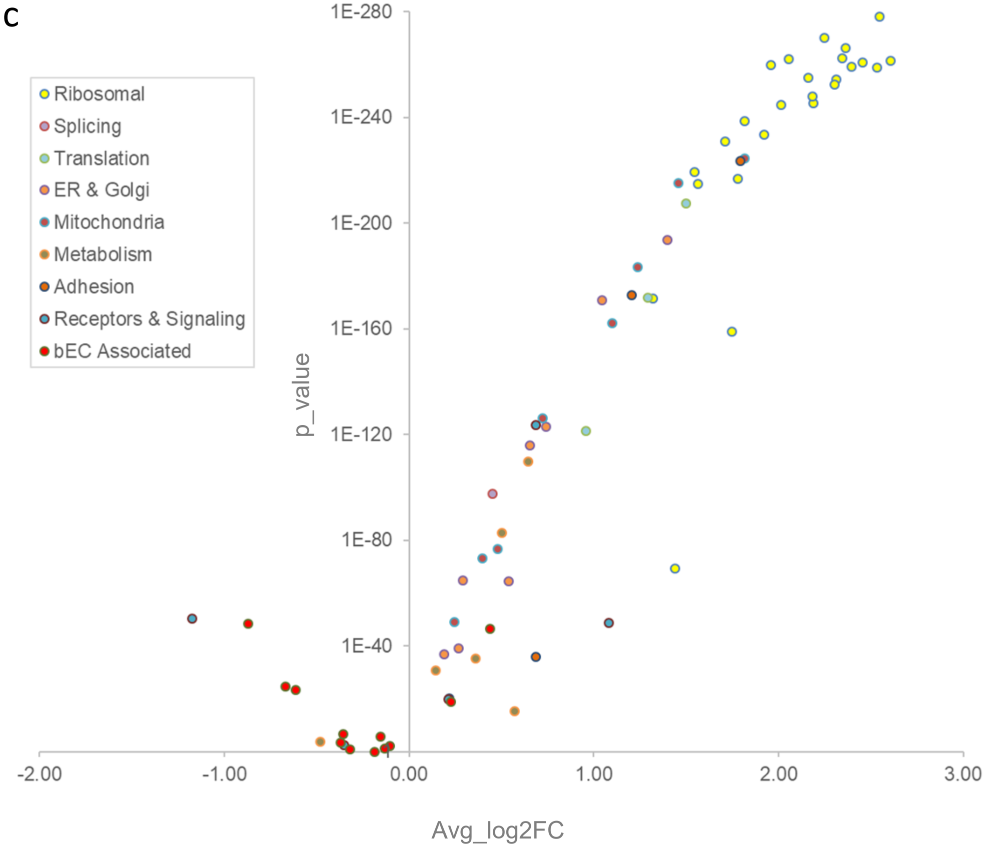

GENE	p_val	avg_log2FC
RPS12	3.56E-262	2.61
RPL26	5.66E-279	2.55
RPL39	1.65E-259	2.53
RPL19	1.03E-261	2.46
RPS4X	5.28E-260	2.40
RPL36	4.03E-267	2.36
RPS15	3.33E-263	2.34
RPLP0	5.05E-255	2.31
RPL34	2.80E-253	2.30
RPL11	5.66E-271	2.25
RPL12	4.41E-246	2.19
RPS5	8.79E-249	2.18
RPL18	6.51E-256	2.16
RPS16	7.08E-263	2.06
RPS3	1.23E-245	2.01
RPL10A	1.20E-260	1.96
RPL23A	2.97E-234	1.92
RPL36A	1.97E-239	1.82
RPSA	1.85E-217	1.78
RPS26	8.78E-160	1.75
RPL17	9.62E-232	1.71
RPL4	1.34E-215	1.57
RPS17	6.13E-220	1.54
RPS10	5.83E-70	1.44
RPS20	4.70E-172	1.32
SNRPE	2.64E-98	0.45
EEF1B2	3.11E-208	1.50
EEF1D	1.18E-172	1.29
EEF1G	6.54E-122	0.96
DAD1	1.65E-194	1.40
SEC61B	1.01E-171	1.05
VAMP5	1.08E-123	0.74
OSTC	1.29E-116	0.65
RPN2	3.24E-65	0.54
COPZ1	1.21E-65	0.29
SPCS2	6.53E-40	0.27
DDOST	1.67E-37	0.19
COX4I1	2.24E-225	1.81
SLC25A5	8.68E-216	1.46
PRELID1	4.36E-184	1.24
COX6C	6.41E-163	1.10
NDUFAB1	4.88E-127	0.72
NDUFB6	2.80E-77	0.48
FUNDC2	5.77E-74	0.40
NDUFA5	9.79E-50	0.25
ATP6V1G1	1.88E-110	0.64
PIGX	4.01E-16	0.57
SH3BGRL	1.25E-83	0.50
BACE2	8.46E-36	0.36
HEXB	1.72E-31	0.15
UGCG	1.68E-04	-0.48
CD63	2.51E-224	1.80
ICAM1	1.10E-36	0.69
TSPAN7	1.01E-20	0.22
BSG	1.46E-173	1.21
IER3	1.66E-49	1.08
SSR2	2.70E-124	0.69
IL6ST	1.34E-20	0.21
ARRB1	2.64E-03	-0.35
THSD7A	5.73E-51	-1.17
FLT4	2.18E-25	-0.67
CD36	3.71E-49	-0.87
SOX17	2.61E-47	0.44
EDNRB	1.44E-19	0.23
KDR	9.63E-03	-0.11
NOTCH4	6.83E-02	-0.13
NRP1	2.56E-06	-0.16
ITGA1	9.71E-01	-0.19
ITGA6	1.78E-01	-0.32
MEF2C	3.53E-07	-0.36
SOS1	4.87E-04	-0.37
SYNE2	5.41E-24	-0.61

Figure S5E: Genes expressed in High Endothelial Venules (HEVs) in mice are also significantly enriched in $CD34_{HI}$ KSHV+ cells.
Genes significantly enriched ($p < 0.01$) in the $CD34_{HI}$ population of KSHV+ Cluster 13 cells were compared to genes identified in a study by 2019 Veerman et al. (PMID: 30865898) describing scRNAseq analysis of high endothelial venules (HEVs) in mice. Table a lists 20 genes (including Log2 expression and p-value in $CD34^{HI}$ cells) that are also known to be expressed in HEVs (as described in Veerman et al.). The volcano plot in panel b indicates the log2 fold change and p value of 812 genes that are significantly different between $CD34^{HI}$ KSHV+ cells and $CD34^{LO}$ KSHV+ cells that are also significantly different between HEVs and blood endothelial cells (bECs) in Veerman et al. The volcano plot (depicting log2 fold change and p-value) in panel c shows that the majority of the genes differentially expressed in mouse HEVs in Veerman et al. are also significantly expressed in $CD34^{HI}$ KSHV+ cells compared to $CD34^{LO}$ KSHV+ cells (genes in the volcano plot are listed in the table along with p value and log2 fold change and color coded as shown it the legend of the volcano plot).

FIGURE S5F

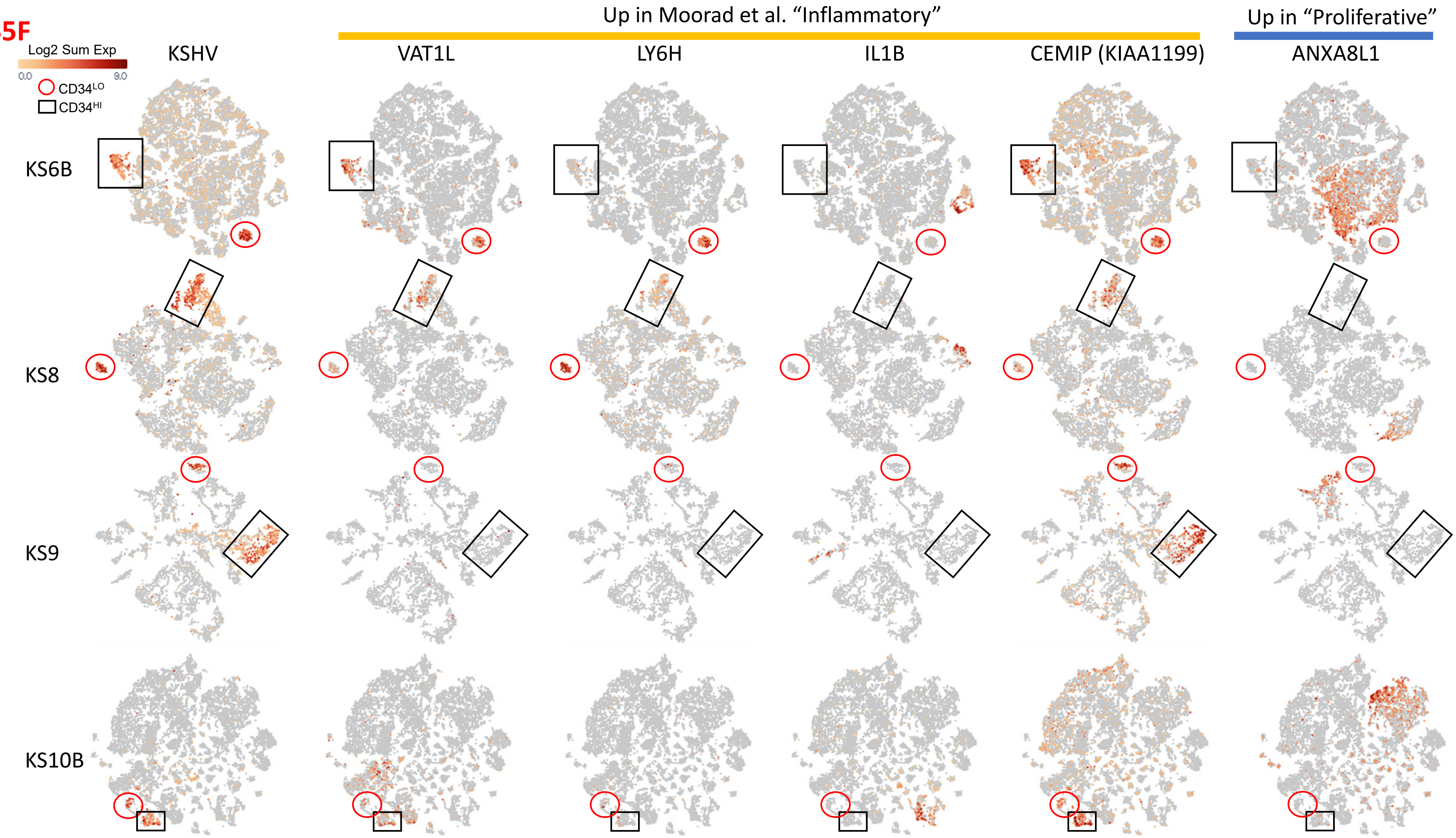

Figure S5F: Characteristics of CD34^{HI} and CD34^{LO} KSHV+ cells. t-SNE plots of selected KS samples indicating CD34^{HI} KSHV+ cells (red circles) and CD34^{LO} KSHV+ cells (black boxes) and highlighting genes (Log2 expression) identified by Moorad et al. as associated with inflammatory vs. proliferative KS lesions.

FIGURE S5G

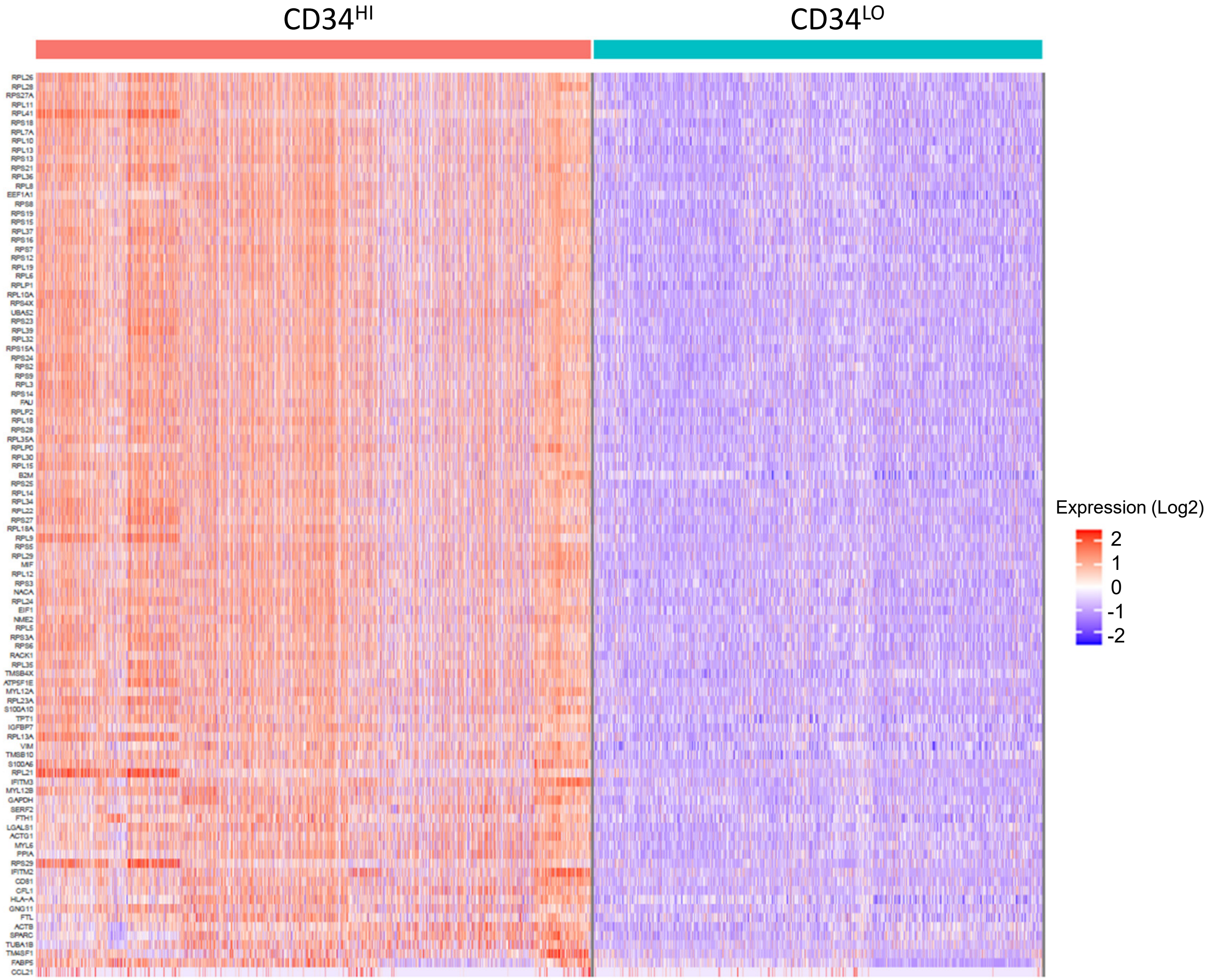

Figure S5G: Heat Map of Differential Gene Expression between CD34^{HI} and CD34^{LO} KSHV+ cells. 100 of 3,546 differentially expressed genes with p value < 0.01 are shown. All genes shown have p values < 1E-200).
