# Supplementary material for: Single-Cell Transcriptomic Analysis of Kaposi Sarcoma
Source: PLoS Pathog. 2025 Apr 1;21(4):e1012233. doi: 10.1371/journal.ppat.1012233 (PMC11984749; doi:10.1371/journal.ppat.1012233)
Supplement: S6 Fig — A graphical depiction of the KSHV latency cluster with a corresponding bam file viewed in IGV of a representative KS skin sample. Pink and blue bars represent reads mapped to the KSHV genome. Long blue horizontal lines represent reads that span splice junctions and correspond with blue arcs which quantitate splice junctions. The grey bar graph indicates read depth and the genome coordinates correspond to the NCBI KSHV reference genome GCF_000838265.1. In this sample, the LTd promoter is most active promoter in the Latency cluster as previously described in Rose et al (PMID: 30557332). (PDF) [file ppat.1012233.s006.pdf]

NEW FIGURE S6

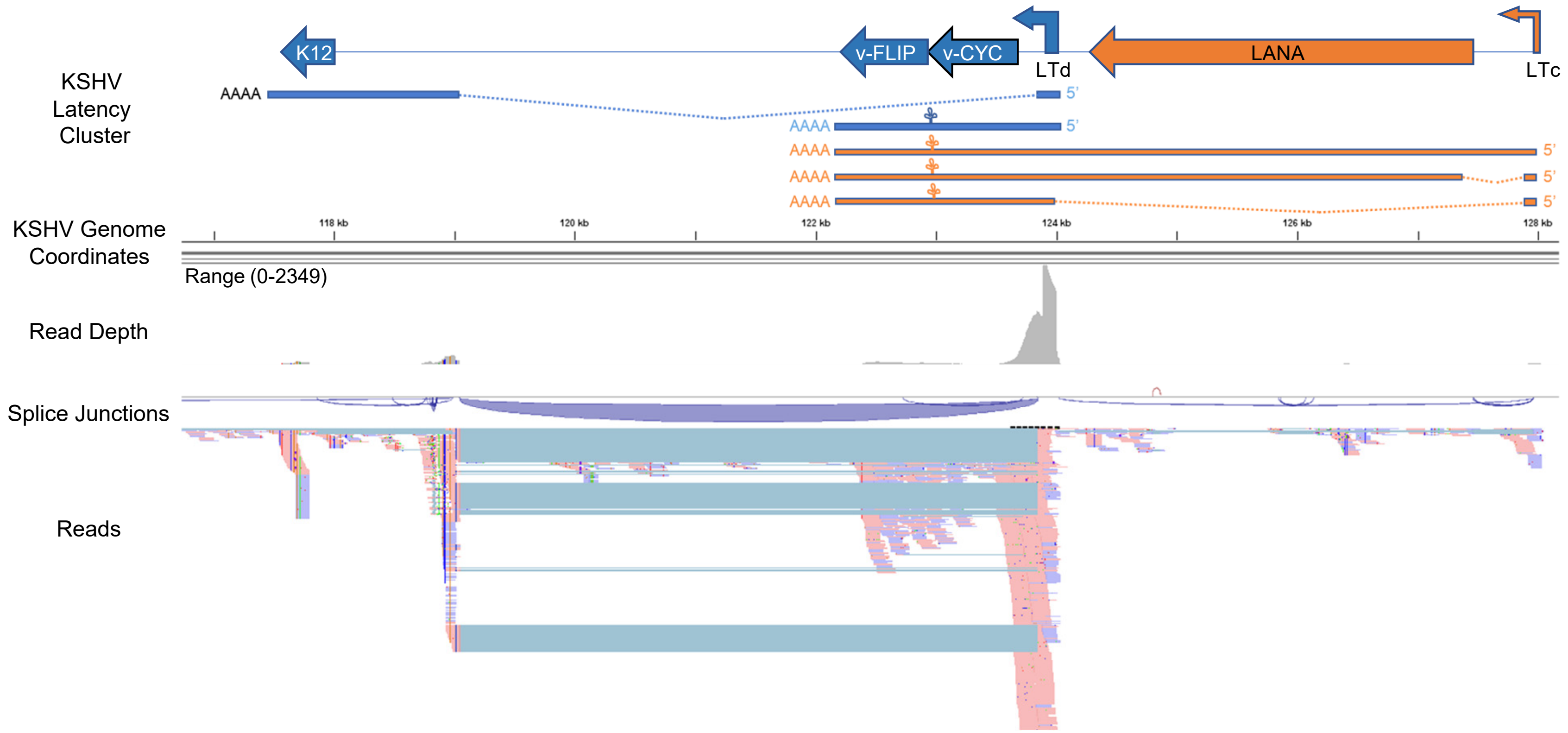

Figure S6: The KSHV Latency Cluster. A graphical depiction of the KSHV latency cluster with a corresponding bam file viewed in IGV of a representative KS skin sample. Pink and blue bars represent reads mapped to the KSHV genome. Long blue horizontal lines represent reads that span splice junctions and correspond with blue arcs which quantitate splice junctions. The grey bar graph indicates read depth and the genome coordinates correspond to the NCBI KSHV reference genome GCF_000838265.1. In this sample, the LTd promoter is most active promoter in the Latency cluster as previously described in Rose et al (PMID: 30557332).
