# Supplementary material for: Single-Cell Transcriptomic Analysis of Kaposi Sarcoma
Source: PLoS Pathog. 2025 Apr 1;21(4):e1012233. doi: 10.1371/journal.ppat.1012233 (PMC11984749; doi:10.1371/journal.ppat.1012233)
Supplement: S7 Fig — A) t-SNE plots of selected KS samples (KS8, 1 of 3 samples with >2% KSHV positive cells) showing unsupervised clustering and log2 expression of KSHV latent (Latency cluster) and lytic genes (K5) and highlighting the expression (purple = reads >1) of 14 voltage-gated sodium channel genes and the correlation between SCN9A and KSHV. B) t-SNE plots of selected KS samples (KS9, 1 of 3 samples with >2% KSHV positive cells) showing unsupervised clustering and log2 expression of KSHV latent (Latency cluster) and lytic genes (K5) and highlighting the expression (purple = reads >1) of 14 voltage-gated sodium channel genes and the correlation between SCN9A and KSHV. C) a) Violin plots of SCN9A expression and KSHV gene expression in each cluster of the graph-based t-SNE plot of KS6B. b) t-SNE plots of three KS samples (KS6B, KS8, KS9, all 3 samples with >2% KSHV positive cells), comparing the abundance, location, and expression of KSHV+ cells to SCN9A expressing cells (log2 scale). p values calculated in Cell Ranger are adjusted using the Benjamini-Hochberg correction for multiple tests. KSHV gene used for p value calculation is gp81. (PDF) [file ppat.1012233.s007.pdf]

FIGURE S7A

Figure S7A: Expression of voltage-gated sodium channel genes in KS. t-SNE plots of selected KS samples (KS8) showing unsupervised clustering and log2 expression of KSHV latent (Latency cluster) and lytic genes (K5) and highlighting the expression (purple = reads >1) of 14 voltage-gated sodium channel genes and the correlation between SCN9A and KSHV.

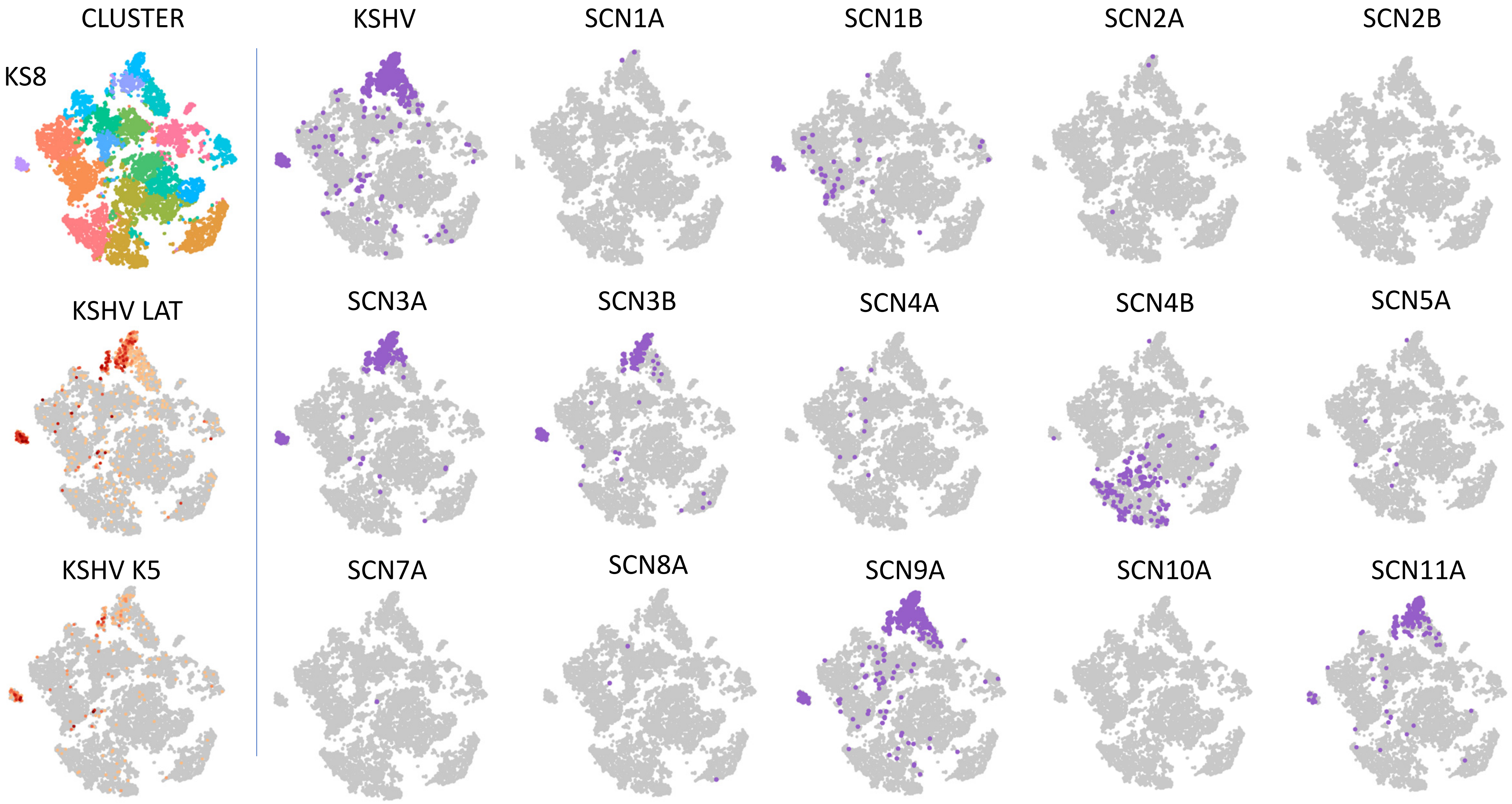

FIGURE S7B

Figure S7B: Expression of voltage-gated sodium channel genes in KS. t-SNE plots of selected KS samples (KS9) showing unsupervised clustering and log2 expression of KSHV latent (Latency cluster) and lytic genes (K5) and highlighting the expression (purple = reads >1) of 14 voltage-gated sodium channel genes and the correlation between SCN9A and KSHV.

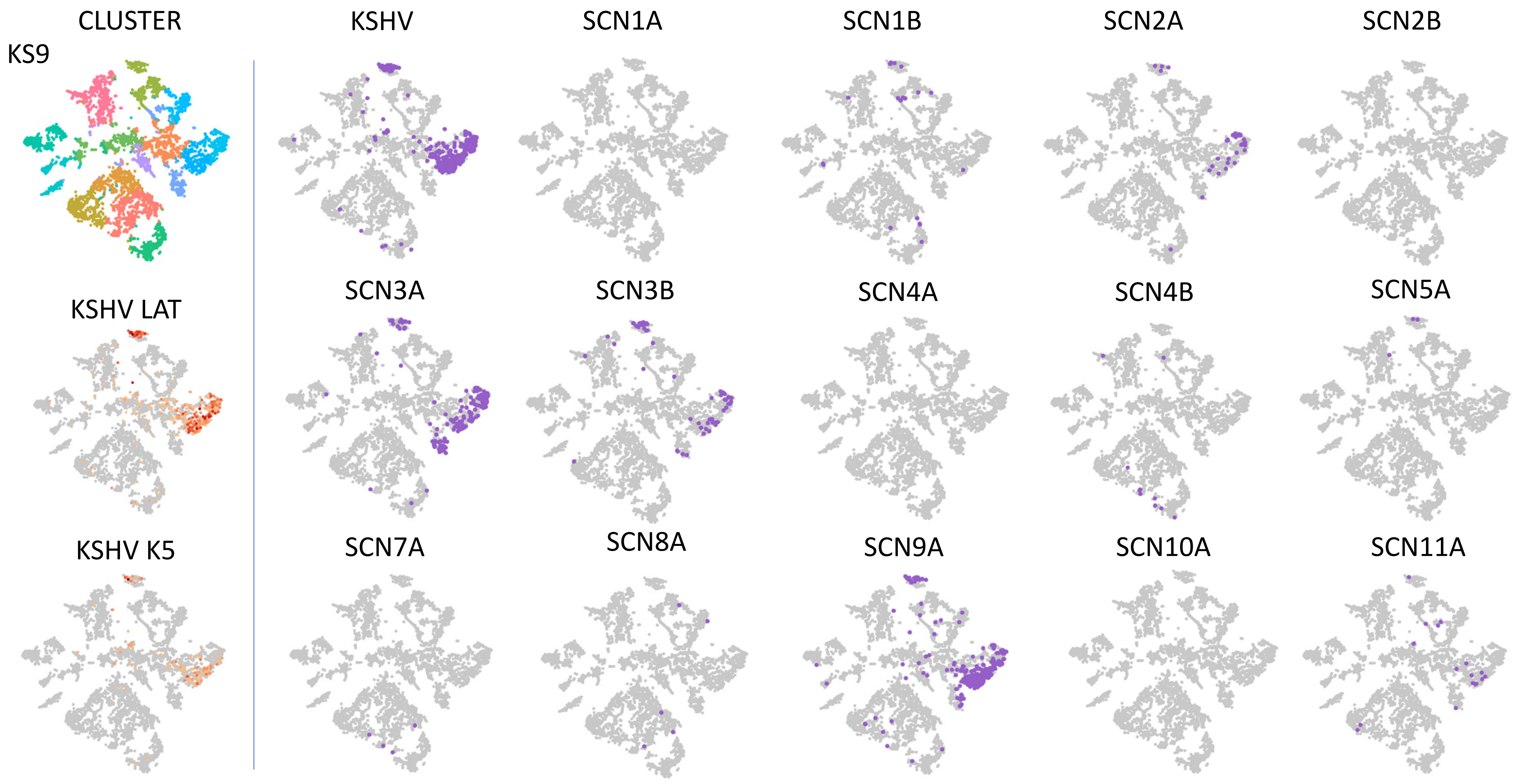

FIGURE S7C

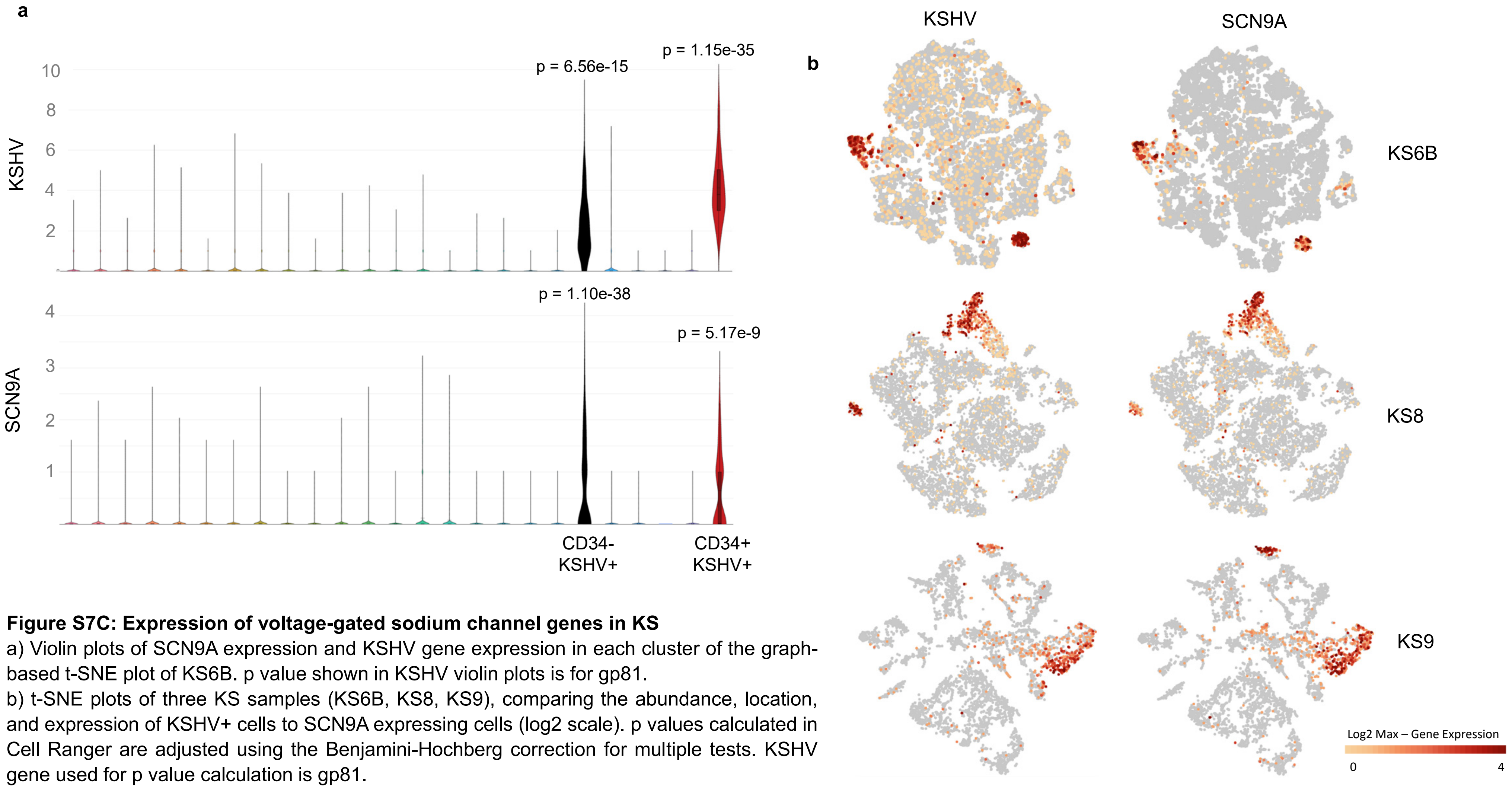

Figure S7C: Expression of voltage-gated sodium channel genes in KS

a) Violin plots of SCN9A expression and KSHV gene expression in each cluster of the graph-based t-SNE plot of KS6B. p value shown in KSHV violin plots is for gp81.

b) t-SNE plots of three KS samples (KS6B, KS8, KS9), comparing the abundance, location, and expression of KSHV+ cells to SCN9A expressing cells (log2 scale). p values calculated in Cell Ranger are adjusted using the Benjamini-Hochberg correction for multiple tests. KSHV gene used for p value calculation is gp81.
