# Supplementary material for: Single-Cell Transcriptomic Analysis of Kaposi Sarcoma
Source: PLoS Pathog. 2025 Apr 1;21(4):e1012233. doi: 10.1371/journal.ppat.1012233 (PMC11984749; doi:10.1371/journal.ppat.1012233)
Supplement: S8 Fig — Parts A-D show one section of a Kaposi sarcoma tissue block with SCN9A primary antibody used. Parts E-H show another section from the same Kaposi sarcoma tissue block, with isotype control antibody used instead of SCN9A primary antibody. Both sections show staining with Hoechst (for chromatin, A and E) and indirect immunofluorescence staining for LANA (B and F). LANA staining in both sections demonstrates expected variable nuclear staining with characteristic dot-like pattern in Kaposi sarcoma cells. SCN9A staining in the first tissue section (C) shows increased signal intensity compared to isotype control (G). D shows color overlay of LANA (red) and SCN9A (green), with increased green signal demonstrating SCN9A positivity as compared to the color overlay of LANA (red) and isotype control (absent) in H. (PDF) [file ppat.1012233.s008.pdf]

FIGURE S8

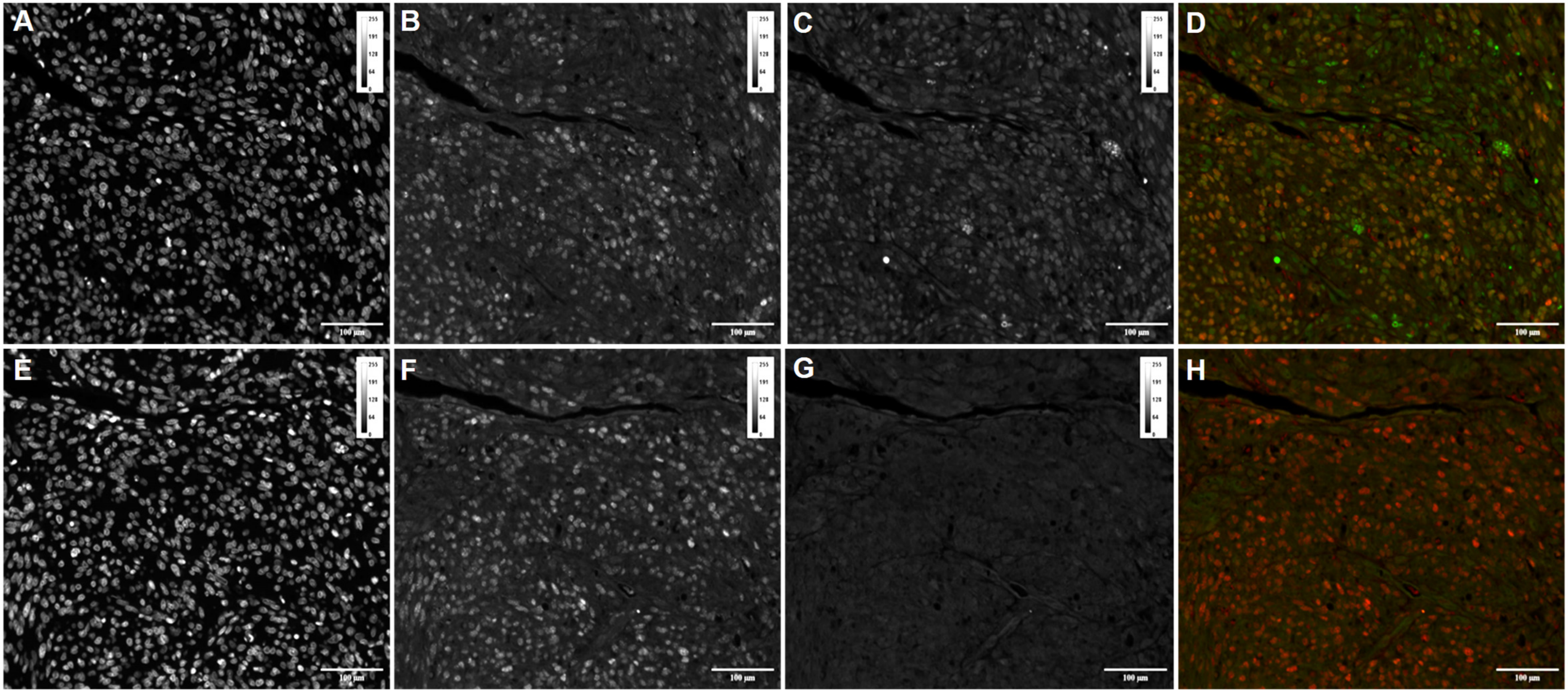

Figure S8 SCN9A expression in a case of Kaposi sarcoma. Parts **A-D** show one section of a Kaposi sarcoma tissue block with SCN9A primary antibody used. Parts **E-H** show another section from the same Kaposi sarcoma tissue block, with isotype control antibody used instead of SCN9A primary antibody. Both sections show staining with Hoechst (for chromatin, **A** and **E**) and indirect immunofluorescence staining for LANA (**B** and **F**). LANA staining in both sections demonstrates expected variable nuclear staining with characteristic dot-like pattern in Kaposi sarcoma cells. SCN9A staining in the first tissue section (**C**) shows increased signal intensity compared to isotype control (**G**). **D** shows color overlay of LANA (red) and SCN9A (green), with increased green signal demonstrating SCN9A positivity as compared to the color overlay of LANA (red) and isotype control (absent) in **H**.
