# Supplementary material for: Single-Cell Transcriptomic Analysis of Kaposi Sarcoma
Source: PLoS Pathog. 2025 Apr 1;21(4):e1012233. doi: 10.1371/journal.ppat.1012233 (PMC11984749; doi:10.1371/journal.ppat.1012233)
Supplement: S9 Fig — A) t-SNE plot of unsupervised clusters (K-means 7 clusters) for KS7 (1 of 3 samples with >2% KSHV positive cells) listing 9 genes used to identify Macrophages (CD14, CD80, CD86, MARCO, TLR2, IL10, IL1B, IL1A, ITGAX) being enriched (Log2 fold change and p-value indicated) in cluster 1 (black dots) and being similar to all cells that meet the criteria of Log2 Sum expression (>2) for all of the nine genes or Reads per cell (>3) for any of the nine genes. B) a) UMAPs of KS6B PBMC and KS10 PBMC (2 of 4 PBMC samples) with corresponding violin plots showing log2 expression of CD4, CD8A, CD3E (marker for T cells), and CD14 (marker for Macrophages). CD4, unlike CD8A is more highly expressed in CD14+ cells than CD3+. In panel b, CD4 expression in 4 of 4 evaluable PBMC samples is higher in monocytes than CD4+T cells when plotted as reads vs number of positive cells in each sample. (PDF) [file ppat.1012233.s009.pdf]

FIGURE S9A

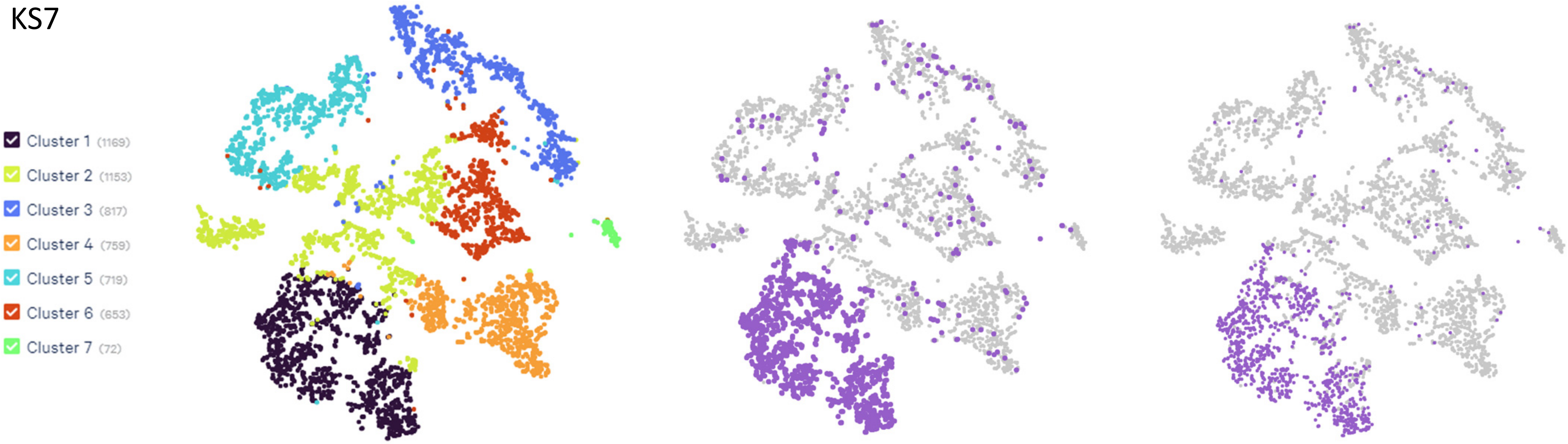

Unsupervised clusters; K-means (7 clusters)
Cluster 1 (black) (n=1,169)
Gene: Log2FC / P-value
- CD14: 5.22 / 4.03e-98
- CD80: 4.83 / 1.00e-106
- CD86: 4.51 / 3.52e-81
- TLR2: 5.47 / 1.63e-132
- MARCO: 5.48 / 4.69e-108
- IL10: 5.75 / 1.27e-119
- IL1B: 5.81 / 6.89e-151
- IL1A: 4.92 / 4.79e-82
- ITGAX: 4.28 / 9.74e-85

n=1,386
Log2 Sum Exp (>2) for all of the following:
CD14, CD80, CD86, CD11C (ITGAX),
MARCO, TLR2, IL1A, IL1B, IL10

n=1,144
Reads per cell (>3) for any of the following:
CD14, CD80, CD86, CD11C (ITGAX),
MARCO, TLR2, IL1A, IL1B, IL10

Figure S9A: Markers used to Identify Monocytes and Macrophages in scRNAseq Data. tSNE plot of unsupervised clusters (K-means 7 clusters) for KS7 listing 9 genes used to identify Macrophages (CD14, CD80, CD86, MARCO, TLR2, IL10, IL1B, IL1A, ITGAX) being enriched (Log2 fold change and p-value indicated) in cluster 1 (black dots) and being similar to all cells that meet the criteria of Log2 Sum expression (>2) for all of the nine genes or Reads per cell (>3) for any of the nine genes.

FIGURE S9B

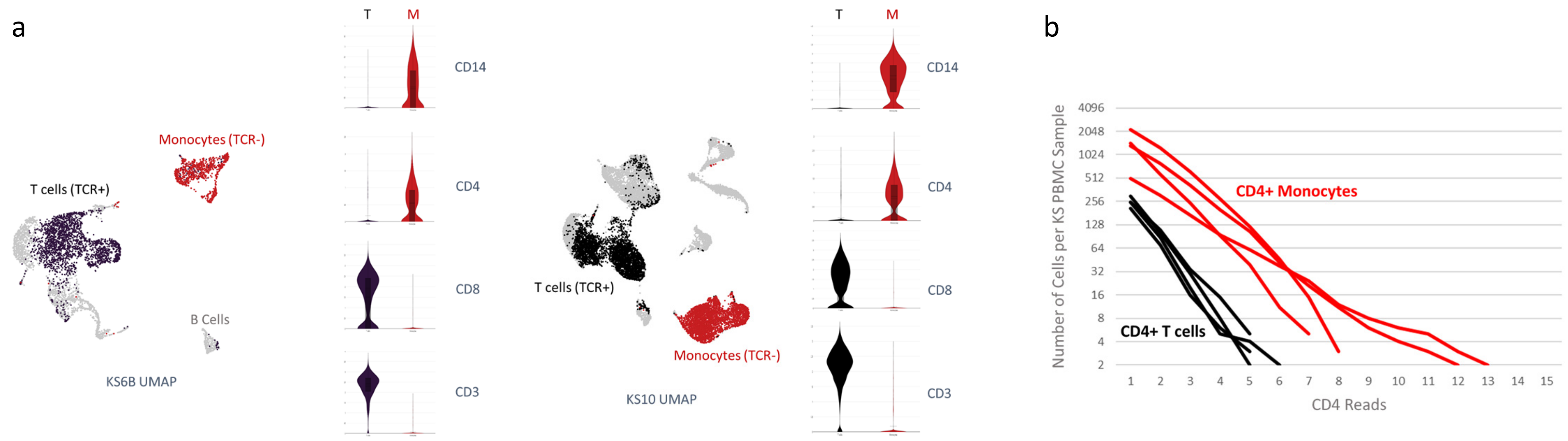

Figure S9B: Markers used to Identify Monocytes and Macrophages in scRNAseq Data. a) UMAPs of KS6B PBMC and KS10 PBMC with corresponding violin plots showing log2 expression of CD4, CD8A, CD3E (marker for T cells), and CD14 (marker for Macrophages). CD4, unlike CD8A is more highly expressed in CD14+ cells than CD3+. In panel b, CD4 expression in 4 of 4 evaluable PBMC samples is higher in monocytes than CD4+T cells when plotted as reads vs number of positive cells in each sample.
