# Supplementary material for: Single-Cell Transcriptomic Analysis of Kaposi Sarcoma
Source: PLoS Pathog. 2025 Apr 1;21(4):e1012233. doi: 10.1371/journal.ppat.1012233 (PMC11984749; doi:10.1371/journal.ppat.1012233)
Supplement: S10 Fig — 10X scRNAseq data for each sample was mapped against KSHV, HIV-1 and EBV genomes to generate BAM files. Reads mapping to unique barcodes were projected against composite UMAP objects of unsupervised clusters created in Seurat based on cells called by Cellranger as described in Fig 1. KSHV: 300K total KSHV reads map to 34,693 unfiltered barcodes. Filters applied for doublets, outliers, low quality cells, dying cells and a minimum of 2 KHSV transcripts dramatically reduce the number of cells defined as truly KSHV+ (red dots), also excluding 4 KSHV+ cells in PBMC samples (in KS10 PBMC and KS6A PBMC). HIV-1: 56 total reads mapped to 25 HIV-1+ barcodes in two samples (KS6A PBMC and KS10 PBMC) resulting in 23 unfiltered HIV-1+ cells 15 HIV+ cells after filtering (red dots). Reads in these 15 cells mapped almost exclusively to the viral LTRs. EBV: 222 total reads mapped to 100 EBV+ barcodes, resulting in 90 unfiltered EBV+ cells and 40 filtered EBV+ cells (red dots) all from the PBMC samples of KS6. (PDF) [file ppat.1012233.s010.pdf]

FIGURE S10

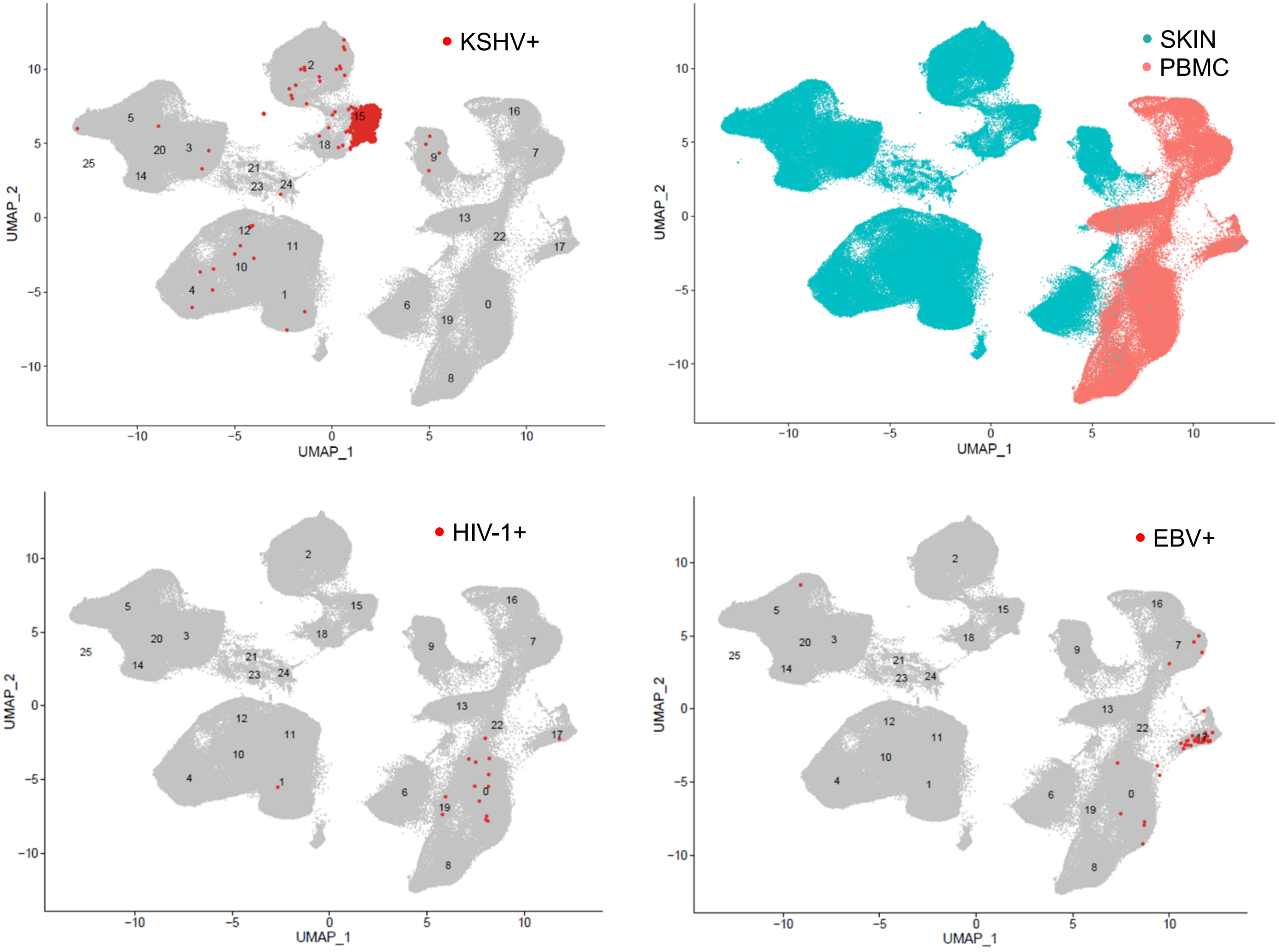

Figure S10: Viral reads in PMSC samples detected in BAM files.

10X scRNAseq data for each sample was mapped against KSHV, HIV-1 and EBV genomes to generate BAM files. Reads mapping to unique barcodes were projected against composite UMAP objects of unsupervised clusters created in Seurat based on cells called by Cellranger as described in Figure 1.

KSHV: 300K total KSHV reads map to 34,693 unfiltered barcodes. Filters applied for doublets, outliers, low quality cells, dying cells and a minimum of 2 KHSV transcripts dramatically reduce the number of cells defined as truly KSHV+ (red dots), also excluding 4 KSHV+ cells in PBMC samples (in KS10 PBMC and KS6A PBMC).

HIV-1: 56 total reads mapped to 25 HIV-1+ barcodes in two samples (KS6A PBMC and KS10 PBMC) resulting in 23 unfiltered HIV-1+ cells 15 HIV+ cells after filtering (red dots). Reads in these 15 cells mapped almost exclusively to the viral LTRs.

EBV: 222 total reads mapped to 100 EBV+ barcodes, resulting in 90 unfiltered EBV+ cells and 40 filtered EBV+ cells (red dots) all from the PBMC samples of KS6.
