# Supplementary material for: Single-Cell Transcriptomic Analysis of Kaposi Sarcoma
Source: PLoS Pathog. 2025 Apr 1;21(4):e1012233. doi: 10.1371/journal.ppat.1012233 (PMC11984749; doi:10.1371/journal.ppat.1012233)
Supplement: S11 Fig — Three t-SNE plots from three samples from patient KS10, a blood sample and two skin samples, one before and one after therapy. Seven CD8+T cell clones found in each of the three samples are indicated in seven different colors in the t-SNE plots that correspond to the color bar shown adjacent to the table. The number of cells representing each CD8+T cell clone is indicated by “count” and the relative abundance of each clone is indicated by “rank” with the most abundant CD8+T cell lone being #1. The most abundant CD8+T cell clone in the KS tumor (Blue dots) is the same in both skin samples and is also present in the peripheral blood but is the 10th most abundant T cell clone in the peripheral blood. Similarly the most abundant CD8+T cell clone in the peripheral blood (Red dots) is less frequently detected in the KS sample. These samples are from the only patient from who PBMC and 2 skin samples were obtained. (PDF) [file ppat.1012233.s011.pdf]

FIGURE S11

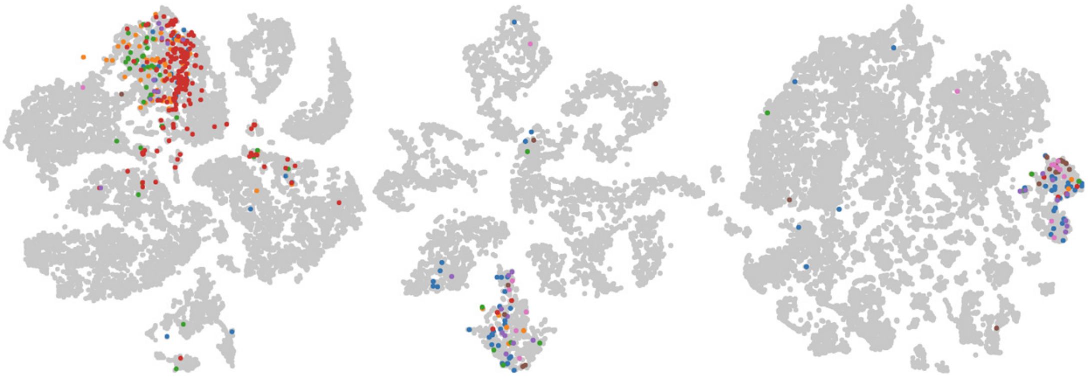

	KS10 PBMC		KS10 TUMOR PRE		KS10 TUMOR POST	
	Count	Rank	Count	Rank	Count	Rank
TRB: V7-9/J1-1	24	10	30	1	32	1
TRB: V6-5/J1-4	53	3	5	8	3	22 (tie)
TRB: V9/J2-1	37	6	6	4 (tie)	4	12(tie)
TRB: V4-1/J2-1	182	1	3	18 (tie)	4	12(tie)
TRB: V7-3/J2-5	10	26 (tie)	10	2	9	5 (tie)
TRB V3-1/J2-5	1	355(tie)	6	4 (tie)	18	2
TRB: V12-4/J1-1	1	355(tie)	6	4 (tie)	9	5 (tie)
Total	4262	2897	534	387	489	293

All listed are CD8+ TCR Clones

Figure S11: Expansion of CD8 T cell clones in primary KS. Three t-SNE plots from three samples from patient KS10, a blood sample and two skin samples, one before and one after therapy. Seven CD8+T cell clones found in each of the three samples are indicated in seven different colors in the t-SNE plots that correspond to the color bar shown adjacent to the table. The number of cells representing each CD8+T cell clone is indicated by "count" and the relative abundance of each clone is indicated by "rank" with the most abundant CD8+T cell lone being #1. The most abundant CD8+T cell clone in the KS tumor (Blue dots) is the same in both skin samples and is also present in the peripheral blood but is the 10th most abundant T cell clone in the peripheral blood. Similarly the most abundant CD8+T cell clone in the peripheral blood (Red dots) is less frequently detected in the KS sample. These samples are from the only patient from who PBMC and 2 skin samples were obtained.
