# Supplementary material for: Single-Cell Transcriptomic Analysis of Kaposi Sarcoma
Source: PLoS Pathog. 2025 Apr 1;21(4):e1012233. doi: 10.1371/journal.ppat.1012233 (PMC11984749; doi:10.1371/journal.ppat.1012233)
Supplement: S12 Fig — A) Reads for viral genes flanking and including the K5-K7 region of the KSHV genome were obtained from KSHV+ cells from 4 KS skin tumors with >100 positive cells for both LANA and K12 (KS6B, KS8, KS9, KS10B, all 3 samples with >2% KSHV positive cells and one additional sample), normalized against the Latency cluster, were graphed as a ratio of the values obtained from sample KS9. Organization of the KSHV genome was reproduced from Prazsak et al (PMC10542539). A composite UMAP plot of all KSHV+ cells reveals that the KS6B sample is distinct from the other samples. B) Bam files of reads mapped to the KSHV genome for 4 KS skin samples and visualized in IGV reveal that KS6B (1 of 3 samples with >2% KSHV positive cells) has an amplification of the K5-K7 region that is not seen in the other three samples. Amplification of this region has been described in 9 of 32 KS samples from a Ugandan cohort by Santiago et al. (PMID:36441790) (PDF) [file ppat.1012233.s012.pdf]

FIGURE S12A

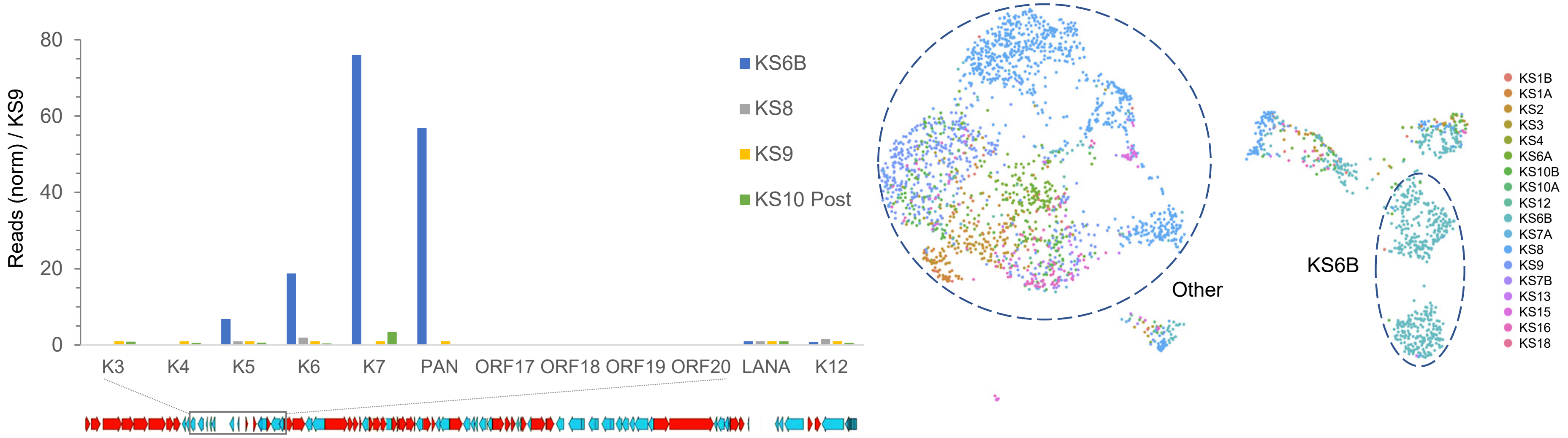

Figure S12A: Amplification of the K5-K7 region in KS6B. Reads for viral genes flanking and including the K5-K7 region of the KSHV genome were obtained from KSHV+ cells from 4 KS skin tumors with >100 positive cells for both LANA and K12 (KS6B, KS8, KS9, KS10B), normalized against the Latency cluster, were graphed as a ratio of the values obtained from sample KS9. Organization of the KSHV genome was reproduced from Prazsak et al (PMC10542539). A composite UMAP plot of all KSHV+ cells reveals that the KS6B sample is distinct from the other samples

FIGURE S12B

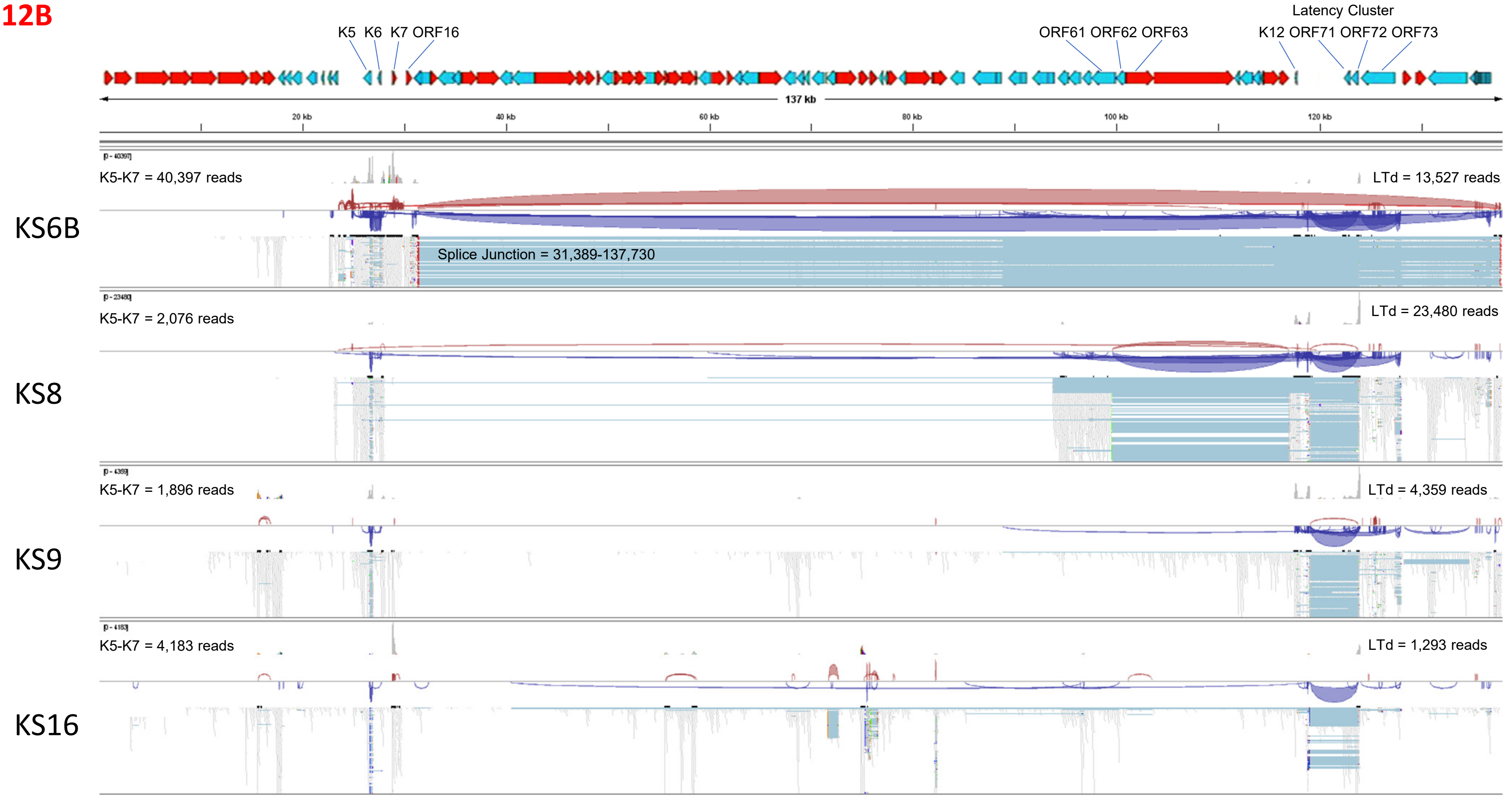

Figure S12B: Amplification of the K5-K7 region in KS6B. Bam files of reads mapped to the KSHV genome for 4 KS skin samples and visualized in IGV reveal that KS6B has an amplification of the K5-K7 region that is not seen in the other three samples. Amplification of this region has been described in 9 of 32 KS samples from a Ugandan cohort by Santiago et al. (PMID:36441790)
