# Supplementary material for: Single-Cell Transcriptomic Analysis of Kaposi Sarcoma
Source: PLoS Pathog. 2025 Apr 1;21(4):e1012233. doi: 10.1371/journal.ppat.1012233 (PMC11984749; doi:10.1371/journal.ppat.1012233)
Supplement: S13 Fig — A) Probe-capture, bulk RNAseq for KSHV latency genes ORF71 (v-FLIP), ORF72 (v-CYC), ORF73 (LANA), and ORFK12 (Kaposin) visualized on IGV. B) Digital Droplet PCR comparing DNA obtained from KS9 to three controls, i) water, ii) DNA from Jurkat cells, and iii) DNA from KSHV-expressing Vero cells showing number of positive droplets. (PDF) [file ppat.1012233.s013.pdf]

FIGURE S13

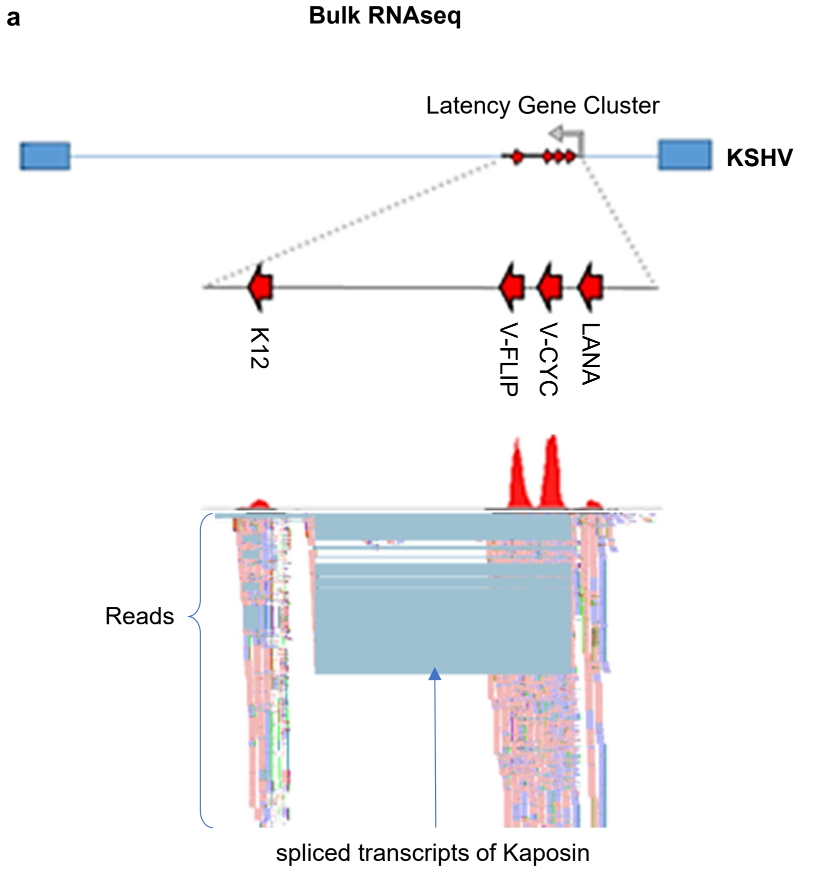

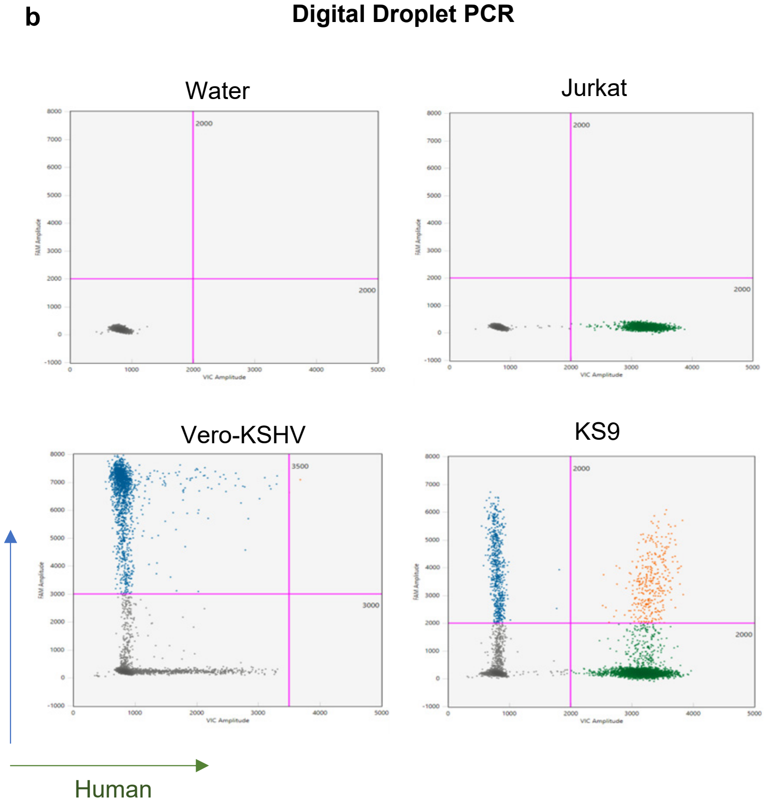

Sample	KSHV	Human RPP30	PVL (2*KSHV/hRPP30)
water	0	0	0
Jurkat	0	5240	0
Vero-KSHV	3380	0	N/A
KS9	174	1320	0.26

Figure S13: Quantitation of KSHV RNA and DNA using RNAseq and ddPCR. A) Probe-capture, bulk RNAseq for KSHV latency genes ORF71 (v-FLIP), ORF72 (v-CYC), ORF73 (LANA), and ORFK12 (Kaposin) visualized on IGV. B) Digital Droplet PCR comparing DNA obtained from KS9 to three controls, i) water, ii) DNA from Jurkat cells, and iii) DNA from KSHV-expressing Vero cells showing number of positive droplets..
