# Supplementary material for: Single-Cell Transcriptomic Analysis of Kaposi Sarcoma
Source: PLoS Pathog. 2025 Apr 1;21(4):e1012233. doi: 10.1371/journal.ppat.1012233 (PMC11984749; doi:10.1371/journal.ppat.1012233)
Supplement: S14 Fig — A) Primary KS tumor changes in one patient after ART initiation. KS tumor biopsy samples were obtained from Patient KS6 before and after initiation of antiretroviral therapy. KS6A represents the pre-therapy sample, and KS6B represents the post-therapy sample. a) Quantification of significant gene expression changes in KSHV+ tumor cells. Significant expression was determined by a p-value of <0.05 of the normalized mean gene counts in KSHV+ cells relative to KSHV- cells in the tumor. All red areas represent gene upregulation, and the blue area represents gene downregulation in KS6B. The yellow area represents genes that were upregulated in KS6A and downregulated in KS6B. b) Volcano plots of gene expression profiles of KSHV+ tumor cells in KS6A and KS6B. Due to high gene counts in KS6B, genes with a fold change between -3 and +3 were omitted. c) Cell composition of KS6A and KS6B tumors. Macrophage, CD8 T cells, and KSHV+ cells are shown on the left-hand t-SNE plots. Endothelial cells are shown on the right-hand t-SNE plots. The KSHV+ endothelial cell populations are boxed in red and the KSHV- endothelial cell population is boxed in blue. d) Volcano plot of T cell gene expression changes before and after therapy. The X axis represents the ratio of differential gene expression in T cells relative to all other tumor cells. The Y axis represents the p-value of KS6B fold change in logarithmic scale. B) Primary KS tumor changes in one patient after Nivolumab plus Ipilimumab treatment. KS tumor biopsy samples were obtained from Patient KS1 before and after a course of Nivolumab plus Ipilimumab therapy. KS1A represents the pre-therapy sample, and KS1B represents the post-therapy sample. a) Quantification of significant gene expression changes in KSHV+ tumor cells. Significant expression was determined by a p-value of <0.05 of the normalized mean gene counts in KSHV+ cells relative to KSHV- cells in the tumor. All genes represented in the diagram are upregulated in KSHV+ tumor [file ppat.1012233.s014.pdf]

FIGURE S14A

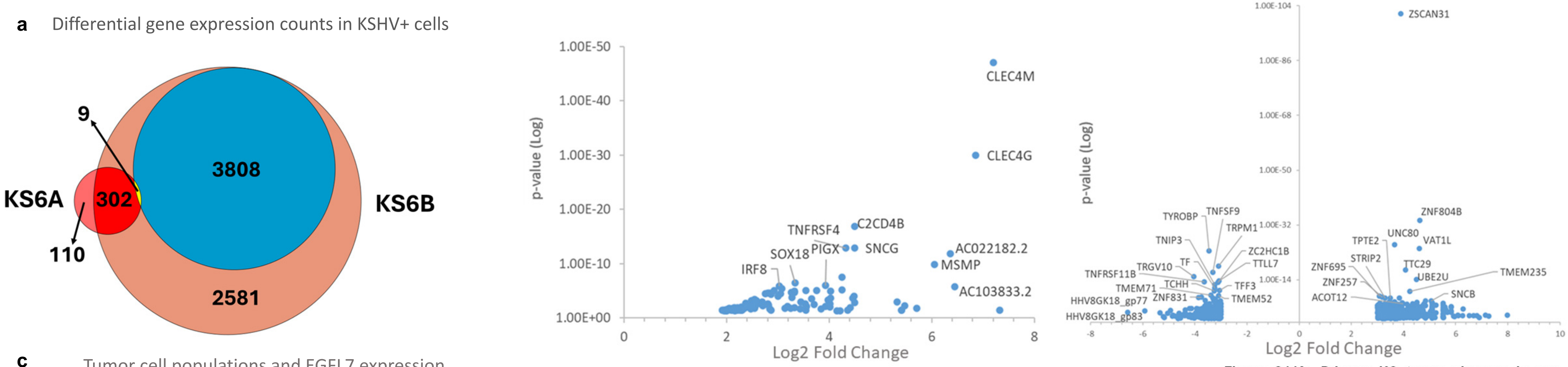

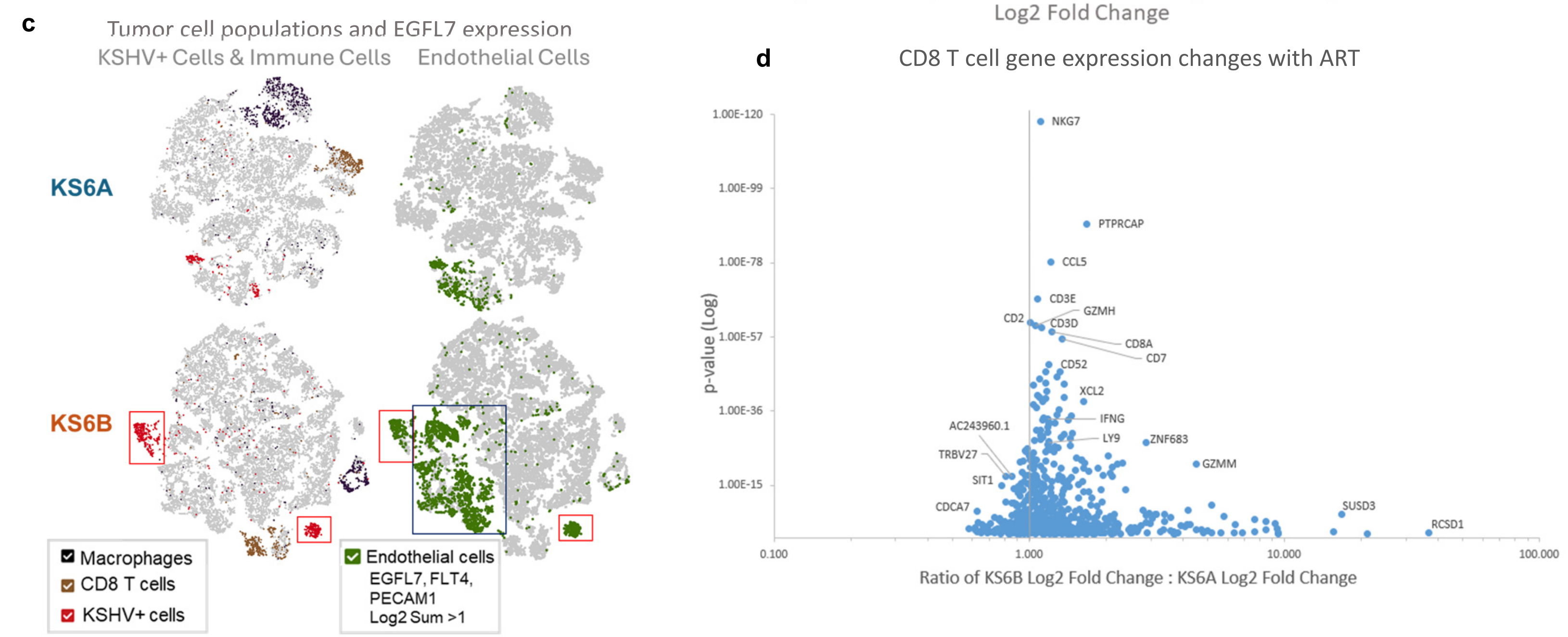

Figure S14A: Primary KS tumor changes in one patient after ART initiation. KS tumor biopsy samples were obtained from Patient KS6 before and after initiation of antiretroviral therapy. KS6A represents the pre-therapy sample, and KS6B represents the post-therapy sample. A) Quantification of significant gene expression changes in KSHV+ tumor cells. Significant expression was determined by a p-value of <0.05 of the normalized mean gene counts in KSHV+ cells relative to KSHV- cells in the tumor. All red areas represent gene upregulation, and the blue area represents gene downregulation in KS6B. The yellow area represents genes that were upregulated in KS6A and downregulated in KS6B. B) Volcano plots of gene expression profiles of KSHV+ tumor cells in KS6A and KS6B. Due to high gene counts in KS6B, genes with a fold change between -3 and +3 were omitted. C) Cell composition of KS6A and KS6B tumors. Macrophage, CD8 T cells, and KSHV+ cells are shown on the left-hand t-SNE plots. Endothelial cells are shown on the right-hand t-SNE plots. The KSHV+ endothelial cell populations are boxed in red and the KSHV- endothelial cell population is boxed in blue. D) Volcano plot of T cell gene expression changes before and after therapy. The X axis represents the ratio of differential gene expression in T cells relative to all other tumor cells. The Y axis represents the p-value of KS6B fold change in logarithmic scale.

FIGURE S14B

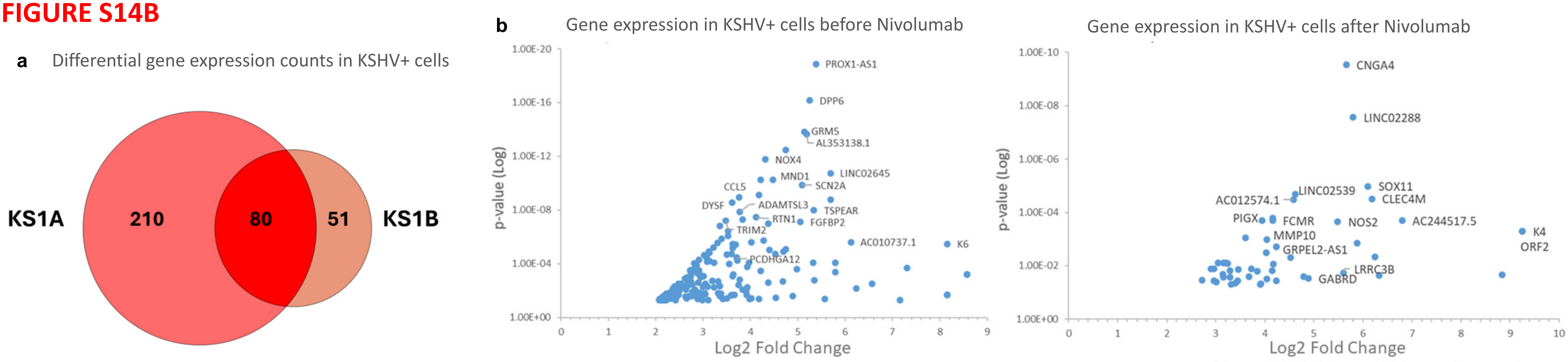

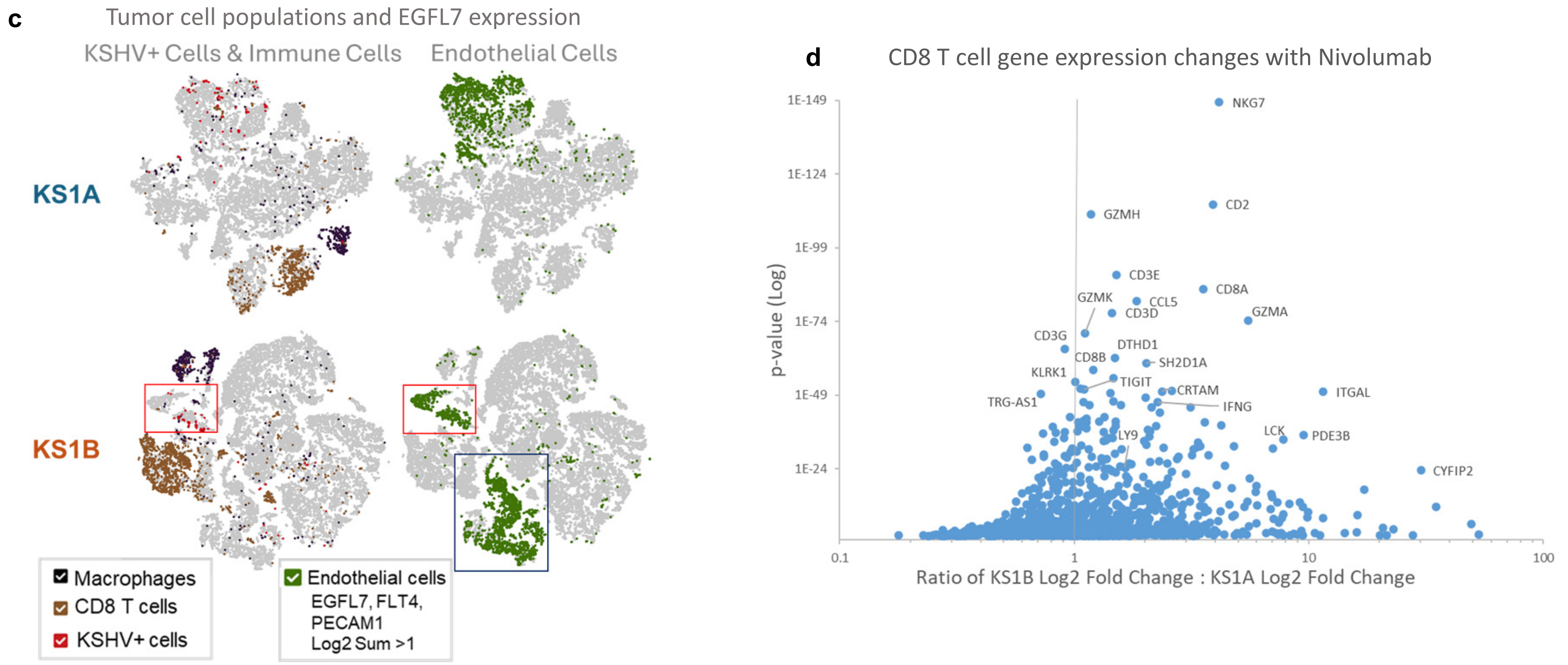

Figure S14B: Primary KS *tumor changes in one* patient after Nivolumab Plus Ipilimumab treatment. KS tumor biopsy samples were obtained from Patient KS1 before and after a course of Nivolumab plus Ipilimumab therapy. KS1A represents the pre-therapy sample, and KS1B represents the post-therapy sample. A) Quantification of significant gene expression changes in KSHV+ tumor cells. Significant expression was determined by a p-value of <0.05 of the normalized mean gene counts in KSHV+ cells relative to KSHV- cells in the tumor. All genes represented in the diagram are upregulated in KSHV+ tumor cells. B) Volcano plots of gene expression profiles of KSHV+ tumor cells in KS1A and KS1B. C) Cell composition of KS1A and KS1B tumors. Macrophage, CD8 T cells, and KSHV+ cells are shown on the left-hand t-SNE plots. Endothelial cells are shown on the right-hand t-SNE plots. The KSHV+ endothelial cell populations are boxed in red and the KSHV- endothelial cell population is boxed in blue. D) Volcano plot of the T cell gene expression changes before and after therapy. The X axis represents the ratio of differential gene expression in T cells relative to all other tumor cells. The Y axis represents the p-value of KS1B fold change in logarithmic scale.

FIGURE S14C

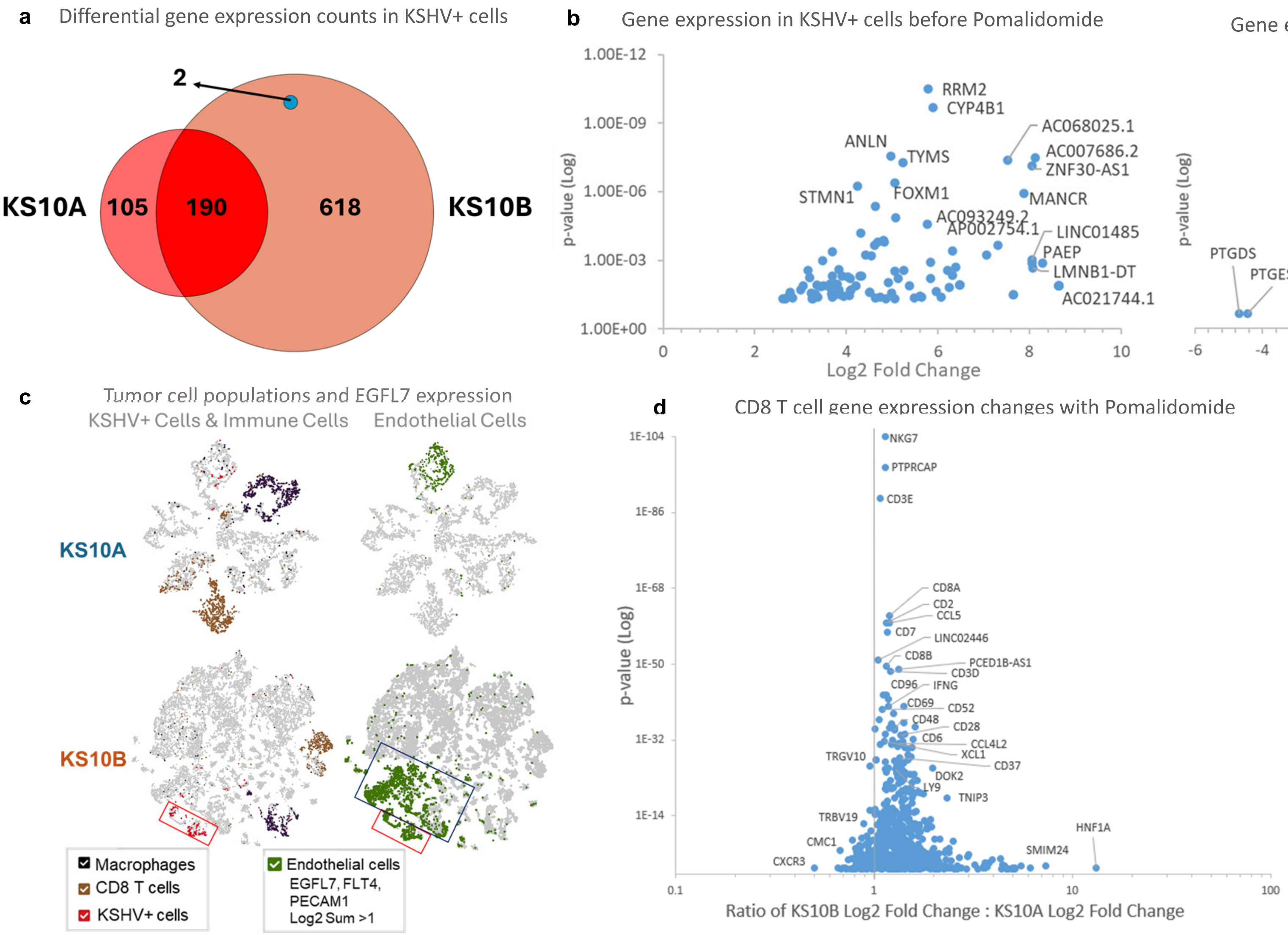

Figure S14C: Primary KS tumor changes in one patient after Pomalidomide treatment. KS tumor biopsy samples were obtained from Patient KS10 before and after initiation of pomalidomide therapy. KS10A represents the pre-therapy sample, and KS10B represents the post-therapy sample. A) Quantification of significant gene expression changes in KSHV+ tumor cells. Significant expression was determined by a p-value of <0.05 of the normalized mean gene counts in KSHV+ cells relative to KSHV- cells in the tumor. All red areas represent gene upregulation, and the blue area represents 2 downregulated genes in KS10B. B) Volcano plots of gene expression profiles of KSHV+ tumor cells in KS10A and KS10B. C) Cell composition of KS10A and KS10B tumors. Macrophage, CD8 T cells, and KSHV+ cells are shown on the left-hand t-SNE plots. Endothelial cells are shown on the right-hand t-SNE plots. The KSHV+ endothelial cell populations are boxed in red and the KSHV- endothelial cell population is boxed in blue. D) Volcano plot of the T cell gene expression changes before and after therapy. The X axis represents the ratio of differential gene expression in T cells relative to all other tumor cells. The Y axis represents the p-value of KS10B fold change in logarithmic scale. Larsson J (2021). *eulerr: Area-Proportional Euler and Venn Diagrams with Ellipses*. R package version 6.1.1, https://CRAN.R-project.org/package=eulerr.
