# Supplementary material for: Single-Cell Transcriptomic Analysis of Kaposi Sarcoma
Source: PLoS Pathog. 2025 Apr 1;21(4):e1012233. doi: 10.1371/journal.ppat.1012233 (PMC11984749; doi:10.1371/journal.ppat.1012233)
Supplement: S1 Table — Most common shared motifs in T cell receptor data from 4 PBMC samples. T cell clones were clustered based on sequence motifs likely to have a common antigen specificity using GLIPH2 software, returning 289 motif patterns (Fig 7B). The 10 most commonly shared clonal groups, listed here, are defined as those that included clones from at least 3 of the 4 PBMC samples. These data reflect the frequent use of the TRBJ1-1 segment in KS that we reported on Fig 7C. (PDF) [file ppat.1012233.s015.pdf]

Supplemental Table 1. Most Common T Cell Receptor CDR3 Motif-Based Clonal Groups Found in KS PBMC Samples.

Motif pattern[1]	Fisher score[2]	Final score[3]	Number subjects[4]	Clonal expansion[5]	V segment	J segment	TCRβ	TCRα	Sample	Freq[6]
**S%DRNTE**	1E-05	1.2E-06	4	1	TRBV5-8	**TRBJ1-1**	CASSLDRNTEAFF	CLVGDIRNNAGNMLTF	KS11	1
					TRBV7-7	**TRBJ1-1**	CASSLDRNTEAFF	CAAQGGSEKLVF	KS6B	1
					TRBV7-6	**TRBJ1-1**	CASSVDRNTEAFF	CAVSGVNTGNQFYF	KS12	1
					TRBV5-6	**TRBJ1-1**	CASSADRNTEAFF	CGIHGGTSYGKLTF	KS10	1
					TRBV18	**TRBJ1-1**	CASSPDRNTEAFF	CAGQPRDAGGTSYGKLTF	KS10	1
PLSD	2E-06	1.6E-07	3	0.044	TRBV6-2	TRBJ2-3	CASFSIVDPLSDTQYF	CVVSDGYGGATNKLIF	KS11	26
					TRBV3-1	TRBJ2-1	CASSPFIGPLSDEQFF	CAVSGDQAGTALIF	KS10	1
					TRBV2	TRBJ1-5	CASSEGPLSDQPQHF	CAVKGTSGTYKYIF	KS10	1
					TRBV6-1	TRBJ2-6	CASSEGQPLSDVLTF	CAASRSGTASKLTF	KS12	1
QGGF	2E-06	8.4E-06	3	0.22	TRBV3-1	TRBJ2-7	CASSQAGGQGGFPYEQYF	CFNTGTASKLTF	KS11	1
					TRBV7-9	TRBJ2-3	CASSLGQGGFTDTQYF	CAGQPYSGNTPLVF	KS6B	1
					TRBV3-1	TRBJ1-3	CASSQEGQGGFFGNTIYF	CALSALRWTGNNRKLIW	KS6B	4
					TRBV4-1	TRBJ2-3	CASSQDIRQGGFTDTQYF	CALSEPRTGNQFYF	KS6B	1
					TRBV11-2	TRBJ1-6	CASSLDQGGFYNSPLHF	CAVEKSNNNARLMF	KS12	1
SLGG%NTE	2E-05	1.3E-07	3	0.52	TRBV5-6	**TRBJ1-1**	CASSLGGINTEAFF	CAVDPAGSNYQLIW	KS6B	2
					TRBV5-6	**TRBJ1-1**	CASSLGGINTEAFF	CVVSDSGYSTLTF	KS6B	1
					TRBV5-1	**TRBJ1-1**	CASSLGGGNTEAFF	CALSDRNSGYALNF	KS11	1
					TRBV13	**TRBJ1-1**	CASSLGGENTEAFF	CAAGGDTGRRALTF	KS10	1
					TRBV28	**TRBJ1-1**	CASSLGGRNTEAFF	CAVRPLYNQGGKLIF	KS6B	1
SSQG%E	0.0007	1.1E-06	3	1	TRBV27	TRBJ2-7	CASSSQGYEQYF	CAVRDQNTGFQKLVF	KS6B	1
					TRBV5-1	TRBJ1-1	CASSSQGTEAFF	CAASRSGNYGGSQGNLIF	KS10	1
					TRBV7-9	TRBJ2-7	CASSSQGREQYF	CAVRRGSGGGADGLTF	KS12	1
%LDRNTE	0.0014	5.4E-06	3	1	TRBV29-1	**TRBJ1-1**	CSVYLDRNTEAFF	CAGRRKTSYDKVIF	KS10	1
					TRBV5-8	**TRBJ1-1**	CASSLDRNTEAFF	CLVGDIRNNAGNMLTF	KS11	1
					TRBV7-7	**TRBJ1-1**	CASSLDRNTEAFF	CAAQGGSEKLVF	KS6B	1
S%GGYE	0.0021	3.7E-07	3	0.33	TRBV25-1	TRBJ2-1	CASSGGGYEQFF	CAVKAGGYQKVTF	KS10	1
					TRBV12-3	TRBJ2-7	CASSLGGYEQYF	CAVSRYNDYKLSF	KS12	1
					TRBV6-5	**TRBJ1-1**	CASSIGGYEAFF	CASGGGADGLTF	KS6B	2
S%GGTGE	0.0022	1.1E-06	3	1	TRBV7-3	TRBJ2-1	CASSLGGTGEQFF	CAGQPRNDYKLSF	KS10	1
					TRBV11-3	TRBJ2-2	CASSLGGTGELFF	CAMKAGNKLTF	KS11	1
					TRBV2	TRBJ2-2	CASSGGGTGELFF	CALGPSSNTGKLIF	KS12	1
S%GGTE	0.0027	9.8E-08	3	0.44	TRBV25-1	**TRBJ1-1**	CASSVGGTEAFF	CARTPLHAGGTSYGKLTF	KS12	2
					TRBV25-1	**TRBJ1-1**	CASSVGGTEAFF	CAVRDSKAPKGAGGSYIPTF	KS12	1
					TRBV7-2	**TRBJ1-1**	CASSTGGTEAFF	CAAPNTDSWGKLQF	KS10	1
					TRBV7-9	**TRBJ1-1**	CASSRGGTEAFF	CAFMSINDMRF	KS6B	1
S%GDE	0.0056	5.6E-11	3	0.003	TRBV25-1	TRBJ2-7	CASSPGDEQYF	CALKTSYDKVIF	KS11	44
					TRBV3-1	TRBJ2-1	CASSQGDEQFF	CAAPLGTSYGKLTF	KS10	1
					TRBV25-1	TRBJ2-1	CASSPGDEQFF	CALKTGANNLFF	KS12	18
					TRBV25-1	TRBJ2-1	CASSPGDEQFF	CAVKTPADKLIF	KS12	1
					TRBV25-1	TRBJ2-1	CASSPGDEQFF	CAVKTPTDKLIF	KS12	47

T cell receptor data for 4 PBMC samples were clustered based on sequence motifs likely to have a common antigen specificity using GLIPH2 software. The most commonly shared motifs among samples, defined as those shared by at least 3 samples, are listed in this table.

Use of TRBJ1-1, one of most common TRBJ segments found in our samples, in 5 of the 10 most common CDR3 motifs is marked in bold in the 'J segment' column.

[1]Motif pattern: amino acid sequence that defines the clonal group. % indicates a variable residue. Bolded motifs indicate the clonal group includes clones from all 4 samples. Underlined motifs indicate the clonal group includes clones from the 3 AIDS-associated/epidemic KS subjects, but not from the iatrogenic KS subject.

[2]Fisher score: significance score, calculated by Fisher exact test.

[3]Final score: calculation of overall significance of the GLIPH output that combines and summarizes scores assigned to the clonal group's individual components.

[4]Number subjects: how many of the 4 PBMC samples are represented in the clonal group.

[5]Clonal expansion: score of clonal expansion enrichment generated by the GLIPH2 output.

[6]Freq: count of cells featuring a specific clonotype.

Reference: Huang, Huang, et al. "Analyzing the Mycobacterium tuberculosis immune response by T-cell receptor clustering with GLIPH2 and genome-wide antigen screening." Nature Biotechnology 38.10 (2020): 1194-1202. http://50.255.35.37:8080/
