# Supplementary material for: Single-Cell Transcriptomic Analysis of Kaposi Sarcoma
Source: PLoS Pathog. 2025 Apr 1;21(4):e1012233. doi: 10.1371/journal.ppat.1012233 (PMC11984749; doi:10.1371/journal.ppat.1012233)
Supplement: S1 Methods — (PDF) [file ppat.1012233.s016.pdf]

Supplemental Methods

Probes for Probe Capture Bulk RNAseq

ORF73 (LANA): Probe is within conserved LANA C-Terminal domain

TTATCTCAGGCCTTCCAGTTTGGAGGAGTAAAGGCAGGCCCCGTGTCCTGCTTGCCCCAC
CCTGGACCAGACCAGTCGCCCATAACTTATTGTGTATATGTGTATTGTCAGAACAAAGAC

ORF72 (v-CYC): Probe is within conserved Cyclin N-terminal domain; aa 21-145

TTCGCATATGCGTAAGTTACTGGGCACATGGATGTTTTCAGTTTGCCAGGAATACAACCTA
GAACCTAACGTGGTCGCGTTGGCCCTTAATCTTTTGGACAGACTCCTACTTATAAAGCA

ORF71 (v-FLIP): Probe is within conserved DED_Caspase-like_r1; Death effector domain, repeat 1, of initiator caspase-like proteins N-terminal domain; aa 3-64)

TACGAGGTTCTCTGTGAGGTGGCGCGGAAACTGGGCACGGATGACAGGGAAGTGGTATT
GTTCCTCCTAAACGTGTTCATACCTCAACCCACACTGGCCCAATTAATTGGAGCTCTTAGA

ORFK12 (Kaposin) Probe corresponds to aa 1-40 of Kaposin A)

ATGGATAGAGGCTTAACGGTGTTTGTGGCAGTTCATGTCCCGGATGTGTTACTAAATGGGT
GGCGCTGGAGGCTTGGGGCGATACCACCACTCGTTTGTCTGTTGGCGATTAGTGTTGTC
